# Supplementary material for: Symmetry breaking meets multisite modification
Source: eLife. 2021 May 21;10:e65358. doi: 10.7554/eLife.65358 (PMC8439660; doi:10.7554/eLife.65358)
Supplement: Supplementary file 1. [file elife-65358-supp1.pdf]

Symmetry breaking meets multisite modification - Source code file

Vaidhiswaran Ramesh[1], J Krishnan[1,2]

Affiliations:
1 - Department of Chemical Engineering, Centre for Process Systems Engineering, Imperial College London, London, SW7 2AZ, UK
2 - Institute for Systems and Synthetic Biology, Imperial College London, South Kensington Campus, London SW7 2AZ, UK

Analytical proofs exploring the presence and features of symmetry and symmetry breaking in various multisite phosphorylation networks

This text details the analytical arguments used to ascertain features associated with symmetry breaking and the impossibility of the behavior in various MSP networks considered in the main text.

Models
The networks described in the main text are modelled as a system of ODEs (Refer to Fig 1 in the main text for reaction scfhematics and Appendix 2 Figure 10 for kinetic nomenclature of elementary reactions). We use mass action kinetic description for reaction rates and the overall model is constructed as a combination of such descriptions for the elementary reactions.

Cross Validation and Parameters
The models provided here in Maple have been analyzed and cross validated with results from Matlab simulations and bifurcation computations using the Matlab package MatCont. Matlab and bifurcation computations have in turn been cross validated with simulations from the software COPASI, which self generates the ODE expressions based on a reaction schematic. The parameters used for generating the figures are presented here (in addition to the being present in appendix 2). These parameters have been used to perform an additional cross validation of the analytical work by mapping the presence and features of symmetry breaking, as predicted by the analytical work and obtained in the computational analysis.

Organization of Results
The general organization of this Maple workbook is as follows. There are a total of 10 maple worksheets organized across 5 folders.

Read_Me (This file) : Contains the parameters used for generating the figures, and further cross validation of the invariants and necessary conditions predicted by the analytical work.

Folder 2: Ordered distributive double site phosphorylation (DSP) models and triple site phosphorylation (TSP) (2.1 - 2.3)
Folder 3: Random DSP (3.1 - 3.3)
Folder 4: Mixed-Random DSP (4.1 - 4.3)
Folder 5: Absolute Concentration Robustness (5.1)

Each of the folders contain the discussions and analytical work pertaining to the various models in that category. For example, in Random folder: The three different models, corresponding to common kinase-common phosphatase, separate kinase-common phosphatase and separate kinase-separate phosphatase enzyme configurations, are presented in separate Maple documents labelled System_1, System_2 and System_3. Within each of these models, the three different classes of symmetries, where applicable are presented.

Necessary and Sufficient Conditions.
In this supplementary material, we provide analytical arguments regarding the presence and absence of various cases of symmetry breaking in multiple DSP networks. In networks permitting symmetry breaking, we obtain a necessary and sufficient condition for the behavior to manifest at some total substrate concentrations, in terms of the kinetic parameters (and in some cases total enzyme concentrations).

These constraints act as sufficient conditions to ensure symmetry breaking behavior is seen for some finite value of total substrate concentration (A_{Total}). The choice of bifurcation along A_{Total} is due to its flexibility in accommodating different classes of symmetry while changing. However the symmetry breaking can be encountered upon bifurcation along any parameter of interest (kinetic or enzyme concentrations) provided there is sufficient total substrate concentration.

Please note: This entire document is also presented in a PDF format and is available as Supplementary file 1.

Parameters

The parameters used in generating the figures are presented below. The kinetic nomenclature pertaining to the specific system and class of symmetry is used below (refer main text, Fig 1D). As discussed earlier the bifurcation is performed along A_{Total} in these figures. A cross verification of the analytical results showing that the necessary conditions for symmetry breaking are met is presented. The features of the symmetry broken state as predicted by the analytical work is also presented.

Figure 2

A. Case 1 - Double site phosphorylation

restart :

$k_1 := 0.1 : k_{b1} := 1 : k_{ub1} := 1 :$
$k_2 := 0.5 : k_{b2} := 1 : k_{ub2} := 1 :$

$P_{Total} := 0.1 :$

$$c_1 := \frac{k_{b1}}{k_1 + k_{ub1}} : c_2 := \frac{k_{b2}}{k_2 + k_{ub2}} :$$

Necessary and sufficient condition

Here we show that the necessary and sufficient condition for symmetry breaking (refer to analytical work) is satisfied for the parameters used.

1. $k_2 > k_1$

$$0.1 < 0.5 \quad \textbf{(1.1.1.1)}$$

Symmetry Breaking (Invariants and position of symmetry in the bifurcation diagram along A_{Total})

The invariant in the asymmetric steady states in ordered distributive DSP post case 1 symmetry breaking is the concentration of the partially modified substrate [Ap].
This is evaluated (based on the expression from the analytical work) for the kinetic parameters used in generating the plot.

$$Ap = -\frac{k_1}{c_2\,(k_1 - k_2)}$$

$$Ap = 0.3750000000 \quad \textbf{(1.1.2.1)}$$

The intersection of the symmetric and asymmetric steady states on the bifurcation plot indicates the presence of a pitch fork bifurcation.
Below, the position of this pitch fork bifurcation is evaluated (based on the expression from the analytical work) for the kinetic parameters used in generating the plot.

$$A_{Total} = \frac{\left(-c_1 c_2 P_{Total} - 2 c_2\right) k_2^{\,2} - k_1 c_1 k_2 + P_{Total} k_1^{\,2} c_1 c_2}{\left(k_1 - k_2\right) c_1 c_2 k_2}$$

$$A_{Total} = 3.245000000 \qquad \textbf{(1.1.2.2)}$$

B. Case 1 - System 1 Random DSP

restart :

$k_1 := 0.25 : k_{b1} := 1 : k_{ub1} := 1 :$
$k_2 := 0.4 : k_{b2} := 1 : k_{ub2} := 1 :$

$a_1 := 0.1 : a_{b1} := 1 : a_{ub1} := 1 :$
$a_2 := 1 : a_{b2} := 1 : a_{ub2} := 1 :$

$P_{Total} := 1 :$

$$c_1 := \frac{k_{b1}}{k_1 + k_{ub1}} : c_2 := \frac{k_{b2}}{k_2 + k_{ub2}} :$$

$$d_1 := \frac{a_{b1}}{a_1 + a_{ub1}} : d_2 := \frac{a_{b2}}{a_2 + a_{ub2}} :$$

Necessary Constraint

Here we show that the necessary and sufficient condition for symmetry breaking (refer to analytical work) is satisfied for the parameters used.

1. $c_1\, a_2 \left(k_2 - k_1\right) + d_1 k_2 \left(a_2 - a_1\right) > 0$

$$0 < 1.163636364 \qquad \textbf{(1.2.1.1)}$$

Symmetry Breaking (Invariants and position of symmetry in the bifurcation diagram along A_{Total}

The invariant in the asymmetric steady states in System 1 Random ordered DSP post case 1 symmetry breaking is the concentration of the partially modified substrates [A01] and [A10]. This is evaluated (based on the expression from the analytical work) for the kinetic parameters used in generating the plot.

$$A01 := -\frac{c_1 k_1 a_2}{c_2 \left(\left(\left(k_1 - k_2\right) c_1 - d_1 k_2\right) a_2 + d_1 k_2 a_1\right)}$$

$$A01 := 0.6260162603 \quad \textbf{(1.2.2.1)}$$

$$A10 := -\frac{d_1 k_2 a_1}{d_2 \left(\left(d_1 \left(a_1 - a_2\right) - c_1 a_2\right) k_2 + c_1 k_1 a_2\right)}$$

$$A10 := 0.1626016260 \quad \textbf{(1.2.2.2)}$$

The intersection of the symmetric and asymmetric steady states on the bifurcation plot indicates the presence of a pitch fork bifurcation.
Below, the position of this pitch fork bifurcation is evaluated (based on the expression from the analytical work) for the kinetic parameters used in generating the plot.

$$A_{Total} = \left(d_2 \left(-c_2 \left(c_1 + d_1\right) \left(c_1 P_{Total} + d_1 P_{Total} + 2\right) k_2^2 - c_1 k_1 \left(c_1 + d_1\right) k_2 + c_1^2 c_2 k_1^2 P_{Total}\right) a_2^2 + 2 d_1 \left(\left(-\frac{c_1}{2} - \frac{d_1}{2}\right) k_2 + c_1 d_2 k_1 P_{Total}\right) c_2 k_2 a_1 a_2 + a_1^2 c_2 d_1^2 d_2 k_2^2 P_{Total}\right) \Big/ \left(\left(\left(\left(-c_1 - d_1\right) k_2 + c_1 k_1\right) a_2 + a_1 d_1 k_2\right) a_2 \left(c_1 + d_1\right) c_2 d_2 k_2\right)$$

$$A_{Total} = 3.922980455 \quad \textbf{(1.2.2.3)}$$

C. Case 1 - System 3 Random DSP

restart :

$k_1 := 0.5 : k_{b1} := 1 : k_{ub1} := 1 :$
$k_2 := 1.5 : k_{b2} := 1 : k_{ub2} := 1 :$

$a_1 := 0.1 : a_{b1} := 1 : a_{ub1} := 1 :$
$a_2 := 1 : a_{b2} := 1 : a_{ub2} := 1 :$

$P1_{Total} := 1 :$
$P2_{Total} := 1 :$

$$c_1 := \frac{k_{b1}}{k_1 + k_{ub1}} : c_2 := \frac{k_{b2}}{k_2 + k_{ub2}} :$$

$$d_1 := \frac{a_{b1}}{a_1 + a_{ub1}} : d_2 := \frac{a_{b2}}{a_2 + a_{ub2}} :$$

Necessary Constraint

Here we show that the necessary and sufficient conditions for symmetry breaking (refer to

analytical work) is satisfied for the parameters used.

1. $k_2 \cdot P1_{Total} > k_1 \cdot P2_{Total}$

$$0.5 < 1.5 \quad (1.3.1.1)$$

2. $a_2 \cdot P2_{Total} > a_1 \cdot P1_{Total}$

$$0.1 < 2. \quad (1.3.1.2)$$

Symmetry Breaking (Invariants and position of symmetry in the bifurcation diagram along A_{Total})

The invariant in the asymmetric steady states in System 3 Random ordered DSP post case 1 symmetry breaking is the concentration of the partially modified substrates [A01] and [A10]. This is evaluated (based on the expression from the analytical work) for the kinetic parameters used in generating the plot.

$$A01 := -\frac{k_1\, P2_{Total}}{c_2\left(k_1\, P2_{Total} - k_2\, P1_{Total}\right)}$$

$$A01 := 1.250000000 \quad (1.3.2.1)$$

$$A10 := -\frac{a_1\, P1_{Total}}{d_2\left(a_1\, P1_{Total} - a_2\, P2_{Total}\right)}$$

$$A10 := 0.2222222222 \quad (1.3.2.2)$$

The intersection of the symmetric and asymmetric steady states on the bifurcation plot indicates the presence of a pitch fork bifurcation.

First we evaluate the value of [K2] and [P2] using the various correlations between the substrates and free enzyme concentrations at steady state. Below we use the *solve* command in Maple to evaluate the value for [K2] from the correlation obtained between [A01] and [A10] earlier. We use additional arguments (use assumptions) to only obtain solutions for [K2] that permit positive concentrations of free enzyme concentrations.

$$K2 := solve\left(A10 = \frac{A01\, K2^2\, a_1\, c_2\, d_1\, k_2}{P2^2\, c_1\, k_1\, a_2\, d_2}, K2, useassumptions\right) \text{assuming } P2 > 0, K2 > 0$$

$$K2 := 0.7370277311\, P2 \quad (1.3.2.3)$$

This gives us the value of α (the ratio of [K2]/[P2]). Using this in the expression for [P2] obtained earlier from solving P2Con at the symmetric state, we get the concentration of [P2] to be,

$$P2 := eval\left(-\frac{\left(a_1\, P1_{Total} - a_2\, P2_{Total}\right)\left(k_1\, P2_{Total} - k_2\, P1_{Total}\right)}{k_2\left(a_1\, \alpha - a_2\right) P1_{Total} + a_2\, P2_{Total}\left(-\alpha\, k_2 + k_1\right)}, \alpha = 1.356801051\right)$$

$$P2 := 0.3178323642 \quad \textbf{(1.3.2.4)}$$

$$A11 := \frac{K2\ A01\ c_2\ k_2}{P2\ c_1\ k_1}$$

$$A11 := 1.658312396 \quad \textbf{(1.3.2.5)}$$

$$A00 := \frac{K2\ A01\ c_2\ k_2}{P2\ c_1\ k_1}$$

$$A00 := 1.658312396 \quad \textbf{(1.3.2.6)}$$

Using these values, the position of the pitch fork bifurcation is evaluated as shown below.

$$A_{Total} = \left(- \left(\left(-2\ a_2 \left(\left(P2\ (k_2 + k_1)\ c_1 + k_2\ (K2\ d_1 + 1) \right) K2\ c_2 + \frac{P2\ c_1\ k_1}{2} \right) d_2\ P2_{Total}^{\ 2} + 2\ P1_{Total} \left(\left(\left(P2\ \left(k_1\ P2 + K2\ (k_2 + k_1)\right) c_1 + K2\ k_2\ (K2\ d_1 + 1) \right) d_2 + \frac{P2\ c_1\ k_1}{2} \right) c_2 + \frac{P2\ c_1\ d_2\ k_1}{2} \right) a_1\ P2_{Total} - 2\ P1_{Total}^{\ 2}\ P2\ c_1 \left(P2\ d_2 + \frac{1}{2} \right) a_1\ k_2\ c_2 \right) \right) \Big/ \left(\left(\left(k_1\ P2_{Total} - k_2\ P1_{Total}\right) c_1\ P2\ d_2\ \left(a_1\ P1_{Total} - a_2\ P2_{Total}\right) c_2 \right) \right)$$

$$A_{Total} = 6.502775632 \quad \textbf{(1.3.2.7)}$$

D. Case 2 - System 3 Random DSP

restart :

$k_1 := 2.35 : k_{b1} := 1 : k_{ub1} := 1 :$
$k_2 := 0.46 : k_{b2} := 1 : k_{ub2} := 1 :$
$k_3 := 1.86 : k_{b3} := 1 : k_{ub3} := 1 :$
$k_4 := 1.1 : k_{b4} := 1 : k_{ub4} := 1 :$

$P_{Total} := 1 :$
$K_{Total} := 1 :$

$$c_1 := \frac{k_{b1}}{k_1 + k_{ub1}} : c_2 := \frac{k_{b2}}{k_2 + k_{ub2}} : c_3 := \frac{k_{b3}}{k_3 + k_{ub3}} : c_4 := \frac{k_{b4}}{k_4 + k_{ub4}} :$$

Necessary Conditions

Here we show that the necessary and sufficient conditions for symmetry breaking (refer to analytical work) is satisfied for the parameters used.

1. $k_1 \cdot K_{Total} > k_4 \cdot P_{Total}$

$$1.1 < 2.35 \quad \textbf{(1.4.1.1)}$$

2. $k_3 \cdot P_{Total} > k_2 \cdot K_{Total}$

$$0.46 < 3.72 \quad \textbf{(1.4.1.2)}$$

Symmetry Breaking (Invariants and position of symmetry in the bifurcation diagram along A_{Total})

The invariant in the asymmetric steady states in System 3 Random ordered DSP post case 2 symmetry breaking is the concentration of the partially modified substrate [A00] and [A11]. This is evaluated (based on the expression from the analytical work) for the kinetic parameters used in generating the plot.

$$A00 := \frac{k_4 P_{Total}}{c_1 \left(k_1 K_{Total} - k_4 P_{Total}\right)}$$

$$A00 := 2.948000000 \quad \textbf{(1.4.2.1)}$$

$$A11 := -\frac{k_2 K_{Total}}{c_3 \left(k_2 K_{Total} - k_3 P_{Total}\right)}$$

$$A11 := 0.9397142857 \quad \textbf{(1.4.2.2)}$$

The intersection of the symmetric and asymmetric steady states on the bifurcation plot indicates the presence of a pitch fork bifurcation.

First we evaluate the value of [K2] and [P2] using the various correlations between the substrates and free enzyme concentrations at steady state. Below we use the *solve* command in Maple to evaluate the value for [K2] from the correlation obtained between [A01] and [A10] earlier. We use additional arguments (use assumptions) to only obtain solutions for [K2] that permit positive concentrations of free enzyme concentrations.

$$K2 := solve\left(A11 = \frac{A00\, K2^2\, c_1\, c_2\, k_1\, k_2}{P2^2\, c_4\, k_4\, c_3\, k_3}, K2, useassumptions\right) \text{assuming } P2 > 0, K2 > 0$$

$$K2 := 0.7009391379\, P2 \quad \textbf{(1.4.2.3)}$$

This gives us the value of ϵ (the ratio of [K2]/[P2]). Using this in the expression for [P2] obtained earlier from solving P2Con at the symmetric state, we get the concentration of [P2] to be,

$$P2 := eval\left(\frac{\left(k_2 K_{Total} - k_3 P_{Total}\right)\left(k_1 K_{Total} - P_{Total} k_4\right)}{k_1 \left(\epsilon k_2 - k_3\right) K_{Total} - k_3 P_{Total} \left(\epsilon k_1 - k_4\right)}, \epsilon = 0.7009391379\right)$$

$$P2 := 0.3778808204 \quad \textbf{(1.4.2.4)}$$

$$A01 := \frac{K2\, A00\, c_1\, k_1}{P2\, c_4\, k_4}$$

$$A01 := 2.767307716 \quad \textbf{(1.4.2.5)}$$

$$A10 := \frac{K2\, A00\, c_1\, k_1}{P2\, c_4\, k_4}$$

$$A10 := 2.767307716 \quad \textbf{(1.4.2.6)}$$

Using these values, the position of the pitch fork bifurcation is evaluated as shown below.

$$A_{Total} = \frac{1}{P2^2\, c_4\, k_4\, c_3\, k_3} \left(2 \left(P2 \left(\left(\left(K2\, (k_4 + k_1)\, c_1 + \frac{k_4}{2}\right) c_4\, k_3\, P2 + \left((K2\, c_2 + 1)\, k_3 + K2\, c_2\, k_2 \right) c_1\, k_1\, K2 \right) c_3 + \frac{K2^2\, c_1\, c_2\, k_1\, k_2}{2} \right) A00 \right)\right)$$

$$A_{Total} = 12.13682517 \quad \textbf{(1.4.2.7)}$$

E. Case 2 - Mixed-Random 2 DSP

restart :

$k_1 := 2 : k_{b1} := 1 : k_{ub1} := 1 :$
$k_2 := 0.1 : k_{b2} := 1 : k_{ub2} := 1 :$
$k_3 := 0.75 : k_{b3} := 1 : k_{ub3} := 1 :$
$k_4 := 1 :$

$P_{Total} := 0.2 : K1_{Total} := 0.1 :$

$$c_1 := \frac{k_{b1}}{k_1 + k_{ub1}} : c_2 := \frac{k_{b2}}{k_2 + k_{ub2}} : c_3 := \frac{k_{b3}}{k_3 + k_{ub3}} :$$

Necessary Conditions

Here we show that the necessary and sufficient condition for symmetry breaking (refer to analytical work) is satisfied for the parameters used.

1. $k_2 < k_1$

$$0.1 < 2 \quad \textbf{(1.5.1.1)}$$

2. $k_2\, K1_{Total}\, (k_3 + k_4) < P_{Total}\, k_3\, k_4$

$$0.0350 < 0.150 \quad \textbf{(1.5.1.2)}$$

Symmetry Breaking (Invariants and position of symmetry in the bifurcation diagram along A_{Total})

The invariant in the asymmetric steady states in Mixed Random 2 ordered DSP post case 2 symmetry breaking is the concentration of the partially modified substrate [A00] and [A11]. This is evaluated (based on the expression from the analytical work) for the kinetic parameters used in generating the plot.

$$A00 = \frac{k_2}{(k_1 - k_2)\, c_1}$$

$$A00 = 0.1578947368 \qquad \textbf{(1.5.2.1)}$$

$$A11 = -\frac{k_2\, k_4\, K1_{Total}}{2\, c_3\, \left(k_2\, K1_{Total}\, (k_3 + k_4) - P_{Total}\, k_3\, k_4\right)}$$

$$A11 = 0.06603773585 \qquad \textbf{(1.5.2.2)}$$

The intersection of the symmetric and asymmetric steady states on the bifurcation plot indicates the presence of a pitch fork bifurcation.
The position of the pitch fork bifurcation is evaluated (based on the expression from the analytical work) for the kinetic parameters used in generating the plot.

$$A_{Total} := \Big(-2\, c_3\, (k_3 + k_4)\, \left((k_1 + k_4)\, k_3 + k_1\, k_4\right) c_1\, c_2\, K1_{Total}^{\,2}\, k_2^{\,3} + 2\, c_2 \Big(c_3\, \left(c_1\, P_{Total}\, k_4^{\,2} + k_1\, (c_1\, P_{Total} + 1)\, k_4 + c_1\, k_1^{\,2}\, K1_{Total}\right) k_3^{\,2} + 2\, \Big(\Big(\frac{1}{2}\, c_1\, c_3\, P_{Total} + \frac{1}{4}\, c_1 + \frac{1}{2}\, c_3\Big) k_4 + c_1\, c_3\, k_1\, K1_{Total}\Big) k_4\, k_1\, k_3 + c_1\, c_3\, k_1^{\,2}\, k_4^{\,2}\, K1_{Total}\Big) K1_{Total}\, k_2^{\,2} - 2\, k_3\, \Big(c_3\, \big(c_2\, P_{Total}\, k_4 + c_1\, k_1\, K1_{Total}\, (-K1_{Total}\, c_2 + c_2\, P_{Total} - 2)\big)\, k_3 + c_1\, k_1\, k_4\, K1_{Total}\, \Big(c_2\, c_3\, P_{Total} - c_2\, c_3\, K1_{Total} + \frac{1}{2}\, c_2 - 2\, c_3\Big)\Big) k_4\, k_1\, k_2 - 2\, c_1\, c_3\, k_1^{\,2}\, k_3^{\,2}\, k_4^{\,2}\, P_{Total}\, (K1_{Total}\, c_2 + 2)\Big) \Big/ \left(2\, (k_1 - k_2)\, c_3\, k_3\, \left(k_2\, (k_3 + k_4)\, K1_{Total} - P_{Total}\, k_3\, k_4\right) k_4\, c_1\, c_2\, k_1\right)$$

$$A_{Total} := 2.668055280 \qquad \textbf{(1.5.2.3)}$$

Figure 3

A. Case 3 - System 1 Random DSP (Hopf and Pitchfork)

restart :

$k_1 := 100 : k_{b1} := 100 : k_{ub1} := 1 :$
$k_2 := 2 : k_{b2} := 1 : k_{ub2} := 1 :$
$k_3 := 0.01 : k_{b3} := 100 : k_{ub3} := 1 :$
$k_4 := 20 : k_{b4} := 0.1 : k_{ub4} := 1 :$

$P_{Total} := 1.25 :$

$$c_1 := \frac{k_{b1}}{k_1 + k_{ub1}} : c_2 := \frac{k_{b2}}{k_2 + k_{ub2}} : c_3 := \frac{k_{b3}}{k_3 + k_{ub3}} : c_4 := \frac{k_{b4}}{k_4 + k_{ub4}} :$$

Necessary conditions

Here we show that the necessary and sufficient condition for symmetry breaking (refer to analytical work) is satisfied for the parameters used.

1. $c_3 k_4 (k_2 - k_3) - c_1 k_2 (k_1 - k_4) > 0$

$$0 < 3782.178218 \quad \textbf{(2.1.1.1)}$$

Symmetry Breaking

The invariant in the asymmetric steady states in System 1 Random ordered DSP post case 3 symmetry breaking is the sum of the concentrations of the partially modified substrates [A01] and [A10].
This is evaluated (based on the expression from the analytical work) for the kinetic parameters used in generating the plot.

$$A01 + A10 = \frac{-c_1 c_2 k_1 k_2 - c_3 c_4 k_3 k_4}{\left(\left(\left(-c_1 - c_3\right) k_4 + c_1 k_1\right) k_2 + c_3 k_3 k_4\right) c_4 c_2}$$

$$A01 + A10 = 11.01047120 \quad \textbf{(2.1.2.1)}$$

The intersection of the symmetric and asymmetric steady states on the bifurcation plot indicates the presence of a pitch fork bifurcation.
Below, the position of the pitch fork bifurcation is evaluated (based on the expression from the analytical work) for the kinetic parameters used in generating the plot.

$$A_{Total} = \Bigg(2 \left(c_1 c_2 k_1 k_2 + c_3 c_4 k_3 k_4\right) \Bigg(-\frac{c_1 k_2^2 k_1 \left(\left(k_1 + k_2\right) c_1 + c_3 \left(k_2 + k_3\right)\right) c_2^3}{2}$$
$$+ \Bigg(\Bigg(k_1 k_2 P_{Total} \left(k_1 - k_4\right) \left(k_1 + k_2\right) c_1^3 + \Bigg(\left(\left(\left(-2 k_1 - k_3\right) k_4 + k_1 \left(k_1 + k_3\right)\right) k_2^2$$

$$+\left(-k_1\left(k_1+k_3\right)k_4+2k_1^2k_3\right)k_2+k_1^2k_3k_4\right)P_{Total}c_3$$

$$+\frac{k_2\left(\left(k_1-4k_4\right)k_2-k_4k_1\right)k_1}{2}\Bigg)c_1^2+c_3\Bigg(\Big(\Big(\Big(-k_1-2k_3\Big)k_4+k_1k_3\Big)k_2^2$$

$$+k_1k_3\left(k_3-k_4\right)k_2+2k_1k_3^2k_4\Big)P_{Total}c_3+\frac{k_2^2\left(\left(-4k_1-3k_3\right)k_4+k_1k_3\right)}{2}\Bigg)c_1$$

$$-k_4\left(\left(k_2^2P_{Total}-k_3^2P_{Total}\right)c_3+\frac{3k_2\left(k_2-\frac{k_3}{3}\right)}{2}\right)c_3^2k_3\Bigg)c_4$$

$$-\frac{c_1k_2k_1\left(\left(k_1+k_2\right)c_1+c_3\left(k_2+k_3\right)\right)\left(c_1k_1+c_3k_3\right)}{2}\Bigg)c_2^2+\Bigg(\Bigg(k_1k_2P_{Total}\Big(k_1$$

$$-k_4\Big)\left(k_1+k_4\right)c_1^3+\Bigg(2\Bigg(\Bigg(\left(-k_1-\frac{k_3}{2}\right)k_4^2-\frac{k_3k_4k_1}{2}+k_1^2k_3\Bigg)k_2$$

$$+\frac{k_1k_3k_4\left(k_1+k_4\right)}{2}\Bigg)P_{Total}c_3+\frac{k_1k_2k_4\left(k_1-3k_4\right)}{2}\Bigg)c_1^2+c_3\Bigg(\Big(\Big(\Big(-k_1$$

$$-2k_3\Big)k_4^2-k_3\left(k_1+k_3\right)k_4+k_1k_3^2\Big)k_2+k_3\left(k_1+k_3\right)k_4^2+2k_1k_3^2k_4\Big)P_{Total}c_3$$

$$-\frac{3k_4^2\left(\left(k_1+\frac{4k_3}{3}\right)k_2-\frac{k_1k_3}{3}\right)}{2}\Bigg)c_1-k_4\Bigg(P_{Total}\left(k_3+k_4\right)\left(k_2-k_3\right)c_3$$

$$+\left(\left(\frac{k_3}{2}+2k_4\right)k_2-\frac{k_3k_4}{2}\right)c_3^2k_3\Bigg)c_4$$

$$+\frac{\left(k_2\left(k_1-3k_4\right)c_1-3k_4c_3\left(k_2-\frac{k_3}{3}\right)\right)\left(c_1k_1+c_3k_3\right)^2}{2}\Bigg)c_4c_2$$

$$-\frac{k_4\left(\left(k_1+k_4\right)c_1+c_3\left(k_3+k_4\right)\right)\left(c_1k_1+c_3k_3+c_4k_4\right)c_3k_3c_4^2}{2}\Bigg)\Bigg)\Bigg/$$

$$\Bigg(\Bigg(c_1k_2k_1\left(\left(k_1+k_2\right)c_1+c_3\left(k_2+k_3\right)\right)c_2^2-\Bigg(k_2\left(k_1-3k_4\right)c_1-3k_4c_3\Bigg(k_2$$

$$-\frac{k_3}{3}\Bigg)\Bigg)\left(c_1k_1+c_3k_3\right)c_4c_2+k_4\left(\left(k_1+k_4\right)c_1+c_3\left(k_3+k_4\right)\right)c_3k_3c_4^2\Bigg)\Big(\Big(k_1$$

$$- k_4) k_2 c_1 - k_4 c_3 (k_2 - k_3)) (c_1 k_1 + c_3 k_3) c_4 c_2 \Big)$$

$$A_{Total} = 13.24004002$$

A. Case 3 - System 1 Random DSP (Tristability)

restart :

$k_1 := 100 : k_{b1} := 100 : k_{ub1} := 1 :$
$k_2 := 2 : k_{b2} := 1 : k_{ub2} := 1 :$
$k_3 := 0.01 : k_{b3} := 100 : k_{ub3} := 1 :$
$k_4 := 20 : k_{b4} := 0.1 : k_{ub4} := 1 :$

$P_{Total} := 10 :$

$$c_1 := \frac{k_{b1}}{k_1 + k_{ub1}} : c_2 := \frac{k_{b2}}{k_2 + k_{ub2}} : c_3 := \frac{k_{b3}}{k_3 + k_{ub3}} : c_4 := \frac{k_{b4}}{k_4 + k_{ub4}} :$$

Necessary conditions

Here we show that the necessary and sufficient condition for symmetry breaking (refer to analytical work) is satisfied for the parameters used.

1. $c_3 k_4 (k_2 - k_3) - c_1 k_2 (k_1 - k_4) > 0$

$$0 < 3782.178218 \qquad (2.2.1.1)$$

Symmetry Breaking

The invariant in the asymmetric steady states in System 1 Random ordered DSP case 3 post symmetry breaking is the sum of the concentrations of the partially modified substrates [A01] and [A10].
This is evaluated (based on the expression from the analytical work) for the kinetic parameters used in generating the plot.

$$A01 + A10 = \frac{-c_1 c_2 k_1 k_2 - c_3 c_4 k_3 k_4}{(((-c_1 - c_3) k_4 + c_1 k_1) k_2 + c_3 k_3 k_4) c_4 c_2}$$

$$A01 + A10 = 11.01047120 \qquad (2.2.2.1)$$

The intersection of the symmetric and asymmetric steady states on the bifurcation plot indicates

the presence of a pitch fork bifurcation.
The position of the pitch fork bifurcation is evaluated (based on the expression from the analytical work) for the kinetic parameters used in generating the plot.

$$
\begin{aligned}
A_{Total} = \Big(& 2\,(c_1 c_2 k_1 k_2 + c_3 c_4 k_3 k_4)\,\Big(-\frac{c_1 k_2^2 k_1\,((k_1+k_2)\,c_1 + c_3\,(k_2+k_3))\,c_2^3}{2} \\
& + \Big(\Big(k_1 k_2 P_{Total}\,(k_1-k_4)\,(k_1+k_2)\,c_1^3 + \Big((((-2k_1-k_3)\,k_4 + k_1\,(k_1+k_3))\,k_2^2 \\
& + (-k_1\,(k_1+k_3)\,k_4 + 2k_1^2 k_3)\,k_2 + k_1^2 k_3 k_4)\,P_{Total}\,c_3 \\
& + \frac{k_2\,((k_1-4k_4)\,k_2 - k_4 k_1)\,k_1}{2} \Big) c_1^2 + c_3 \Big((((-k_1-2k_3)\,k_4 + k_1 k_3)\,k_2^2 \\
& + k_1 k_3\,(k_3-k_4)\,k_2 + 2k_1 k_3^2 k_4)\,P_{Total}\,c_3 + \frac{k_2^2\,((-4k_1-3k_3)\,k_4 + k_1 k_3)}{2} \Big) c_1 \\
& - k_4 \Big((k_2^2 P_{Total} - k_3^2 P_{Total})\,c_3 + \frac{3k_2\left(k_2 - \frac{k_3}{3}\right)}{2} \Big) c_3^2 k_3 \Big) c_4 \\
& - \frac{c_1 k_2 k_1\,((k_1+k_2)\,c_1 + c_3\,(k_2+k_3))\,(c_1 k_1 + c_3 k_3)}{2} \Big) c_2^2 + \Big(\Big(k_1 k_2 P_{Total}\,(k_1 \\
& - k_4)\,(k_1+k_4)\,c_1^3 + \Big(2\Big(\Big(\Big(-k_1 - \frac{k_3}{2}\Big) k_4^2 - \frac{k_3 k_4 k_1}{2} + k_1^2 k_3\Big) k_2 \\
& + \frac{k_1 k_3 k_4\,(k_1+k_4)}{2} \Big) P_{Total}\,c_3 + \frac{k_1 k_2 k_4\,(k_1-3k_4)}{2} \Big) c_1^2 + c_3 \Big((((-k_1 \\
& - 2k_3)\,k_4^2 - k_3\,(k_1+k_3)\,k_4 + k_1 k_3^2)\,k_2 + k_3\,(k_1+k_3)\,k_4^2 + 2k_1 k_3^2 k_4)\,P_{Total}\,c_3 \\
& - \frac{3k_4^2\left(\left(k_1 + \frac{4k_3}{3}\right) k_2 - \frac{k_1 k_3}{3}\right)}{2} \Big) c_1 - k_4 \Big(P_{Total}\,(k_3+k_4)\,(k_2-k_3)\,c_3 \\
& + \left(\frac{k_3}{2} + 2k_4\right) k_2 - \frac{k_3 k_4}{2} \Big) c_3^2 k_3 \Big) c_4
\end{aligned}
$$

$$+\frac{\left(k_2\left(k_1-3\,k_4\right)c_1-3\,k_4\,c_3\left(k_2-\frac{k_3}{3}\right)\right)\left(c_1\,k_1+c_3\,k_3\right)^2}{2}\Bigg)c_4\,c_2$$

$$-\frac{k_4\left(\left(\left(k_1+k_4\right)c_1+c_3\left(k_3+k_4\right)\right)\left(c_1\,k_1+c_3\,k_3+c_4\,k_4\right)c_3\,k_3\,c_4^{\,2}\right.}{2}\Bigg)\Bigg)\Bigg/$$

$$\Bigg(\Bigg(c_1\,k_2\,k_1\left(\left(k_1+k_2\right)c_1+c_3\left(k_2+k_3\right)\right)c_2^{\,2}-\Bigg(k_2\left(k_1-3\,k_4\right)c_1-3\,k_4\,c_3\Bigg(k_2$$

$$-\frac{k_3}{3}\Bigg)\Bigg)\left(c_1\,k_1+c_3\,k_3\right)c_4\,c_2+k_4\left(\left(\left(k_1+k_4\right)c_1+c_3\left(k_3+k_4\right)\right)c_3\,k_3\,c_4^{\,2}\right)\Bigg)\Big(\Big(k_1$$

$$-k_4\Big)k_2\,c_1-k_4\,c_3\left(k_2-k_3\right)\Big)\left(c_1\,k_1+c_3\,k_3\right)c_4\,c_2\Bigg)$$

$$A_{Total}=28.25979662 \qquad \textbf{(2.2.2.2)}$$

B. Case 3 - System 3 Random DSP (Hopf)

restart :

$k_1 := 150 : k_{b1} := 100 : k_{ub1} := 1 :$
$k_2 := 50 : k_{b2} := 1 : k_{ub2} := 1 :$
$k_3 := 1 : k_{b3} := 0.01 : k_{ub3} := 1 :$
$k_4 := 10 : k_{b4} := 500 : k_{ub4} := 1 :$

$K1_{Total} := 1 : P1_{Total} := 1 :$
$K2_{Total} := 1 : P2_{Total} := 1 :$

B. Case 3 - System 3 Random DSP (PitchFork - Approximate robustness in A00 + A11)

restart :

$k_1 := 10 : k_{b1} := 1 : k_{ub1} := 1 :$
$k_2 := 1 : k_{b2} := 1 : k_{ub2} := 1 :$
$k_3 := 2 : k_{b3} := 1 : k_{ub3} := 1 :$
$k_4 := 5 : k_{b4} := 1 : k_{ub4} := 1 :$

$K1_{Total} := 1 : P1_{Total} := 1 :$

Appendix 2 Figure 1

B. Case 2 - Mixed-Random 3 DSP

restart :

$k_1 := 0.9 : k_{b1} := 1 : k_{ub1} := 1 :$
$k_2 := 0.8 : k_{b2} := 1 : k_{ub2} := 1 :$
$k_3 := 2 : k_{b3} := 1 : k_{ub3} := 1 :$
$k_4 := 1 :$

$K1_{Total} := 0.1 :$

$$c_1 := \frac{k_{b1}}{k_1 + k_{ub1}} : c_2 := \frac{k_{b2}}{k_2 + k_{ub2}} : c_3 := \frac{k_{b3}}{k_3 + k_{ub3}} :$$

Necessary Conditions

Here we show that the necessary and sufficient condition for symmetry breaking (refer to analytical work) is satisfied for the parameters used.

1. $k_2 < k_1$

$$0.8 < 0.9 \quad \textbf{(3.1.1.1)}$$

Symmetry Breaking (Invariants and position of symmetry in the bifurcation diagram along A_{Total})

The invariant in the asymmetric steady states in Mixed Random 3 ordered DSP case 2 post symmetry breaking is the concentrations of the partially modified substrates [A00] and [A11]. This is evaluated (based on the expression from the analytical work) for the kinetic parameters used in generating the plot.

$$A11 = \frac{K1_{Total}\, k_2}{2\, k_3}$$

$$A11 = 0.02000000000 \quad \textbf{(3.1.2.1)}$$

$$A00 = \frac{k_2}{(k_1 - k_2)\, c_1}$$

$$A00 = 15.20000000 \quad \textbf{(3.1.2.2)}$$

The intersection of the symmetric and asymmetric steady states on the bifurcation plot indicates the presence of a pitch fork bifurcation.

The position of the pitch fork bifurcation is evaluated (based on the expression from the analytical work) for the kinetic parameters used in generating the plot.

$$A_{Total} = \frac{1}{2\left(k_1 - k_2\right) c_1 c_2 k_3 k_1} \left(\left(K1_{Total} \left(k_2 + 2 k_3\right) c_2 + 4 k_3 \right) c_1 k_1^2 - \left(K1_{Total} k_2 c_1 - 2 k_3 \right) c_2 k_2 k_1 - 2 K1_{Total} k_2^2 c_1 c_2 k_3 \right)$$

=

$$A_{Total} = 47.80888889 \qquad \textbf{(3.1.2.3)}$$

Appendix 2 Figure 2

A. Case 3 - System 1 Random DSP (Hopf)

restart :

$k_1 := 100 : k_{b1} := 100 : k_{ub1} := 1 :$
$k_2 := 20 : k_{b2} := 1 : k_{ub2} := 1 :$
$k_3 := 1 : k_{b3} := 100 : k_{ub3} := 1 :$
$k_4 := 2 : k_{b4} := 1 : k_{ub4} := 1 :$

$P_{Total} := 1 :$

A. Case 3 - System 1 Random DSP (Oscillations - Dynamic response)

restart :

$A_{Total} := 12.95 :$

A. Case 3 - System 1 Random DSP (Pitchfork)

restart :

$k_1 := 0.1 : k_{b1} := 1 : k_{ub1} := 1 :$
$k_2 := 1 : k_{b2} := 1 : k_{ub2} := 1 :$
$k_3 := 2 : k_{b3} := 1 : k_{ub3} := 1 :$
$k_4 := 5 : k_{b4} := 1 : k_{ub4} := 1 :$

$P_{Total} := 1 :$

$$c_1 := \frac{k_{b1}}{k_1 + k_{ub1}} : c_2 := \frac{k_{b2}}{k_2 + k_{ub2}} : c_3 := \frac{k_{b3}}{k_3 + k_{ub3}} : c_4 := \frac{k_{b4}}{k_4 + k_{ub4}} :$$

Necessary conditions

Here we show that the necessary and sufficient condition for symmetry breaking (refer to analytical work) is satisfied for the parameters used.

1. $c_3 k_4 (k_2 - k_3) - c_1 k_2 (k_1 - k_4) > 0$

$$0 < 2.787878788 \tag{4.3.1.1}$$

Symmetry Breaking (Invariants and position of symmetry in the bifurcation diagram along A_{Total})

The invariant in the asymmetric steady states in System Random 1 ordered distributive DSP post case 3 symmetry breaking is the sum of the concentrations of the partially modified substrates [A00] and [A11].
This is evaluated (based on the expression from the analytical work) for the kinetic parameters used in generating the plot.

$$A01 + A10 = \frac{-c_1 c_2 k_1 k_2 - c_3 c_4 k_3 k_4}{(((-c_1 - c_3) k_4 + c_1 k_1) k_2 + c_3 k_3 k_4) c_4 c_2}$$

$$A01 + A10 = 2.586956524 \tag{4.3.2.1}$$

The intersection of the symmetric and asymmetric steady states on the bifurcation plot indicates the presence of a pitch fork bifurcation.
The position of the pitch fork bifurcation is evaluated (based on the expression from the analytical work) for the kinetic parameters used in generating the plot.

$$A_{Total} = \Bigg(2 (c_1 c_2 k_1 k_2 + c_3 c_4 k_3 k_4) \Bigg(-\frac{c_1 k_2^2 k_1 ((k_1 + k_2) c_1 + c_3 (k_2 + k_3)) c_2^3}{2}$$
$$+ \Bigg(\Bigg(k_1 k_2 P_{Total} (k_1 - k_4) (k_1 + k_2) c_1^3 + \Big((((-2 k_1 - k_3) k_4 + k_1 (k_1 + k_3)) k_2^2$$
$$+ (-k_1 (k_1 + k_3) k_4 + 2 k_1^2 k_3) k_2 + k_1^2 k_3 k_4) P_{Total} c_3$$
$$+ \frac{k_2 ((k_1 - 4 k_4) k_2 - k_4 k_1) k_1}{2}\Big) c_1^2 + c_3 \Big((((-k_1 - 2 k_3) k_4 + k_1 k_3) k_2^2$$
$$+ k_1 k_3 (k_3 - k_4) k_2 + 2 k_1 k_3^2 k_4) P_{Total} c_3 + \frac{k_2^2 ((-4 k_1 - 3 k_3) k_4 + k_1 k_3)}{2}\Big) c_1$$

$$- k_4 \left(\left(k_2^2 P_{Total} - k_3^2 P_{Total} \right) c_3 + \frac{3 k_2 \left(k_2 - \frac{k_3}{3} \right)}{2} \right) c_3^2 k_3 \Bigg) c_4$$

$$- \frac{c_1 k_2 k_1 \left(\left(k_1 + k_2 \right) c_1 + c_3 \left(k_2 + k_3 \right) \right) \left(c_1 k_1 + c_3 k_3 \right)}{2} \Bigg) c_2^2 + \Bigg(\Bigg(k_1 k_2 P_{Total} \left(k_1 \right.$$

$$\left. - k_4 \right) \left(k_1 + k_4 \right) c_1^3 + \left(2 \left(\left(\left(-k_1 - \frac{k_3}{2} \right) k_4^2 - \frac{k_3 k_4 k_1}{2} + k_1^2 k_3 \right) k_2 \right. \right.$$

$$\left. \left. + \frac{k_1 k_3 k_4 \left(k_1 + k_4 \right)}{2} \right) P_{Total} c_3 + \frac{k_1 k_2 k_4 \left(k_1 - 3 k_4 \right)}{2} \right) c_1^2 + c_3 \Bigg(\Big(\Big(\Big(-k_1$$

$$- 2 k_3 \Big) k_4^2 - k_3 \left(k_1 + k_3 \right) k_4 + k_1 k_3^2 \Big) k_2 + k_3 \left(k_1 + k_3 \right) k_4^2 + 2 k_1 k_3^2 k_4 \Big) P_{Total} c_3$$

$$- \frac{3 k_4^2 \left(\left(k_1 + \frac{4 k_3}{3} \right) k_2 - \frac{k_1 k_3}{3} \right)}{2} \Bigg) c_1 - k_4 \Bigg(P_{Total} \left(k_3 + k_4 \right) \left(k_2 - k_3 \right) c_3$$

$$+ \left(\left(\frac{k_3}{2} + 2 k_4 \right) k_2 - \frac{k_3 k_4}{2} \right) c_3^2 k_3 \Bigg) c_4$$

$$+ \frac{\left(k_2 \left(k_1 - 3 k_4 \right) c_1 - 3 k_4 c_3 \left(k_2 - \frac{k_3}{3} \right) \right) \left(c_1 k_1 + c_3 k_3 \right)^2}{2} \Bigg) c_4 c_2$$

$$- \frac{k_4 \left(\left(k_1 + k_4 \right) c_1 + c_3 \left(k_3 + k_4 \right) \right) \left(c_1 k_1 + c_3 k_3 + c_4 k_4 \right) c_3 k_3 c_4^2}{2} \Bigg) \Bigg) \Bigg/$$

$$\Bigg(\Bigg(c_1 k_2 k_1 \left(\left(k_1 + k_2 \right) c_1 + c_3 \left(k_2 + k_3 \right) \right) c_2^2 - \Bigg(k_2 \left(k_1 - 3 k_4 \right) c_1 - 3 k_4 c_3 \Bigg(k_2$$

$$- \frac{k_3}{3} \Bigg) \Bigg) \left(c_1 k_1 + c_3 k_3 \right) c_4 c_2 + k_4 \left(\left(k_1 + k_4 \right) c_1 + c_3 \left(k_3 + k_4 \right) \right) c_3 k_3 c_4^2 \Bigg) \Big(\Big(k_1$$

$$- k_4 \Big) k_2 c_1 - k_4 c_3 \left(k_2 - k_3 \right) \Big) \left(c_1 k_1 + c_3 k_3 \right) c_4 c_2 \Bigg)$$

$$A_{Total} = 8.713626644 \qquad \textbf{(4.3.2.2)}$$

Appendix 2 Figure 3

Case 1 - System 1 Random DSP (Independent leg bifurcation)

$restart:$

$k_1 := 1 : k_{b1} := 1 : k_{ub1} := 1 :$
$k_2 := 1 : k_{b2} := 1 : k_{ub2} := 1 :$
$P_{Total} := 1 :$

$$c_1 := \frac{k_{b1}}{k_1 + k_{ub1}} : c_2 := \frac{k_{b2}}{k_2 + k_{ub2}} :$$

$a_1 := 0.1 : a_{b1} := 1 : a_{ub1} := 1 :$
$a_2 := 1 : a_{b2} := 1 : a_{ub2} := 1 :$

$$d_1 := \frac{a_{b1}}{a_1 + a_{ub1}} : d_2 := \frac{a_{b2}}{a_2 + a_{ub2}} :$$

Necessary Constraint

Here we show that the necessary and sufficient conditions for symmetry breaking (refer to analytical work) is satisfied for the parameters used.

1. $c_1 a_2 (k_2 - k_1) + a_1 k_2 (a_2 - a_1) > 0$

$$0 < 0.6000000000 \quad \textbf{(5.1.1.1)}$$

Symmetry Breaking (Invariants and position of symmetry in the bifurcation diagram along A_{Total})

The invariant in the asymmetric steady states in System Random 1 ordered distributive DSP post case 1 symmetry breaking is the concentrations of the partially modified substrates [A01] and [A10].
This is evaluated (based on the expression from the analytical work) for the kinetic parameters used in generating the plot.

$$A10 := -\frac{d_1 k_2 a_1}{d_2 \left(\left(d_1 \left(a_1 - a_2 \right) - c_1 a_2 \right) k_2 + c_1 k_1 a_2 \right)}$$

$$A10 := 0.2222222222 \quad \textbf{(5.1.2.1)}$$

$$A01 := -\frac{c_1 k_1 a_2}{c_2 \left(\left(\left(k_1 - k_2 \right) c_1 - d_1 k_2 \right) a_2 + d_1 k_2 a_1 \right)}$$

$$A01 := 1.222222222 \quad \textbf{(5.1.2.2)}$$

The intersection of the symmetric and asymmetric steady states on the bifurcation plot indicates the presence of a pitch fork bifurcation.

The position of the pitch fork bifurcation is evaluated (based on the expression from the analytical work) for the kinetic parameters used in generating the plot.

$$A_{Total} = \Big(d_2 \left(-c_2 \left(c_1 + d_1\right) \left(c_1 P_{Total} + d_1 P_{Total} + 2\right) k_2^{\,2} - c_1 k_1 \left(c_1 + d_1\right) k_2 + c_1^{\,2} c_2 k_1^{\,2} P_{Total}\right) a_2^{\,2} + 2\, d_1 \left(\left(-\frac{c_1}{2} - \frac{d_1}{2}\right) k_2 + c_1 d_2 k_1 P_{Total}\right) c_2 k_2 a_1 a_2 + a_1^{\,2} c_2 d_1^{\,2} d_2 k_2^{\,2} P_{Total}\Big) \Big/ \left(\left(\left(\left(-c_1 - d_1\right) k_2 + c_1 k_1\right) a_2 + a_1 d_1 k_2\right) a_2 \left(c_1 + d_1\right) c_2 d_2 k_2\right)$$

$$A_{Total} = 5.308243724 \qquad \textbf{(5.1.2.3)}$$

Appendix 2 Figure 4

Case 3 - System 3 Random DSP (PitchFork - Approximate robustness in A01 + A10)

restart :

$k_1 := 1 : k_{b1} := 10 : k_{ub1} := 1 :$
$k_2 := 2 : k_{b2} := 1 : k_{ub2} := 1 :$
$k_3 := 1 : k_{b3} := 10 : k_{ub3} := 1 :$
$k_4 := 20 : k_{b4} := 1 : k_{ub4} := 1 :$

$K1_{Total} := 5 : P1_{Total} := 5 :$
$K2_{Total} := 5 : P2_{Total} := 5 :$

Appendix 2 Figure 5

Case 2 - System 3 Random DSP (Approximate robustness in non exact symmetric state)

restart :

$k_1 := 1.25 : k_{b1} := 1 : k_{ub1} := 1 :$
$k_2 := 1.1 : k_{b2} := 1 : k_{ub2} := 1 :$
$k_3 := 2.5 : k_{b3} := 1 : k_{ub3} := 1 :$
$k_4 := 0.4 : k_{b4} := 1 : k_{ub4} := 1 :$

$a_1 := 1.25 : a_{b1} := 1 : a_{ub1} := 1 :$
$a_{b2} := 1 : a_{ub2} := 1 :$

$a_3 := 2.5 : a_{b3} := 1 : a_{ub3} := 1 :$
$a_4 := 0.4 : a_{b4} := 1 : a_{ub4} := 1 :$

$K1_{Total} := 1 : P1_{Total} := 1 :$

Exact symmetry used for comparison

$k_1 := 1.25 : k_{b1} := 1 : k_{ub1} := 1 :$
$k_2 := 1.1 : k_{b2} := 1 : k_{ub2} := 1 :$
$k_3 := 2.5 : k_{b3} := 1 : k_{ub3} := 1 :$
$k_4 := 0.4 : k_{b4} := 1 : k_{ub4} := 1 :$

$K1_{Total} := 1 : P1_{Total} := 1 :$

Appendix 2 Figure 6

Approximate concentration robustness in near symmetric systems (Ordered DSP with common kinase common phosphatase)

restart :

$k_{b1} := 1 : k_{ub1} := 1 :$
$k_2 := 0.5 : k_{b2} := 1 : k_{ub2} := 1 :$
$k_3 := 0.1 : k_{b3} := 1 : k_{ub3} := 1 :$
$k_4 := 0.5 : k_{b2} := 1 : k_{ub2} := 1 :$

$P_{Total} := 0.1 : K_{Total} := 0.1 :$

k1 is varied between 50% to 150% from it's symmetric value (0.1). All other parameters remain the same as those used in Fig2A.

Appendix 2 Figure 7

A. Case 3 - System 1 Random DSP

restart :

$k_1 := 30 : k_{b1} := 100 : k_{ub1} := 1 :$
$k_2 := 2 : k_{b2} := 1 : k_{ub2} := 1 :$
$k_3 := 0.3 : k_{b3} := 100 : k_{ub3} := 1 :$

$k_4 := 20 : k_{b4} := 0.1 : k_{ub4} := 1 :$

$K1_{Total} := 1 : P1_{Total} := 1 :$

B. Case 3 - System 1 Random DSP

Same kinetic parameters as above

$K1_{Total} := 20 : P1_{Total} := 20 :$

Appendix 2 Figure 8

Case 1 - Triple site phosphorylation

restart :

$k_1 := 0.1 : k_{b1} := 1 : k_{ub1} := 1 :$
$k_2 := 1.5 : k_{b2} := 1 : k_{ub2} := 1 :$
$k_3 := 2 : k_{b3} := 1 : k_{ub3} := 1 :$

$P_{Total} := 0.1 :$

$$c_1 := \frac{k_{b1}}{k_1 + k_{ub1}} : c_2 := \frac{k_{b2}}{k_2 + k_{ub2}} : c_3 := \frac{k_{b3}}{k_3 + k_{ub3}} :$$

Necessary Constraint

Here we show that the necessary and sufficient condition for symmetry breaking (refer to analytical work) is satisfied for the parameters used.

1. $k_3 > k_1$

$$0.1 < 2. \qquad \textbf{(10.1.1.1)}$$

Symmetry Breaking (Invariants and position of symmetry in the bifurcation diagram along A_{Total})

The invariant in the asymmetric steady states in distributive ordered TSP post case 1 symmetry breaking is the sum of the concentrations of the partially modified substrates [Ap] and [App].

This is evaluated (based on the expression from the analytical work) for the kinetic parameters used in generating the plot.

$$Ap + App = -\frac{k_1}{c_3 (k_1 - k_3)}$$

$$Ap + App = 0.1578947368 \tag{10.1.2.1}$$

The intersection of the symmetric and asymmetric steady states on the bifurcation plot indicates the presence of a pitch fork bifurcation.
The position of the pitch fork bifurcation is evaluated (based on the expression from the analytical work) for the kinetic parameters used in generating the plot.

$$A_{Total} = \frac{\left(2 c_1 c_2 c_3 k_1^2 - 2 c_1 c_2 c_3 k_1 k_3 + 2 c_1 c_3^2 k_1^2 - 2 c_1 c_3^2 k_3^2\right) P_{Total}}{(k_1 - k_3) c_1 c_3 (c_2 k_1 - c_3 k_1 + 3 c_3 k_3)} + \frac{-c_1 c_2 k_1^2 + c_1 c_3 k_1^2 - 3 c_1 c_3 k_1 k_3 - c_2 c_3 k_1 k_3 + c_3^2 k_1 k_3 - 3 c_3^2 k_3^2}{(k_1 - k_3) c_1 c_3 (c_2 k_1 - c_3 k_1 + 3 c_3 k_3)}$$

$$=$$

$$A_{Total} = 1.389543626 \tag{10.1.2.2}$$

Appendix 2 Figure 9

Ordered DSP (Common kinase common phosphatase) - ACR with non-symmetric kinetics along changing A_{Total}

$restart:$

$k_1 := 1 : k_{b1} := 1 : k_{ub1} := 1 :$
$k_2 := 2 : k_{b2} := 1 : k_{ub2} := 1 :$
$k_3 := 0.5 : k_{b3} := 1 : k_{ub3} := 1 :$
$k_4 := 1.2 : k_{b4} := 11 : k_{ub4} := 1 :$

$K_{Total} := 1 : P_{Total} := 1 :$

$$c_1 := \frac{k_{b1}}{k_1 + k_{ub1}} : c_2 := \frac{k_{b2}}{k_2 + k_{ub2}} : c_3 := \frac{k_{b3}}{k_3 + k_{ub3}} : c_4 := \frac{k_{b4}}{k_4 + k_{ub4}} :$$

Necessary and sufficient condition

Here we show that the necssary and sufficient condition for ACR in Ap is satisfied by the

parameters used.

$$1. \quad \frac{k_3 P_{Total}}{c_2 \left(k_2 K_{Total} - k_3 P_{Total}\right)} = - \frac{k_1 K_{Total}}{c_4 \left(K_{Total} k_1 - k_4 P_{Total}\right)}$$

$$1.000000000 = 1.000000000 \qquad \textbf{(11.1.1.1)}$$

ACR concentration of Ap

The ACR concentration of Ap (as seen in the analytical work in section 5.1) is thus given by,

$$Ap = \frac{k_3 P_{Total}}{c_2 \left(k_2 K_{Total} - k_3 P_{Total}\right)}$$

$$Ap = 1.000000000 \qquad \textbf{(11.1.2.1)}$$

This is verified in the compuatational result in the figure.

Double Site Ordered Distributive Phosphorylation : Common Kinase Common Phosphatase
Case 1 Symmetry - Present and Breaks

In this Maple file we analytically show the presence of symmetry breaking in ordered distributive DSP network with common kinase and common phosphatase effecting phosphorylation and dephoshorylation respectively. We do this by first describing the model as a system of ODEs and imposing the kinetic constraints pertaining to case 1 symmetry. We further describe the enzyme and substrate conservations associated with the model. By solving for the steady states of the system of ODEs we obtain relations between substrate variables (concentrations) in terms of each other and the free enzyme concentrations. In ordered distributive DSP (with common kinase common phosphatase), $K_{Total} = P_{Total}$ is required for exact case 1 symmetry to be present. We use this information to get a further simplified expression describing all possible steady states of the system. With case 1 symmetry in the ordered distributive MSP, the free enzyme kinase and phosphatase share a strict symmetry in the symmetric steady state ([K] = [P]). Thus by isolating steady states not of this type from the earlier correlation, we ascertain the features of the asymmetric states emerging from symmetry breaking. This procedure is carried out in detail below using built in Maple commands.

We initialize the Maple file with the *restart* command and load the relevant libraries of inbuilt Maple functions (*LinearAlgebra, VectorCalculus, Student[LinearAlgebra]*)

$restart : with(LinearAlgebra) : with(VectorCalculus) : with(Student[LinearAlgebra]) :$

The system is modelled as a set of ODEs using the kinetic nomenclature described in the main text and supplementary figure (refer to Appendix 2 figure 10). Here dA represents d[A]/dt and similarly in the case of the other variables. At steady state thus, the right hand sides of each of these expressions will be equal to zero.

$$dA := k_4 \cdot ApP + k_{ub1} \cdot AK - k_{b1} \cdot A \cdot K :$$
$$dAp := k_1 \cdot AK + k_3 \cdot AppP + k_{ub2} \cdot ApK + k_{ub4} \cdot ApP - k_{b2} \cdot Ap \cdot K - k_{b4} \cdot Ap \cdot P :$$
$$dApp := k_2 \cdot ApK + k_{ub3} \cdot AppP - k_{b3} \cdot App \cdot P :$$

$$dAK := k_{b1} \cdot A \cdot K - (k_{ub1} + k_1) \cdot AK :$$
$$dApK := k_{b2} \cdot Ap \cdot K - (k_{ub2} + k_2) \cdot ApK :$$
$$dAppP := k_{b3} \cdot App \cdot P - (k_{ub3} + k_3) \cdot AppP :$$
$$dApP := k_{b4} \cdot Ap \cdot P - (k_{ub4} + k_4) \cdot ApP :$$

$$dK := -k_{b1} \cdot A \cdot K + (k_{ub1} + k_1) \cdot AK - k_{b2} \cdot Ap \cdot K + (k_{ub2} + k_2) \cdot ApK :$$
$$dP := -k_{b3} \cdot App \cdot P + (k_{ub3} + k_3) \cdot AppP - k_{b4} \cdot Ap \cdot P + (k_{ub4} + k_4) \cdot ApP :$$

The model is also associated with conservation conditions which are described below. Here we store the conservation expressions as ACon, PCon and KCon for the substrate and the respective enzymes. Each of these expressions is always equal to zero (both in the transient behavior and at steady state).

$ACon := A_{Total} - A - Ap - App - AK - ApK - AppP - ApP:$
$PCon := P_{Total} - P - AppP - ApP:$
$KCon := K_{Total} - K - AK - ApK:$

Kinetic constraints for case 1 symmetry (refer main text) are imposed on the original model.

$k_3 := k_1 : k_{ub3} := k_{ub1} : k_{b3} := k_{b1} :$
$k_4 := k_2 : k_{ub4} := k_{ub2} : k_{b4} := k_{b2} :$

In addition to the kinetic constraints the total enzyme concentrations of kinase and phosphatase also need to be equal for exact case 1 symmetry to be present. This is imposed as shown below.

$K_{Total} := P_{Total} :$

At this stage we introduce auxiliary constants c_1 and c_2 in place of the binding constants so as to make further analytical expressions more accessible.

$k_{b1} := c_1 \cdot (k_1 + k_{ub1}) :$
$k_{b2} := c_2 \cdot (k_2 + k_{ub2}) :$

Once this is done, we solve for the steady state of the system in terms of fewer key variables. In this context we want to solve all variables in terms of the concentrations of the free enzymes ([K] & [P]) and the concentration of the partially modified substrate ([Ap]). In order to do this, we use the Maple command *solve,* which solves the supplied equation for a given variable. We first solve for the individual complexes using their corresponding differential equation. An example of this (using [AK]) is given below in detail.

The differential equation of [AK] is given by,

$\frac{d\,[AK]}{dt} = dAK$

$$\frac{d\,[AK]}{dt} = c_1 \left(k_{ub1} + k_1\right) A\,K - \left(k_{ub1} + k_1\right) AK \quad \textbf{(1)}$$

The *solve* command by Maple, solves this equation for the given variable (in this case [AK]). We in turn store this value (the solution returned by the *solve* command) in [AK]. This is performed by the following command.

$AK := solve(dAK, AK)$

$$AK := K\,A\,c_1 \quad \textbf{(2)}$$

This operation is repeated for the other complexes and substrate forms as well.

$ApK := solve(dApK, ApK) :$

$AppP := solve(dAppP, AppP)$:
$ApP := solve(dApP, ApP)$:

$A := solve(dA, A)$:
$App := solve(dApp, App)$:

Doing this results in the following correlations between the concentrations of the various substrate forms at steady state.

$$App = \frac{K\,Ap\,c_2\,k_2}{c_1\,P\,k_1}$$
$$A = \frac{P\,Ap\,c_2\,k_2}{c_1\,K\,k_1}$$

Proof for invariant in the asymmetric branches

We know that PCon and KCon are both individually always equal to zero. Thus at a given steady state, PCon - KCon must also be equal to zero.

We thus introduce the term T = (KCon - PCon) = 0 and also introduce a new ratio, ϵ = [K]/[P]. Note: As discussed in the main text, the symmetric steady state is one where [K] = [P] or ϵ = 1. Since we are isolating solutions of asymmetry, we are primarily interested in solutions that permit, $\epsilon \neq 1$.
$T := KCon - PCon = 0$:
$K := \epsilon \cdot P$:

The following Maple command (*simplify*), simplifies the expression algebraically.

$$simplify(T) = -\frac{P\left(Ap\left(k_1 - k_2\right)c_2 + k_1\right)(\epsilon - 1)}{k_1} = 0$$

From this we can ascertain that, should an asymmetric steady state exist (where $\epsilon \neq 1$) - the term ($Ap\left(k_1 - k_2\right)c_2 + k_1$) in the expression needs to be zero. We note that this term is a expression in the partial substrate form [Ap] and kinetic constants. Thus solving this to isolate the partial substrate form we get the following. Here we use the *solve* command from Maple to solve T for [Ap] as shown below.

$Ap = solve(T, Ap)$

$$Ap = -\frac{k_1}{\left(k_1 - k_2\right)c_2} \quad \textbf{(1.1)}$$

Thus we can see that in an asymmetric steady state, the value of the partially modified substrate is fixed and is given by the above expression involving only a few key kinetic

parameters.

Necessary conditions for symmetry breaking

Since substrate concentrations are always positive, the expression in equation 1.1 should be positive. As the involved terms (kinetic constants) are by definition positive, we get the necessary condition for asymmetric states to exist as follows.

1. $k_2 > k_1$

Sufficiency of necessary conditions

In this section of the proof we show the necessary conditions shown above are also sufficient for an asymmetric steady state to exist for some positive A_{Total} value. i.e. We show that upon a bifurcation along A_{Total} we are bound to encounter symmetry breaking provided the necessary conditions are satisfied. Note that a feasible steady state in this context is one in which the concentrations of all substrates, complexes and enzymes are positive.

We do this by showing that the asymmetric states defined by the invariant concentration of [Ap] described above is indeed a feasible solution for the system of ODEs at some positive A_{Total} value.

In an asymmetric steady state, as seen above the concentration of [Ap] is fixed by a few kinetic constants,

$$Ap := -\frac{k_1}{(k_1 - k_2)\, c_2} = -\frac{k_1}{(k_1 - k_2)\, c_2}$$

The other variables in this asymmetric state are thus given by (we obtain this by using the correlation obtained earlier between the different concentrations)

$$A = -\frac{k_2}{(k_1 - k_2)\, c_1\, \epsilon}$$

$$App = -\frac{\epsilon\, k_2}{(k_1 - k_2)\, c_1}$$

$$AK = -\frac{P\, k_2}{k_1 - k_2}$$

$$ApK = -\frac{\epsilon\, P\, k_1}{k_1 - k_2}$$

$$AppP = -\frac{P\, \epsilon\, k_2}{k_1 - k_2}$$

$$ApP = -\frac{P\,k_1}{k_1 - k_2}$$

The system of ODE is also satisfied at this point, as is verified below.

$simplify(dA) = 0$
$simplify(dAp) = 0$
$simplify(dApp) = 0$
$simplify(dAK) = 0$
$simplify(dApK) = 0$
$simplify(dAppP) = 0$
$simplify(dApP) = 0$
$simplify(dK) = 0$
$simplify(dP) = 0$

Hence all that remains to be shown is that the variables (as described above) are positive for some value of A_{Total}.
This is true if and only if

1. **Necessary condition $(k_2 > k_1)$ is satisfied**
2. **P and ϵ are positive.**

Now by using the total conservation of phosphatase in the system (PCon = 0), we obtain an algebraic expression for [P] in terms of P_{Total}, kinetic constants and ϵ as shown below. The Maple command *solve* is used for this purpose.

$$P := solve(PCon, P) = -\frac{P_{Total}\,(k_1 - k_2)}{k_2\,(\epsilon + 1)}$$

Hence if ϵ is positive P is automatically positive (provided necessary conditions are satisfied).

Thus this means that for every value of $\epsilon \neq 1$, all concentrations are positive and the conservation of kinase and phosphatase is also satisfied.

Since the concentrations are all positive there exists a unique finite A_{Total} value for the given ϵ (permitting asymmetric states).

Hence we have proved that symmetry breaking is guaranteed for some finite positive A_{Total}, provided the necessary conditions above are satisfied - making those conditions sufficient for the behavior.

Prediction of position of pitchfork bifurcation along A_{Total}

Here we predict the value of A_{Total} at which symmetry breaking occurs via a pitchfork bifurcation. This point in the bifurcation is characterized by the intersection of both the symmetric steady state branch and the asymmetric steady state branches.

Hence at the position of symmetry breaking, we know two facts.

1. The system is still symmetric, hence [A] = [App] and [K] = [P].
2. The invariant describing the asymmetric steady state is also true.

Using these two information, we can simplify the original system considerably as follows.

$K := P : \epsilon := 1 :$

$$Ap := -\frac{k_1}{(k_1 - k_2)\, c_2} :$$

Now, by solving the conservation expression for the substrate we can isolate the value of A_{Total} when the asymmetric steady states and the symmetric steady state intersect (indicating the pitchfork bifurcation point)

$A_{Total} = simplify(solve(simplify(ACon), A_{Total}))$

$$A_{Total} = \frac{(-c_1\, c_2\, P_{Total} - 2\, c_2)\, k_2^2 - k_1\, c_1\, k_2 + P_{Total}\, k_1^2\, c_1\, c_2}{(k_1 - k_2)\, c_1\, c_2\, k_2} \qquad \textbf{(4.1)}$$

A cross verification of this analytical work is carried out in the read me file for the parameters used in generating the figures (Fig 2A).

Double Site Ordered Distributive Phosphorylation : Separate Kinase Separate Phosphatase
Case 1 Symmetry - Present and Cannot Break

In this Maple file we analytically show the infeasibility of symmetry breaking in ordered distributive DSP network with a unique kinase and unique phosphatase effecting phosphorylation and dephoshorylation on each modification site. We do this by first describing the model as a system of ODEs and imposing the kinetic constraints pertaining to case 1 symmetry. We further describe the enzyme and substrate conservations associated with the model. By solving for the steady state of the system of ODEs we obtain relations between substrate variables (concentrations) in terms of each other and the free enzyme concentrations. In ordered distributive DSP (with separate kinase separate phosphatase), $K1_{Total} = P2_{Total}$ and $K2_{Total} = P1_{Total}$ is required for exact case 1 symmetry to be present. We use this information to get a further simplified expression describing all possible steady states of the system. By subsequently analyzing the steady state of the system using the conservation conditions, we show that a steady state violating case 1 symmetry between [A] and [App] is not possible in this network for any choice of kinetics or total enzyme concentrations, thus ruling out symmetry breaking.

We initialize the maple file with the *restart* command and load the relevant libraries of inbuilt Maple functions (*LinearAlgebra, VectorCalculus, Student[LinearAlgebra]*)

$restart : with(LinearAlgebra) : with(VectorCalculus) : with(Student[LinearAlgebra]) :$

The system is modelled as a set of ODEs using the kinetic nomenclature described in the main text and supplementary figure (refer to Appendix 2 figure 10). Here dA represents d[A]/dt and similarly in the case of the other variables. At steady state thus, each of the right hand sides of these expressions will be equal to zero.

$$dA := k_4 \cdot ApP1 + k_{ub1} \cdot AK1 - k_{b1} \cdot A \cdot K1 :$$
$$dAp := k_1 \cdot AK1 + k_3 \cdot AppP2 + k_{ub2} \cdot ApK2 + k_{ub4} \cdot ApP1 - k_{b2} \cdot Ap \cdot K2 - k_{b4} \cdot Ap \cdot P1 :$$
$$dApp := k_2 \cdot ApK2 + k_{ub3} \cdot AppP2 - k_{b3} \cdot App \cdot P2 :$$

$$dAK1 := k_{b1} \cdot A \cdot K1 - (k_{ub1} + k_1) \cdot AK1 :$$
$$dApK2 := k_{b2} \cdot Ap \cdot K2 - (k_{ub2} + k_2) \cdot ApK2 :$$
$$dAppP2 := k_{b3} \cdot App \cdot P2 - (k_{ub3} + k_3) \cdot AppP2 :$$
$$dApP1 := k_{b4} \cdot Ap \cdot P1 - (k_{ub4} + k_4) \cdot ApP1 :$$

$$dK1 := -k_{b1} \cdot A \cdot K1 + (k_{ub1} + k_1) \cdot AK1 :$$
$$dK2 := -k_{b2} \cdot Ap \cdot K2 + (k_{ub2} + k_2) \cdot ApK2 :$$
$$dP2 := -k_{b3} \cdot App \cdot P2 + (k_{ub3} + k_3) \cdot AppP2 :$$
$$dP1 := -k_{b4} \cdot Ap \cdot P1 + (k_{ub4} + k_4) \cdot ApP1 :$$

The model is also associated with conservation conditions which are described below. Here we store the conservation expressions as ACon, P1Con, P2Con, K1Con and K2Con for the substrate

and the respective enzymes. Each of these expressions is always equal to zero (both in the transient behavior and at steady state).

$ACon := A_{Total} - A - Ap - App - AK1 - ApK2 - AppP2 - ApP1 :$
$P1Con := P1_{Total} - P1 - ApP1 :$
$K1Con := K1_{Total} - K1 - AK1 :$
$P2Con := P2_{Total} - P2 - AppP2 :$
$K2Con := K2_{Total} - K2 - ApK2 :$

Kinetic constraints for case 1 symmetry (refer main text) are imposed on the original model.

$k_3 := k_1 : k_{ub3} := k_{ub1} : k_{b3} := k_{b1} :$
$k_4 := k_2 : k_{ub4} := k_{ub2} : k_{b4} := k_{b2} :$

In addition to the kinetic constraints the total enzyme concentrations of corresponding kinases and phosphatases also need to be equal for exact case 1 symmetry to be present. This is imposed as shown below.

$K1_{Total} := P2_{Total} :$
$K2_{Total} := P1_{Total} :$

At this stage we introduce auxiliary constants c_1 and c_2 in place of the binding constants so as to make further analytical expressions more accessible.

$k_{b1} := c_1 \cdot (k_1 + k_{ub1}) :$
$k_{b2} := c_2 \cdot (k_2 + k_{ub2}) :$

Once this is done, we solve for the steady state of the system in terms of fewer key variables. In this context we want to solve all variables in terms of the concentrations of the free enzymes ([K1], [K2], [P1] and [P2]) and concentration of the partially modified substrate ([Ap]). In order to do this, we use the Maple command *solve,* which solves the equation supplied for a given variable. We first solve for the individual complexes using their corresponding differential equation. An example of this (using [AK1]) is given below in detail.
The differential equation of [AK1] is given by,

$$\frac{d\,[AK1]}{dt} = dAK1 \qquad \frac{d\,[AK1]}{dt} = c_1\,(k_{ub1} + k_1)\,A\,K1 - (k_{ub1} + k_1)\,AK1$$

The *solve* command by Maple, solves this equation for the given variable (in this case [AK1]). We in turn store this value (the solution returned by the *solve* command) in [AK1]. This is performed by the following command.

$AK1 := solve(dAK1, AK1)$

$$AK1 := K1\,A\,c_1 \qquad \textbf{(1)}$$

This operation is repeated for the other complexes and substrate forms as well.

$ApK2 := solve(dApK2, ApK2)$:
$AppP2 := solve(dAppP2, AppP2)$:
$ApP1 := solve(dApP1, ApP1)$:

$A := solve(dA, A)$:
$App := solve(dApp, App)$:

Doing this results in the following correlations between the concentrations of the various substrate forms at steady state.

$$A = \frac{P1\ Ap\ c_2\ k_2}{c_1\ K1\ k_1}$$

$$App = \frac{K2\ Ap\ c_2\ k_2}{P2\ c_1\ k_1}$$

Proof for infeasibility of symmetry breaking

We know that P1Con, K1Con, P2Con and K2Con are all individually equal to zero always. Thus at a given steady state, [K2Con - P1Con] and [K1Con - P2Con] must also be equal to zero. We thus introduce terms T = (K2Con - P1Con) = 0 and Q = (K1Con - P2Con) = 0.

$T := (K2Con - P1Con) = 0$:
$Q := (K1Con - P2Con) = 0$:

Simplifying T = [K2Con - P1Con] using the *simplify* command, we get the following expression.

$simplify(T)$ $-(K2 - P1)\,(Ap\ c_2 + 1) = 0$

Since all kinetic constants and concentrations of variables are positive, in order for T to be equal to zero, [K2] is necessarily equal to [P1]. Using this information and simplifying Q = [K1Con - P2Con] = 0 we get the following expression.

$K2 := P1$:
$simplify(Q)$ $-K1 + P2 = 0$

Thus [K1] is equal to [P2] at any given steady state. Using this information, the earlier expression for [A] and [App] reduces to the following.

$K1 := P2$:

$$A = \frac{P1\ Ap\ c_2\ k_2}{c_1\ P2\ k_1}$$

$$App = \frac{P1\ Ap\ c_2\ k_2}{c_1\ P2\ k_1}$$

Thus we can see that irrespective of enzyme concentrations and kinetic parameters, [A] is always equal to [App]. i.e. There is no possibility of an asymmetric branch in this model implying infeasibility of symmetry breaking.

Triple Site Ordered Distributive Phosphorylation : Common Kinase Common Phosphatase
Case 1 symmetry - Present and Breaks

In this Maple file we analytically show the presence of symmetry breaking in ordered distributive TSP network with common kinase and common phosphatase effecting phosphorylation and dephoshorylation respectively. We do this by first describing the model as a system of ODEs and imposing the kinetic constraints pertaining to case 1 symmetry. We further describe the substrate and enzyme conservations associated with the model. By solving for the steady state of the system of ODEs we obtain relations between substrate variables (concentrations) in terms of each other and the free enzyme concentrations. In ordered distributive TSP (with common kinase common phosphatase), $K_{Total} = P_{Total}$ is required for exact case 1 symmetry to be present. We use this information to get a further simplified expression describing all possible steady states of the system. With case 1 symmetry in the ordered distributive MSP, the free enzyme kinase and phosphatase share a strict symmetry in the symmetric steady state ([K] = [P]). Thus by isolating steady states not of this type from the earlier correlation, we ascertain the features of the asymmetric state emerging from symmetry breaking. This procedure is carried out in detail below using built in Maple commands.

We initialize the Maple file with the *restart* command and load the relevant libraries of inbuilt Maple functions (*LinearAlgebra, VectorCalculus, Student[LinearAlgebra]*)

restart : *with*(*LinearAlgebra*) : *with*(*VectorCalculus*) : *with*(*Student*[*LinearAlgebra*]) :

The ordered distributive TSP system is modelled as a set of ODEs using the kinetic nomenclature described in the main text and supplementary figure (refer to Appendix 2 figure 10). Here dA represents d[A]/dt and similarly for other expressions. At steady state thus, each of the right hand sides of these expressions will be equal to zero.

$dA := k_{ub1} \cdot AK + k_6 \cdot ApP - k_{b1} \cdot A \cdot K :$
$dAp := k_1 \cdot AK + k_5 \cdot AppP + k_{ub2} \cdot ApK + k_{ub6} \cdot ApP - k_{b6} \cdot Ap \cdot P - k_{b2} \cdot Ap \cdot K :$
$dApp := k_2 \cdot ApK + k_4 \cdot ApppP + k_{ub3} \cdot AppK + k_{ub5} \cdot AppP - k_{b3} \cdot App \cdot K - k_{b5} \cdot App \cdot P :$
$dAppp := k_3 \cdot AppK + k_{ub4} \cdot ApppP - k_{b4} \cdot Appp \cdot P :$

$dAK := k_{b1} \cdot A \cdot K - (k_{ub1} + k_1) \cdot AK :$
$dApK := k_{b2} \cdot Ap \cdot K - (k_{ub2} + k_2) \cdot ApK :$
$dAppK := k_{b3} \cdot App \cdot K - (k_{ub3} + k_3) \cdot AppK :$
$dApppP := k_{b4} \cdot Appp \cdot P - (k_{ub4} + k_4) \cdot ApppP :$
$dAppP := k_{b5} \cdot App \cdot P - (k_{ub5} + k_5) \cdot AppP :$
$dApP := k_{b6} \cdot Ap \cdot P - (k_{ub6} + k_6) \cdot ApP :$

$dK := -k_{b1} \cdot A \cdot K - k_{b2} \cdot Ap \cdot K - k_{b3} \cdot App \cdot K + (k_{ub1} + k_1) \cdot AK + (k_{ub2} + k_2) \cdot ApK + (k_{ub3} + k_3)$
$\cdot AppK :$
$dP := -k_{b4} \cdot Appp \cdot P - k_{b5} \cdot App \cdot P - k_{b6} \cdot Ap \cdot P + (k_{ub4} + k_4) \cdot ApppP + (k_{ub5} + k_5) \cdot AppP + (k_{ub6}$

$$+ k_6)\cdot ApP:$$

The above equations are also associated with conservation conditions which are described below. Here we store the conservation expressions as ACon, PCon and KCon for the substrate and the respective enzymes. Each of these expressions is always equal to zero (both in the transient behavior and at steady state).

$$ACon := A_{Total} - A - Ap - App - Appp - AK - ApP - AppP - ApppP - ApK - AppK:$$
$$KCon := K_{Total} - K - AK - ApK - AppK:$$
$$PCon := P_{Total} - P - ApP - AppP - ApppP:$$

Kinetic constraints for case 1 symmetry (refer main text) are imposed on the original model.

$$k_4 := k_1 : k_{ub4} := k_{ub1} : k_{b4} := k_{b1} :$$
$$k_5 := k_2 : k_{ub5} := k_{ub2} : k_{b5} := k_{b2} :$$
$$k_6 := k_3 : k_{ub6} := k_{ub3} : k_{b6} := k_{b3} :$$

In addition to the kinetic constraints the total enzyme concentrations of kinase and phosphatase need to be equal for exact case 1 symmetry to be present. This is imposed as shown below.

$$K_{Total} := P_{Total} :$$

At this stage we introduce auxiliary constants c_1, c_2 and c_3 in place of the binding constants so as to make further analytical expressions more accessible.

$$k_{b1} := c_1\cdot(k_1 + k_{ub1}) :$$
$$k_{b2} := c_2\cdot(k_2 + k_{ub2}) :$$
$$k_{b3} := c_3\cdot(k_3 + k_{ub3}) :$$

Once this is done, we solve for the steady state of the system in terms of fewer key variables. In this context we want to solve all variables in terms of the concentrations of the free enzymes ([K] & [P]) and the concentration of the partially modified substrate ([Ap]). In order to do this, we use the Maple command *solve*, which solves the equation supplied for a given variable. We first solve for the individual complexes using their corresponding differential equation. An example of this (using [AK]) is given below in detail.

The differential equation of [AK] is given by,

$$\frac{d\,[AK]}{dt} = dAK \qquad \frac{d\,[AK]}{dt} = c_1\,(k_{ub1} + k_1)\,A\,K - (k_{ub1} + k_1)\,AK$$

The *solve* command by Maple, solves this equation for the given variable (in this case [AK]). We in turn store this value (the solution returned by the *solve* command) in [AK]. This is performed by the following command.

$AK := solve(dAK, AK)$

$$AK := K\, A\, c_1 \quad (1)$$

This operation is repeated for the other complexes and substrate forms as well. Here we simultaneously solve expressions for the substrate forms using the *solve* command as shown below. The solution is stored in a variable labelled *Sol*, and then the respective solutions of the substrate concentrations are extracted from this vector using the *eval* command.

$ApK := solve(dApK, ApK)$:
$AppK := solve(dAppK, AppK)$:
$ApP := solve(dApP, ApP)$:
$AppP := solve(dAppP, AppP)$:
$ApppP := solve(dApppP, ApppP)$:

$Sol := solve(\{dA, dAppp, dApp\}, \{A, Appp, App\})$:

$A := eval(A, Sol)$:
$Ap := eval(Ap, Sol)$:
$App := eval(App, Sol)$:
$Appp := eval(Appp, Sol)$:

Doing this results in the following correlations between the concentrations of the various substrate forms at steady state.

$$A = \frac{P\, Ap\, c_3\, k_3}{K\, c_1\, k_1}$$

$$Ap = Ap$$

$$App = \frac{Ap\, K}{P}$$

$$Appp = \frac{Ap\, K^2\, c_3\, k_3}{P^2\, c_1\, k_1}$$

Proof for invariant in the asymmetric branches

We know that PCon and KCon are both individually equal to zero always. Thus at a given steady state, PCon - KCon must also be equal to zero.

Here we introduce the term T = (KCon - PCon) = 0 and also introduce a new ratio, ϵ = [K]/[P]. Note: As discussed in the main text, the symmetric steady state is one where [K] = [P] or ϵ = 1. Since we are isolating solutions of asymmetry, we are primarily interested in solutions that permit, $\epsilon \neq 1$.

$T := KCon - PCon = 0$:
$K := \epsilon \cdot P$:

The following command (*simplify*), simplifies the expression T algebraically as shown below

$$simplify(T) = -\frac{\left(Ap\ (\epsilon+1)\ (k_1-k_3)\ c_3+k_1\right)\ P\ (\epsilon-1)}{k_1} = 0$$

From this we can ascertain that, should an asymmetric steady state exist (where $\epsilon \neq 1$) - the expression $Ap\ (\epsilon+1)\ (k_1-k_3)\ c_3+k_1$ needs to be zero. This term is an expression involving of the concentrations of the partial substrate form, ϵ and kinetic constants. Thus solving this to isolate the partial substrate form in terms of the kinetic parameters and ϵ, we get the following. Here we use the *solve* command from Maple to solve T for [Ap].

$$Ap := solve(T, Ap) = -\frac{k_1}{(\epsilon k_1-\epsilon k_3+k_1-k_3)\ c_3}$$

Using this information, we evaluate [App] from the correlations obtained earlier

$$App = -\frac{k_1\ \epsilon}{(\epsilon k_1-\epsilon k_3+k_1-k_3)\ c_3}$$

We can see a pattern here. Adding [Ap] and [App] we get,

$simplify(Ap + App)$

$$-\frac{k_1}{c_3\ (k_1-k_3)} \qquad (1.1)$$

Thus we can see that in an asymmetric steady state, the sum of the partially modified substrates is fixed and is given by the above expression involving only a few key kinetic parameters.

Necessary conditions

Since substrate concentrations are always positive, the expression in equation 1.1 should be positive. This gives us the necessary condition for an asymmetric state to exist as follows.

1. $k_3 > k_1$

Sufficiency of necessary conditions

In this section of the proof we show the necessary conditions shown above are also sufficient for an asymmetric steady state to exist for some positive A_{Total} value. i.e. We show that upon a bifurcation along A_{Total} we are bound to encounter symmetry breaking provided the necessary conditions are satisfied. Note that a feasible steady state in this context is one in which the concentrations of all substrates, complexes and enzymes are positive.

We do this by showing that the asymmetric states defined by the invariant concentration of [Ap] and [App] described above is indeed a feasible solution for the system of ODEs at some positive A_{Total} value.

In an asymmetric steady state, as seen above the concentration of [Ap] and [App] are fixed by a few kinetic constants,

$$Ap := simplify\left(-\frac{k_1}{(\epsilon k_1 - \epsilon k_3 + k_1 - k_3)\, c_3}\right) = -\frac{k_1}{(\epsilon + 1)\,(k_1 - k_3)\, c_3}$$

$$App := simplify\left(-\frac{k_1\, \epsilon}{(\epsilon k_1 - \epsilon k_3 + k_1 - k_3)\, c_3}\right) = -\frac{k_1\, \epsilon}{(\epsilon + 1)\,(k_1 - k_3)\, c_3}$$

The other variables in this asymmetric state are thus given by (we obtain this by using the correlation obtained earlier between the different concentrations)

$$A = -\frac{k_3}{(\epsilon + 1)\,(k_1 - k_3)\, \epsilon\, c_1}$$

$$Appp = -\frac{\epsilon^2\, k_3}{(\epsilon + 1)\,(k_1 - k_3)\, c_1}$$

$$AK = -\frac{P\, k_3}{(\epsilon + 1)\,(k_1 - k_3)}$$

$$ApK = -\frac{\epsilon\, P\, k_1\, c_2}{(\epsilon + 1)\,(k_1 - k_3)\, c_3}$$

$$AppK = -\frac{\epsilon^2\, P\, k_1}{(\epsilon + 1)\,(k_1 - k_3)}$$

$$ApppP = -\frac{P\, \epsilon^2\, k_3}{(\epsilon + 1)\,(k_1 - k_3)}$$

$$AppP = -\frac{\epsilon\, P\, k_1\, c_2}{(\epsilon + 1)\,(k_1 - k_3)\, c_3}$$

$$ApP = -\frac{P\, k_1}{(\epsilon + 1)\,(k_1 - k_3)}$$

The system of ODE is also satisfied at this point, as is verified below.

$simplify(dA) = 0$
$simplify(dAp) = 0$
$simplify(dApp) = 0$
$simplify(dAppp) = 0$
$simplify(dAK) = 0$
$simplify(dApK) = 0$

$simplify(dAppK) = 0$
$simplify(dApppP) = 0$
$simplify(dAppP) = 0$
$simplify(dApP) = 0$
$simplify(dK) = 0$
$simplify(dP) = 0$

Hence all that remains to be shown is that the variables (as described above) are positive for some value of A_{Total}.

This is true if and only if

1. **Necessary condition $(k_3 > k_1)$ is satisfied**
2. **P and ϵ are positive.**

Now by using the total conservation of phosphatase in the system (PCon = 0), we obtain an algebraic expression for [P] in terms of P_{Total}, kinetic constants and ϵ as shown below. The Maple command *solve* is used for this purpose as shown below.

$P := simplify(solve(PCon, P))$

$$P := -\frac{P_{Total}\,(\epsilon+1)\,(k_1-k_3)\,c_3}{\epsilon^2\,k_3\,c_3+\left(\left(-k_1+k_3\right)c_3+c_2\,k_1\right)\epsilon+c_3\,k_3} \tag{3.1}$$

Hence if ϵ is positive P is automatically positive (provided necessary conditions are satisfied).

Thus this means that for every positive value of $\epsilon \neq 1$, all concentrations are positive and the conservation of kinase and phosphatase is also satisfied.

Since the concentrations are all positive, there exists a unique A_{Total} value for every ϵ (permitting asymmetric states).

Hence we have proved that symmetry breaking is guaranteed for some finite positive A_{Total}, provided the necessary conditions above are satisfied - making those conditions sufficient for the behavior.

Prediction of pitchfork bifurcation along A_{Total}

Here we predict the value of A_{Total} at which symmetry breaking occurs via a pitch fork bifurcation. This point in the bifurcation is characterized by the intersection of both the symmetric steady state branch and the asymmetric steady state branches.

Hence at the position of symmetry breaking, we know two insights.

1. The system is still symmetric, hence [A] = [Appp], [Ap] = [App] and [K] = [P].
2. The invariant describing the asymmetric steady state is also true.

Using these two information, we can simplify the original system considerably as follows.

$K := P : \epsilon := 1 :$

$$Ap := -\frac{k_1}{(\epsilon + 1)\,(k_1 - k_3)\,c_3} :$$

$$App := -\frac{k_1\,\epsilon}{(\epsilon + 1)\,(k_1 - k_3)\,c_3} :$$

Now, by solving the conservation expression for the substrate we can isolate the value of A_{Total} when the asymmetric steady states and the symmetric steady state intersect (indicating the pitchfork bifurcation point)

$A_{Total} = (collect(solve(ACon, A_{Total}), P_{Total}))$

$$A_{Total} = \frac{\left(2\,c_1\,c_2\,c_3\,k_1^2 - 2\,c_1\,c_2\,c_3\,k_1\,k_3 + 2\,c_1\,c_3^2\,k_1^2 - 2\,c_1\,c_3^2\,k_3^2\right) P_{Total}}{(k_1 - k_3)\,c_1\,c_3\,(c_2\,k_1 - k_1\,c_3 + 3\,c_3\,k_3)} + \frac{-c_1\,c_2\,k_1^2 + c_1\,c_3\,k_1^2 - 3\,c_1\,c_3\,k_1\,k_3 - c_2\,c_3\,k_1\,k_3 + c_3^2\,k_1\,k_3 - 3\,c_3^2\,k_3^2}{(k_1 - k_3)\,c_1\,c_3\,(c_2\,k_1 - k_1\,c_3 + 3\,c_3\,k_3)} \quad \textbf{(4.1)}$$

A cross verification of this analytical work is carried out in the read me file for the parameters used in generating the figure (Appendix 2 figure 8).

Random DSP System 1 : Common Kinase Common Phosphatase

Case 1	Case 2	Case 3
**Present and Breaks Invariant identified**	**Present and Can't Break**	**Present and Breaks Invariant Identified**

In this Maple file we analytically show the presence of case 1 and case 3 symmetry breaking in Random DSP network with common kinase and common phosphatase (System 1) effecting phosphorylation and dephoshorylation respectively. We also show the infeasibility of case 2 symmetry breaking in this network.

In each case, we do this by first describing the model as a system of ODEs along with the associated substrate and enzyme conservations. We then impose the kinetic constraints (and constraints on total enzyme concentrations) pertaining to the specific case of symmetry. By algebraically solving for the steady state of the resulting system of ODEs we obtain relations between concentrations of the substrate variables in terms of each other and the free enzyme concentrations. After this, we identify key symmetric pairings that represent the symmetric steady state. i.e. In Random System 1, case 1 symmetry requires symmetry between [K] & [P] and [A00] & [A11]; case 2 symmetry breaking requires symmetry between [A01] & [A10]; case 3 symmetry breaking requires symmetry between [K] & [P], [A01] & [A10] and [A00] & [A11]. In each case by leveraging this insight and isolating steady states not of this type, we ascertain the features of the asymmetric state emerging through symmetry breaking. In a similar way we show the infeasibility of case 2 symmetry breaking by revealing that [A01] and [A10] are always equal for any given feasible steady state. These procedures are carried out in detail below using built in Maple commands.

Note: A subscript is used to distinguish between the two different complexes formed between [K] and [A00] ([$A00K_1$ and $A00K_2$]). Similarly a subscript is used to distinguish between the two distinct complexes formed between [P] and [A11] ([$A11P_1$ and $A11P_2$]).

We initialize the Maple file with the *restart* command and load the relevant libraries of inbuilt Maple functions (*LinearAlgebra, VectorCalculus, Student[LinearAlgebra]*)

restart : *with*(*LinearAlgebra*) : *with*(*VectorCalculus*) : *with*(*Student*[*LinearAlgebra*]) :

The system is modelled as a set of ODEs using the kinetic nomenclature described in the main text and supplementary figure (refer to Appendix 2 figure 10). Here dA00 represents d[A00]/dt and similarly for other expressions. At steady state thus, each of the right hand sides of these expressions will be equal to zero.

$$dA00 := k_4 \cdot A01P + a_4 \cdot A10P + k_{ub1} \cdot A00K_1 + a_{ub1} \cdot A00K_2 - k_{b1} \cdot A00 \cdot K - a_{b1} \cdot A00 \cdot K :$$
$$dA01 := k_1 \cdot A00K_1 + k_3 \cdot A11P_1 + k_{ub2} \cdot A01K + k_{ub4} \cdot A01P - k_{b2} \cdot A01 \cdot K - k_{b4} \cdot A01 \cdot P :$$
$$dA10 := a_1 \cdot A00K_2 + a_3 \cdot A11P_2 + a_{ub2} \cdot A10K + a_{ub4} \cdot A10P - a_{b2} \cdot A10 \cdot K - a_{b4} \cdot A10 \cdot P :$$
$$dA11 := k_2 \cdot A01K + a_2 \cdot A10K + k_{ub3} \cdot A11P_1 + a_{ub3} \cdot A11P_2 - k_{b3} \cdot A11 \cdot P - a_{b3} \cdot A11 \cdot P :$$

$$dA00K_1 := k_{b1} \cdot A00 \cdot K - \left(k_1 + k_{ub1}\right) \cdot A00K_1 :$$

$dA01K := k_{b2} \cdot A01 \cdot K - (k_2 + k_{ub2}) \cdot A01K :$
$dA00K_2 := a_{b1} \cdot A00 \cdot K - (a_{ub1} + a_1) \cdot A00K_2 :$
$dA10K := a_{b2} \cdot A10 \cdot K - (a_{ub2} + a_2) \cdot A10K :$
$dA11P_1 := k_{b3} \cdot A11 \cdot P - (k_{ub3} + k_3) \cdot A11P_1 :$
$dA01P := k_{b4} \cdot A01 \cdot P - (k_{ub4} + k_4) \cdot A01P :$
$dA11P_2 := a_{b3} \cdot A11 \cdot P - (a_{ub3} + a_3) \cdot A11P_2 :$
$dA10P := a_{b4} \cdot A10 \cdot P - (a_{ub4} + a_4) \cdot A10P :$

$dK := -k_{b1} \cdot A00 \cdot K + (k_1 + k_{ub1}) \cdot A00K_1 - a_{b1} \cdot A00 \cdot K + (a_{ub1} + a_1) \cdot A00K_2 - k_{b2} \cdot A01 \cdot K + (k_2 + k_{ub2}) \cdot A01K - a_{b2} \cdot A10 \cdot K + (a_{ub2} + a_2) \cdot A10K :$
$dP := -k_{b3} \cdot A11 \cdot P + (k_{ub3} + k_3) \cdot A11P_1 - a_{b3} \cdot A11 \cdot P + (a_{ub3} + a_3) \cdot A11P_2 - k_{b4} \cdot A01 \cdot P + (k_{ub4} + k_4) \cdot A01P - a_{b4} \cdot A10 \cdot P + (a_{ub4} + a_4) \cdot A10P :$

The above equations are also associated with conservation conditions which are described below. Here we store the conservation expressions as ACon, PCon and KCon for the substrate and the respective enzymes. Each of these expressions is always equal to zero (both in the transient behavior and at steady state).

$ACon := A_{Total} - A00 - A10 - A01 - A11 - A00K_1 - A01K - A00K_2 - A10K - A11P_1 - A10P - A11P_2 - A01P :$
$KCon := K_{Total} - K - A00K_1 - A10K - A00K_2 - A01K :$
$PCon := P_{Total} - P - A11P_1 - A10P - A11P_2 - A01P :$

Until now we have modelled the System 1 Random DSP network with common kinase effecting phosphorylation and a common phosphatase for dephosphorylation - without any impositions on kinetics or total concentrations of enzymes. In the following segments, we specifically do this for each class of symmetry. The codes for each symmetry are modular and in order to run a particular symmetry, please run the code until this point and then run only the code for the specific class of symmetry.
Note: Do not run the whole script at the same time, as this will impose all symmetries at the same time and give incorrect results.

Case 1 Symmetry : Present and Breaks

Kinetic constraints for case 1 symmetry (refer main text) are imposed on the original model.

$k_3 := k_1 : k_{b3} := k_{b1} : k_{ub3} := k_{ub1} :$
$k_4 := k_2 : k_{b4} := k_{b2} : k_{ub4} := k_{ub2} :$

$a_3 := a_1 : a_{b3} := a_{b1} : a_{ub3} := a_{ub1} :$
$a_4 := a_2 : a_{b4} := a_{b2} : a_{ub4} := a_{ub2} :$

In addition to the kinetic constraints the total enzyme concentrations of kinase and phosphatase need to be equal for exact case 1 symmetry to be present. This is imposed as shown below.

$K_{Total} := P_{Total}:$

At this stage we introduce auxiliary constants c_1, c_2, d_1 and d_2 in place of the binding constants so as to make further analytical expressions more accessible.

$k_{b1} := c_1 \cdot (k_1 + k_{ub1}):$
$k_{b2} := c_2 \cdot (k_2 + k_{ub2}):$

$a_{b1} := d_1 \cdot (a_1 + a_{ub1}):$
$a_{b2} := d_2 \cdot (a_2 + a_{ub2}):$

Once this is done, we solve for the steady state of the system in terms of fewer key variables. In this context we want to solve all variables in terms of the concentrations of the free enzymes ([K] & [P]) and concentration of the partially modified substrate ([A10]). In order to do this, we use the Maple command *solve,* which solves the equation supplied for a given variable. We first solve for the individual complexes using their corresponding differential equation. An example of this (using [A00K$_1$]) is given below in detail.

The differential equation of [A00K$_1$] is given by,

$$\frac{d\,[A00K_1]}{dt} = dA00K_1 \qquad \frac{d\,[A00K_1]}{dt} = c_1\,(k_1 + k_{ub1})\,A00\,K - (k_1 + k_{ub1})\,A00K_1$$

The *solve* command by Maple, solves this equation for the given variable (in this case [A00K$_1$]). We in turn store this value (the solution returned by the *solve* command) in [A00K$_1$]. This is performed by the following command.

$A00K_1 := solve(dA00K_1, A00K_1)$

$$A00K_1 := K\,A00\,c_1 \tag{1.1}$$

This operation is performed for the other complexes and substrate forms as well. Here we simultaneously solve expressions for the substrate forms using the *solve* command as shown below. The solution is stored in a variable labelled *Sol*, and then the respective solutions are extracted from this vector using the *eval* command.

$A10K := solve(dA10K, A10K):$
$A00K_2 := solve(dA00K_2, A00K_2):$
$A01K := solve(dA01K, A01K):$
$A11P_1 := solve(dA11P_1, A11P_1):$

$A11P_2 := solve(dA11P_2, A11P_2) :$
$A01P := solve(dA01P, A01P) :$
$A10P := solve(dA10P, A10P) :$

$Sol := solve(\{dA00, dA01, dA11\}, \{A00, A01, A11\}) :$

$A00 := eval(A00, Sol) :$
$A01 := eval(A01, Sol) :$
$A11 := eval(A11, Sol) :$

Doing this results in the following correlations between the concentrations of the various substrate forms at steady state.

$$A00 = \frac{A10\, d_2\, a_2\, P}{a_1\, d_1\, K}$$
$$A01 = \frac{A10\, d_2\, a_2\, c_1\, k_1}{a_1\, d_1\, c_2\, k_2}$$
$$A10 = A10$$
$$A11 = \frac{K\, A10\, d_2\, a_2}{P\, a_1\, d_1}$$

Proof for invariant in the asymmetric branches

We know that PCon and KCon are both individually equal to zero always. Thus at a given steady state, PCon - KCon must also be equal to zero.

We thus introduce the term T = (KCon - PCon) = 0 and also introduce a new ratio, ϵ = [K]/[P]. Note: As discussed in the main text, the symmetric steady state is one where [K] = [P] or ϵ = 1. Since we are isolating solutions of asymmetry, we are primarily interested in solutions that permit, $\epsilon \neq 1$.

$T := KCon - PCon = 0 :$
$K := \epsilon \cdot P :$

The following command (*simplify*), simplifies the expression

$simplify(T) =$
$$-\frac{(\epsilon - 1)\left(\left(\left(\left(a_1 - a_2\right) d_1 - a_2\, c_1\right) A10\, d_2 + a_1\, d_1\right) k_2 + A10\, a_2\, c_1\, d_2\, k_1\right) P}{a_1\, d_1\, k_2} = 0$$

From this we can ascertain that, should an asymmetric steady state exist (where $\epsilon \neq 1$) - the term ($\left(A10\left(\left(k_1 - k_2\right) c_1 - d_1\, k_2\right) c_2 + c_1\, k_1\right) p_2 + A10\, c_2\, d_1\, k_2\, p_1$) in the expression needs to be zero. We note that this term is a expression in the partial substrate form and kinetic

constants. Thus solving this to isolate the partial substrate form we get the following. Here we use the *solve* command from Maple to solve T for [A10] as shown below.

$A10 := simplify(solve(T, A10))$

$$A10 := -\frac{a_1 d_1 k_2}{d_2\left(\left(\left(-c_1 - d_1\right) a_2 + a_1 d_1\right) k_2 + a_2 c_1 k_1\right)} \quad (1.1.1)$$

We can thus see that in an asymmetric state [A10] is always a constant given by only a few key kinetic parameters. Using this expression back in the relation between [A01] we ascertain that [A01] is also fixed at a constant concentration at an asymmetric state.

$A01 := simplify(A01)$

$$A01 := -\frac{a_2 c_1 k_1}{\left(\left(\left(k_1 - k_2\right) c_1 - d_1 k_2\right) a_2 + a_1 d_1 k_2\right) c_2} \quad (1.1.2)$$

Thus we can see that in an asymmetric steady state, the value of the partially modified substrates ([A01] and [A10]) is fixed and is given by the above expressions involving only a few key kinetic parameters.

Necessary Conditions

Since substrate concentration are always necessarily positive, the expression in equation 1.1.1 and 1.1.2 should be positive. The numerator is only a function of kinetic parameters which are always positive, thus the denominator must necessarily be negative to ensure that the resulting concentration is positive. This gives us the necessary condition for the asymmetric state to exist as follows.

1. $c_1 a_2 (k_2 - k_1) + d_1 k_2 (a_2 - a_1) > 0$

Sufficiency of necessary conditions

In this section of the proof we show the necessary conditions shown above are also sufficient for an asymmetric steady state to exist for some positive A_{Total} value. or upon a bifurcation along A_{Total} we are bound to encounter symmetry breaking provided the necessary conditions are satisfied. Note that in the context of this system, a feasible steady state is one in which the concentrations of all substrates, complexes and enzymes are positive.

We do this by showing that an asymmetric state defined by the invariant concentrations described above indeed is a solution for the system of ODEs at some positive A_{Total} value.

In an asymmetric steady state, as seen above [A01] and [A10] are fixed by a few kinetic constants,

$$A10 := expand\left(-\frac{a_1 d_1 k_2}{d_2 \left(\left(\left(-c_1 - d_1\right) a_2 + a_1 d_1\right) k_2 + a_2 c_1 k_1\right)}\right) =$$

$$-\frac{a_1 d_1 k_2}{\left(a_1 d_1 k_2 + a_2 c_1 k_1 - a_2 c_1 k_2 - a_2 d_1 k_2\right) d_2}$$

$$A01 := expand\left(-\frac{a_2 c_1 k_1}{c_2 \left(\left(\left(k_1 - k_2\right) c_1 - d_1 k_2\right) a_2 + a_1 d_1 k_2\right)}\right) =$$

$$-\frac{a_2 c_1 k_1}{\left(a_1 d_1 k_2 + a_2 c_1 k_1 - a_2 c_1 k_2 - a_2 d_1 k_2\right) c_2}$$

The other variables in this asymmetric state are thus given by

$$expand(A00) = -\frac{k_2 a_2}{\left(a_1 d_1 k_2 + a_2 c_1 k_1 - a_2 c_1 k_2 - a_2 d_1 k_2\right) \epsilon}$$

$$expand(A11) = -\frac{\epsilon k_2 a_2}{a_1 d_1 k_2 + a_2 c_1 k_1 - a_2 c_1 k_2 - a_2 d_1 k_2}$$

$$expand(A00K_1) = -\frac{P k_2 a_2 c_1}{a_1 d_1 k_2 + a_2 c_1 k_1 - a_2 c_1 k_2 - a_2 d_1 k_2}$$

$$expand(A00K_2) = -\frac{P k_2 a_2 d_1}{a_1 d_1 k_2 + a_2 c_1 k_1 - a_2 c_1 k_2 - a_2 d_1 k_2}$$

$$expand(A01K) = -\frac{\epsilon P a_2 c_1 k_1}{a_1 d_1 k_2 + a_2 c_1 k_1 - a_2 c_1 k_2 - a_2 d_1 k_2}$$

$$expand(A10K) = -\frac{\epsilon P a_1 d_1 k_2}{a_1 d_1 k_2 + a_2 c_1 k_1 - a_2 c_1 k_2 - a_2 d_1 k_2}$$

$$expand(A11P_1) = -\frac{P \epsilon k_2 a_2 c_1}{a_1 d_1 k_2 + a_2 c_1 k_1 - a_2 c_1 k_2 - a_2 d_1 k_2}$$

$$expand(A11P_2) = -\frac{P \epsilon k_2 a_2 d_1}{a_1 d_1 k_2 + a_2 c_1 k_1 - a_2 c_1 k_2 - a_2 d_1 k_2}$$

$$expand(A01P) = -\frac{P a_2 c_1 k_1}{a_1 d_1 k_2 + a_2 c_1 k_1 - a_2 c_1 k_2 - a_2 d_1 k_2}$$

$$expand(A10P) = -\frac{P a_1 d_1 k_2}{a_1 d_1 k_2 + a_2 c_1 k_1 - a_2 c_1 k_2 - a_2 d_1 k_2}$$

The system of ODE is also satisfied at this point, as is verified below.

$simplify(dA00) = 0$
$simplify(dA11) = 0$
$simplify(dA01) = 0$

$simplify(dA10) = 0$
$simplify(dA00K_1) = 0$
$simplify(dA00K_2) = 0$
$simplify(dA01P) = 0$
$simplify(dA10P) = 0$
$simplify(dA01K) = 0$
$simplify(dA10K) = 0$
$simplify(dA11P_1) = 0$
$simplify(dA11P_2) = 0$
$simplify(dP) = 0$
$simplify(dK) = 0$

Hence all that remains to be shown is that the variables (as described above) are positive for some value of A_{Total}.
This is true if and only if

1. **Necessary condition is satisfied**
2. **P and ϵ are positive.**

Now by using the total conservation of phosphatase in the system (PCon = 0), we obtain an algebraic expression for [P] in terms of P_{Total}, kinetic constants and ϵ as shown below. The Maple command *solve* is used for this purpose as shown below.

$P := expand(solve(PCon, P))$

$$P := -\frac{P_{Total}\, a_1\, d_1}{a_2\,(c_1\,\epsilon + d_1\,\epsilon + c_1 + d_1)} - \frac{P_{Total}\, c_1\, k_1}{k_2\,(c_1\,\epsilon + d_1\,\epsilon + c_1 + d_1)} + \frac{P_{Total}\, c_1}{c_1\,\epsilon + d_1\,\epsilon + c_1 + d_1} + \frac{P_{Total}\, d_1}{c_1\,\epsilon + d_1\,\epsilon + c_1 + d_1} \qquad \textbf{(1.3.1)}$$

$simplify(P)$

$$-\frac{\left(\left((k_1 - k_2)\, c_1 - d_1\, k_2\right) a_2 + a_1\, d_1\, k_2\right) P_{Total}}{a_2\, k_2\,(\epsilon + 1)\,(c_1 + d_1)} \qquad \textbf{(1.3.2)}$$

Hence if ϵ is positive P is automatically positive (provided necessary conditions are satisfied) . Hence we have shown that the individual conservation equations for the enzymes (KCon and PCon) and the system of ODEs is solved by the expressions above, and represent feasible solutions provided ϵ is positive.

Since the concentrations are all positive there exists a unique A_{Total} value for every ϵ (permitting asymmetric states).

Hence we have proved that symmetry breaking is guaranteed for some finite positive A_{Total}, provided the necessary conditions above are satisfied - making those conditions sufficient for the behavior.

Prediction of pitchfork bifurcation along A_{Total}

Here we predict the value of A_{Total} at which symmetry breaking occurs via a pitch fork bifurcation. This point in the bifurcation is characterized by the intersection of both the symmetric steady state branch and the asymmetric steady state branches.

Hence at the position of symmetry breaking, we know two insights.

1. The system is still symmetric, hence [A00] = [A11] and [K] = [P].
2. The invariant describing the asymmetric steady state is also true.

Using these two information, we can simplify the original system considerably as follows.

$$K := P : \epsilon := 1 :$$

$$A10 := -\frac{a_1 d_1 k_2}{d_2 \left(\left(\left(-c_1 - d_1 \right) a_2 + a_1 d_1 \right) k_2 + a_2 c_1 k_1 \right)} :$$

Now, by solving the conservation expression for the substrate we can isolate the value of A_{Total} when the asymmetric steady states and the symmetric steady state intersect (indicating the pitchfork bifurcation point).

$$A_{Total} = simplify\left(solve\left(ACon, A_{Total}\right)\right)$$

$$A_{Total} = \left(d_2 \left(-c_2 \left(c_1 + d_1 \right) \left(P_{Total} c_1 + P_{Total} d_1 + 2 \right) k_2^2 - c_1 k_1 \left(c_1 + d_1 \right) k_2 + c_1^2 c_2 k_1^2 P_{Total} \right) a_2^2 + 2 d_1 \left(\left(-\frac{c_1}{2} - \frac{d_1}{2} \right) k_2 + c_1 d_2 k_1 P_{Total} \right) c_2 k_2 a_1 a_2 + a_1^2 c_2 d_1^2 d_2 k_2^2 P_{Total} \right) \Big/ \left(\left(c_1 + d_1 \right) a_2 c_2 \left(\left(\left(-c_1 - d_1 \right) k_2 + c_1 k_1 \right) a_2 + a_1 d_1 k_2 \right) d_2 k_2 \right) \quad (1.4.1)$$

A cross verification of this analytical work is carried out in the read me file for the parameters used in generating the figures (Fig 2B).

Case 2 Symmetry : Present and Can't Break

Kinetic constraints for case 2 symmetry (refer main text) are imposed on the original model.

$a_1 := k_1 : a_{b1} := k_{b1} : a_{ub1} := k_{ub1} :$
$a_2 := k_2 : a_{b2} := k_{b2} : a_{ub2} := k_{ub2} :$
$a_3 := k_3 : a_{b3} := k_{b3} : a_{ub3} := k_{ub3} :$
$a_4 := k_4 : a_{b4} := k_{b4} : a_{ub4} := k_{ub4} :$

There are no constraints on the total enzyme concentrations for case 2 symmetry to be present. At this stage we introduce auxiliary constants c_1, c_2, c_3 and c_4 in place of the binding constants so as to make further analytical expressions more accessible.

$k_{b1} := c_1 \cdot (k_1 + k_{ub1}) :$
$k_{b2} := c_2 \cdot (k_2 + k_{ub2}) :$
$k_{b3} := c_3 \cdot (k_3 + k_{ub3}) :$
$k_{b4} := c_4 \cdot (k_4 + k_{ub4}) :$

Once this is done, we solve for the steady state of the system in terms of fewer key variables. In this context we want to solve all variables in terms of the concentrations of the free enzymes ([K] & [P]) and concentration of the completely unmodified substrate ([A00]). In order to do this, we use the Maple command *solve,* which solves the equation supplied for a given variable. We first solve for the individual complexes using their corresponding differential equation. An example of this (using [A00K$_1$]) is given below in detail.

The differential equation of [A00K$_1$] is given by,

$$\frac{d\,[A00K_1]}{dt} = dA00K_1$$

$$\frac{d\,[A00K_1]}{dt} = c_1\,(k_1 + k_{ub1})\,A00\,K - (k_1 + k_{ub1})\,A00K_1 \quad \textbf{(2.1)}$$

The *solve* command by Maple, solves this equation for the given variable (in this case [A00K$_1$]). We in turn store this value (the solution returned by the *solve* command) in [A00K$_1$]. This is performed by the following command.

$A00K_1 := solve(dA00K_1, A00K_1)$

$$A00K_1 := K\,A00\,c_1 \quad \textbf{(2.2)}$$

This operation is performed for the other complexes and substrate forms as well. Here we

simultaneously solve expressions for the substrate forms using the *solve* command as shown below. The solution is stored in a variable labelled *Sol*, and then the respective solutions are extracted from this vector using the *eval* command.

$A10K := solve(dA10K, A10K) :$
$A00K_2 := solve(dA00K_2, A00K_2) :$
$A01K := solve(dA01K, A01K) :$
$A11P_1 := solve(dA11P_1, A11P_1) :$
$A11P_2 := solve(dA11P_2, A11P_2) :$
$A01P := solve(dA01P, A01P) :$
$A10P := solve(dA10P, A10P) :$

$Sol := solve(\{dA10, dA01, dA11\}, \{A10, A01, A11\}) :$

$A10 := eval(A10, Sol) :$
$A01 := eval(A01, Sol) :$
$A11 := eval(A11, Sol) :$

Doing this results in the following correlations between the concentrations of the various substrate forms at steady state.

$$A00 = A00$$
$$A01 = \frac{K\, A00\, c_1\, k_1}{c_4\, k_4\, P}$$
$$A10 = \frac{K\, A00\, c_1\, k_1}{c_4\, k_4\, P}$$
$$A11 = \frac{A00\, K^2\, c_1\, c_2\, k_1\, k_2}{c_4\, k_4\, P^2\, c_3\, k_3}$$

Proof for impossibility of symmetry breaking

Thus from this we can clearly see that irrespective of kinetic parameters, the concentration of [A01] is always going to be equal to the concentration of [A10]. Thus there is no scope for any asymmetric steady state or case 2 symmetry breaking.

$$A01 = \frac{K\, A00\, c_1\, k_1}{c_4\, k_4\, P}$$

$$A10 = \frac{K\, A00\, c_1\, k_1}{c_4\, k_4\, P}$$

Case 3 Symmetry : Present and Breaks

Kinetic constraints for case 3 symmetry to be seen (refer main text) are imposed on the original model.

$a_3 := k_1 : a_{b3} := k_{b1} : a_{ub3} := k_{ub1} :$
$a_4 := k_2 : a_{b4} := k_{b2} : a_{ub4} := k_{ub2} :$
$a_1 := k_3 : a_{b1} := k_{b3} : a_{ub1} := k_{ub3} :$
$a_2 := k_4 : a_{b2} := k_{b4} : a_{ub2} := k_{ub4} :$

In addition to the kinetic constraints the total enzyme concentrations of kinase and phosphatase also need to be equal for exact case 3 symmetry to be present. This is imposed as shown below.

$K_{Total} := P_{Total} :$

At this stage we introduce auxiliary constants c_1, c_2, c_3 and c_4 in place of the binding constants so as to make further analytical expressions more accessible.

$k_{b1} := c_1 \cdot (k_1 + k_{ub1}) :$
$k_{b2} := c_2 \cdot (k_2 + k_{ub2}) :$
$k_{b3} := c_3 \cdot (k_3 + k_{ub3}) :$
$k_{b4} := c_4 \cdot (k_4 + k_{ub4}) :$

Once this is done, we solve for the steady state of the system in terms of fewer key variables. In this context we want to solve all variables in terms of the concentrations of the free enzymes ([K] & [P]) and concentration of the fully modified substrate ([A11]). In order to do this, we use the Maple command *solve,* which solves the equation supplied for a given variable. We first solve for the individual complexes using their corresponding differential equation. An example of this (using [A00K$_1$]) is given below in detail.

The differential equation of [A00K$_1$] is given by,

$$\frac{d\left[A00K_1\right]}{dt} = dA00K_1$$

$$\frac{d\left[A00K_1\right]}{dt} = c_1\left(k_1 + k_{ub1}\right) A00\,K - \left(k_1 + k_{ub1}\right) A00K_1 \qquad (3.1)$$

The *solve* command by Maple, solves this equation for the given variable (in this case [A00K$_1$]). We in turn store this value (the solution returned by the *solve* command) in [A00K$_1$]. This is performed by the following command.

$A00K_1 := solve(dA00K_1, A00K_1)$

$$A00K_1 := K\,A00\,c_1 \tag{3.2}$$

This operation is performed for the other complexes and substrate forms as well. Here we simultaneously solve expressions for the substrate forms using the *solve* command as shown below. The solution is stored in a variable labelled *Sol*, and then the respective solutions are extracted from this vector using the *eval* command.

$A10K := solve(dA10K, A10K) :$
$A00K_2 := solve(dA00K_2, A00K_2) :$
$A01K := solve(dA01K, A01K) :$
$A11P_1 := solve(dA11P_1, A11P_1) :$
$A11P_2 := solve(dA11P_2, A11P_2) :$
$A01P := solve(dA01P, A01P) :$
$A10P := solve(dA10P, A10P) :$

$Sol := solve(\{dA00, dA01, dA10, dA11\}, \{A00, A01, A10, A11\}) :$

$A00 := eval(A00, Sol) :$
$A01 := eval(A01, Sol) :$
$A10 := eval(A10, Sol) :$
$A11 := eval(A11, Sol) :$

Doing this results in the following correlations between the concentrations of the various substrate forms at steady state.

$A00 =$

$$\frac{P^2\,A11\,\left(K\,c_1\,c_2^2\,k_1\,k_2^2 + K\,c_3\,c_4^2\,k_3\,k_4^2 + P\,c_1\,c_2\,c_4\,k_1\,k_2\,k_4 + P\,c_2\,c_3\,c_4\,k_2\,k_3\,k_4\right)}{K^2\,\left(K\,c_1\,c_2\,c_4\,k_1\,k_2\,k_4 + K\,c_2\,c_3\,c_4\,k_2\,k_3\,k_4 + P\,c_1\,c_2^2\,k_1\,k_2^2 + P\,c_3\,c_4^2\,k_3\,k_4^2\right)} \tag{3.3}$$

$A01 =$

$$\frac{P\,A11\,\left(K\,c_1\,c_3\,c_4\,k_1\,k_3\,k_4 + K\,c_3^2\,c_4\,k_3^2\,k_4 + P\,c_1^2\,c_2\,k_1^2\,k_2 + P\,c_1\,c_2\,c_3\,k_1\,k_2\,k_3\right)}{K\,\left(K\,c_1\,c_2\,c_4\,k_1\,k_2\,k_4 + K\,c_2\,c_3\,c_4\,k_2\,k_3\,k_4 + P\,c_1\,c_2^2\,k_1\,k_2^2 + P\,c_3\,c_4^2\,k_3\,k_4^2\right)} \tag{3.4}$$

$A10 =$

$$\frac{P\,\left(K\,c_1^2\,c_2\,k_1^2\,k_2 + K\,c_1\,c_2\,c_3\,k_1\,k_2\,k_3 + P\,c_1\,c_3\,c_4\,k_1\,k_3\,k_4 + P\,c_3^2\,c_4\,k_3^2\,k_4\right)\,A11}{K\,\left(K\,c_1\,c_2\,c_4\,k_1\,k_2\,k_4 + K\,c_2\,c_3\,c_4\,k_2\,k_3\,k_4 + P\,c_1\,c_2^2\,k_1\,k_2^2 + P\,c_3\,c_4^2\,k_3\,k_4^2\right)} \tag{3.5}$$

$A11 =$

$$A11 \tag{3.6}$$

Proof for invariant in the asymmetric branches

We know that PCon and KCon are both individually equal to zero always. Thus at a given steady state, PCon - KCon must also be equal to zero.

We thus introduce the term T = (KCon - PCon) = 0 and also introduce a new ratio, ϵ = [K]/[P]. Note: As discussed in the main text, the symmetric steady state is one where [K] = [P] or ϵ = 1. Since we are isolating solutions of asymmetry, we are primarily interested in solutions that permit, $\epsilon \neq 1$.

$T := KCon - PCon = 0 :$
$K := \epsilon \cdot P :$

The following command (*simplify*), simplifies the expression

$simplify(T)$

$$-\left(P\,(\epsilon-1)\left(c_1\,c_2^{\,2}\,\epsilon\,k_1\,k_2^{\,2}+c_4\left(c_1\,k_1+c_3\,k_3\right)\left(\left(\left(\epsilon^2-A11\left(c_1+c_3\right)\epsilon-A11\left(c_1+c_3\right)\right)k_4+A11\,c_1\,k_1\,(\epsilon+1)\right)k_2+k_4\,A11\,c_3\,k_3\,(\epsilon+1)\right)c_2+c_3\,c_4^{\,2}\,\epsilon\,k_3\,k_4^{\,2}\right)\right)\Big/\left(\epsilon\left(c_1\,c_2^{\,2}\,k_1\,k_2^{\,2}+k_4\,\epsilon\,c_4\,k_2\left(c_1\,k_1+c_3\,k_3\right)c_2+c_3\,c_4^{\,2}\,k_3\,k_4^{\,2}\right)\right)=0 \quad (3.1.1)$$

From this we can ascertain that, should an asymmetric steady state exist (where $\epsilon \neq 1$) - the term (

$$c_1\,c_2^{\,2}\,\epsilon\,k_1\,k_2^{\,2}+\left(c_1\,k_1+c_3\,k_3\right)c_4\left(\left(\left(\epsilon^2-A11\left(c_1+c_3\right)\epsilon-A11\left(c_1+c_3\right)\right)k_4+A11\,c_1\,k_1\,(\epsilon+1)\right)k_2+k_4\,A11\,c_3\,k_3\,(\epsilon+1)\right)c_2+c_3\,c_4^{\,2}\,\epsilon\,k_3\,k_4^{\,2}$$

) in the expression needs to be equal to zero. We note that this term is a expression in the fully modified [A11], ϵ and kinetic constants. Thus solving this to isolate the fully modified substrate form we get the following. Here we use the *solve* command from Maple to solve T for [A11] as shown below.

$$A11 := simplify(solve(T, A11)) = -\frac{\epsilon\left(c_1\,c_2^{\,2}\,k_1\,k_2^{\,2}+k_4\,\epsilon\,c_4\,k_2\left(c_1\,k_1+c_3\,k_3\right)c_2+c_3\,c_4^{\,2}\,k_3\,k_4^{\,2}\right)}{c_2\left(c_1\,k_1+c_3\,k_3\right)c_4\left(\left(\left(-c_1-c_3\right)k_4+c_1\,k_1\right)k_2+c_3\,k_3\,k_4\right)(\epsilon+1)}$$

Substituting it back into the expressions for [A01] and [A10], we get the following correlations

$$simplify(A01) = \frac{-c_3\,c_4\,\epsilon\,k_3\,k_4-c_1\,c_2\,k_1\,k_2}{c_2\,c_4\left(\left(\left(-c_1-c_3\right)k_4+c_1\,k_1\right)k_2+c_3\,k_3\,k_4\right)(\epsilon+1)} \quad (3.1.2)$$

$$simplify(A10) = \frac{-c_1\,c_2\,\epsilon\,k_1\,k_2-c_3\,c_4\,k_3\,k_4}{c_2\,c_4\left(\left(\left(-c_1-c_3\right)k_4+c_1\,k_1\right)k_2+c_3\,k_3\,k_4\right)(\epsilon+1)} \quad (3.1.3)$$

From this we can see that the sum of concentrations of the partially modified substrates ([A01] and [A10]) are independent of ϵ and are dependent only on a few key kinetic parameters. This thus shows that at an asymmetric steady state, the sum of the concentrations of the partially modified substrates is fixed at a given value.

$simplify(A01 + A10)$

$$\frac{-c_1 c_2 k_1 k_2 - c_3 c_4 k_3 k_4}{c_2 c_4 \left(\left(\left(-c_1 - c_3\right) k_4 + c_1 k_1\right) k_2 + c_3 k_3 k_4\right)} \tag{3.1.4}$$

Necessary Conditions

Since substrate concentrations are always necessarily positive, the expression in equation 3.1.1 should be positive. The numerator is only a function of kinetic parameters all of which are positive always, thus the denominator must necessarily be positive to ensure that the resulting concentration is positive. This gives us the necessary condition for symmetric state to exist as follows.

1. $c_3 k_4 \left(k_2 - k_3\right) - c_1 k_2 \left(k_1 - k_4\right) > 0$

Sufficiency of necessary conditions

In this section of the proof we show the necessary conditions shown above are also sufficient for an asymmetric steady state to exist for some positive A_{Total} value. or upon a bifurcation along A_{Total} we are bound to encounter symmetry breaking provided the necessary conditions are satisfied. Note that in the context of this system, a feasible steady state is one in which the concentrations of all substrates, complexes and enzymes are positive.

We do this by showing that an asymmetric state defined by the invariant concentrations described above indeed is a solution for the system of ODEs at some positive A_{Total} value.

In an symmetric steady state, as seen above [Ap] is fixed by a few kinetic constants,

$$A01 := simplify\left(\frac{-c_3 c_4 \epsilon k_3 k_4 - c_1 c_2 k_1 k_2}{\left(\left(\left(-c_1 - c_3\right) k_4 + c_1 k_1\right) k_2 + c_3 k_3 k_4\right) c_2 c_4 (\epsilon + 1)}\right) =$$

$$\frac{-c_3 c_4 \epsilon k_3 k_4 - c_1 c_2 k_1 k_2}{c_2 c_4 \left(\left(\left(-c_1 - c_3\right) k_4 + c_1 k_1\right) k_2 + c_3 k_3 k_4\right) (\epsilon + 1)}$$

$$A10 := simplify\left(\frac{-c_1 c_2 \epsilon k_1 k_2 - c_3 c_4 k_3 k_4}{\left(\left(\left(-c_1 - c_3\right) k_4 + c_1 k_1\right) k_2 + c_3 k_3 k_4\right) c_2 c_4 (\epsilon + 1)}\right) =$$

$$\frac{-c_1 c_2 \epsilon k_1 k_2 - c_3 c_4 k_3 k_4}{c_2 c_4 \left(\left(\left(-c_1 - c_3\right) k_4 + c_1 k_1\right) k_2 + c_3 k_3 k_4\right) (\epsilon + 1)} =$$

The other variables in this asymmetric state are thus given by

$$simplify(A11) = -\frac{\epsilon \left(c_1 c_2^2 k_1 k_2^2 + k_4 \epsilon c_4 k_2 \left(c_1 k_1 + c_3 k_3\right) c_2 + c_3 c_4^2 k_3 k_4^2\right)}{c_2 \left(c_1 k_1 + c_3 k_3\right) c_4 \left(\left(\left(-c_1 - c_3\right) k_4 + c_1 k_1\right) k_2 + c_3 k_3 k_4\right) (\epsilon + 1)}$$

$$simplify(A00) = \frac{-c_1 c_2^2 \epsilon k_1 k_2^2 - c_2 c_4 k_4 \left(c_1 k_1 + c_3 k_3\right) k_2 - c_3 c_4^2 \epsilon k_3 k_4^2}{c_2 \left(c_1 k_1 + c_3 k_3\right) c_4 \left(\left(\left(-c_1 - c_3\right) k_4 + c_1 k_1\right) k_2 + c_3 k_3 k_4\right) (\epsilon + 1) \epsilon}$$

$$simplify\left(A00K_1\right) = -\frac{c_1 P \left(c_2 k_1 k_2 \left(c_2 \epsilon k_2 + c_4 k_4\right) c_1 + c_3 c_4 k_3 k_4 \left(c_4 \epsilon k_4 + c_2 k_2\right)\right)}{\left(c_1 k_1 + c_3 k_3\right) \left(k_2 \left(k_1 - k_4\right) c_1 - c_3 k_4 \left(k_2 - k_3\right)\right) c_2 (\epsilon + 1) c_4}$$

$$simplify\left(A00K_2\right) = -\frac{c_3 P \left(c_2 k_1 k_2 \left(c_2 \epsilon k_2 + c_4 k_4\right) c_1 + c_3 c_4 k_3 k_4 \left(c_4 \epsilon k_4 + c_2 k_2\right)\right)}{\left(c_1 k_1 + c_3 k_3\right) \left(k_2 \left(k_1 - k_4\right) c_1 - c_3 k_4 \left(k_2 - k_3\right)\right) c_2 (\epsilon + 1) c_4}$$

$$simplify(A01K) = -\frac{\epsilon P \left(c_3 c_4 \epsilon k_3 k_4 + c_2 k_1 k_2 c_1\right)}{\left(\left(\left(-c_1 - c_3\right) k_4 + c_1 k_1\right) k_2 + c_3 k_3 k_4\right) (\epsilon + 1) c_4}$$

$$simplify(A10K) = -\frac{\epsilon P \left(c_1 c_2 \epsilon k_1 k_2 + c_3 c_4 k_3 k_4\right)}{\left(\left(\left(-c_1 - c_3\right) k_4 + c_1 k_1\right) k_2 + c_3 k_3 k_4\right) c_2 (\epsilon + 1)}$$

$$simplify\left(A11P_1\right) = -\frac{c_3 P \epsilon \left(c_4 k_3 k_4 \left(c_2 \epsilon k_2 + c_4 k_4\right) c_3 + c_2 k_1 k_2 c_1 \left(c_4 \epsilon k_4 + c_2 k_2\right)\right)}{\left(c_1 k_1 + c_3 k_3\right) \left(k_2 \left(k_1 - k_4\right) c_1 - c_3 k_4 \left(k_2 - k_3\right)\right) c_2 (\epsilon + 1) c_4}$$

$$simplify\left(A11P_2\right) = -\frac{c_1 P \epsilon \left(c_4 k_3 k_4 \left(c_2 \epsilon k_2 + c_4 k_4\right) c_3 + c_2 k_1 k_2 c_1 \left(c_4 \epsilon k_4 + c_2 k_2\right)\right)}{\left(c_1 k_1 + c_3 k_3\right) \left(k_2 \left(k_1 - k_4\right) c_1 - c_3 k_4 \left(k_2 - k_3\right)\right) c_2 (\epsilon + 1) c_4}$$

$$simplify(A01P) = -\frac{P \left(c_3 c_4 \epsilon k_3 k_4 + c_2 k_1 k_2 c_1\right)}{\left(\left(\left(-c_1 - c_3\right) k_4 + c_1 k_1\right) k_2 + c_3 k_3 k_4\right) c_2 (\epsilon + 1)}$$

$$simplify(A10P) = -\frac{P \left(c_1 c_2 \epsilon k_1 k_2 + c_3 c_4 k_3 k_4\right)}{\left(\left(\left(-c_1 - c_3\right) k_4 + c_1 k_1\right) k_2 + c_3 k_3 k_4\right) (\epsilon + 1) c_4}$$

The system of ODE is also satisfied at this point, as is verified below.

$simplify(dA00) = 0$
$simplify(dA11) = 0$
$simplify(dA01) = 0$
$simplify(dA10) = 0$
$simplify\left(dA00K_1\right) = 0$
$simplify\left(dA00K_2\right) = 0$
$simplify(dA01P) = 0$
$simplify(dA10P) = 0$
$simplify(dA01K) = 0$
$simplify(dA10K) = 0$
$simplify\left(dA11P_1\right) = 0$

$simplify(dA11P_2) = 0$
$simplify(dP) = 0$
$simplify(dK) = 0$

Hence all that remains to be shown is that the variables (As described above) are positive for some value of A_{Total}.
This is true if and only if

1. **Necessary condition is satisfied**
2. **P and ϵ are positive.**

Now by using the total conservation of phosphatase in the system (PCon = 0), we obtain an algebraic expression for [P] in terms of P_{Total}, kinetic constants and ϵ as shown below. The Maple command *solve* is used for this purpose as shown below.

$$P := simplify(solve(PCon, P)) =$$
$$-\left(c_2 (c_1 k_1 + c_3 k_3) c_4 (k_2 (k_1 - k_4) c_1 - c_3 k_4 (k_2 - k_3)) P_{Total} (\epsilon + 1)\right) \Big/ \left(c_2 c_4 k_2 k_4 (c_1 + c_3) (c_1 k_1 + c_3 k_3) \epsilon^2 + \left(c_2 k_2 ((k_1 + k_2) c_2 - c_4 (k_1 - k_4)) k_1 c_1^2 + \left(k_1 k_2 (k_2 + k_3) c_2^2 - c_4 (((-k_1 - k_3) k_4 + k_1 k_3) k_2 + k_1 k_3 k_4) c_2 + c_4^2 k_3 k_4 (k_1 + k_4)\right) c_3 c_1 + c_4 ((k_2 - k_3) c_2 + c_4 (k_3 + k_4)) k_4 c_3^2 k_3\right) \epsilon + c_2 c_4 k_2 k_4 (c_1 + c_3) (c_1 k_1 + c_3 k_3)\right)$$

Hence if ϵ is positive P is automatically positive (provided necessary conditions are satisfied) . Hence we have shown that the individual conservation equations for the enzymes (KCon and PCon) and the system of ODEs is solved by the expressions above, and represent feasible solutions provided ϵ is positive.

Since the concentrations are all positive there exists a unique A_{Total} value for every ϵ (permitting asymmetric states).

Hence we have proved that symmetry breaking is guaranteed for some finite positive A_{Total}, provided the necessary conditions above are satisfied - making those conditions sufficient for the behavior.

Prediction of pitchfork bifurcation along A_{Total}

Here we predict the value of A_{Total} at which symmetry breaking occurs via a pitch fork bifurcation. This point in the bifurcation is characterized by the intersection of both the symmetric steady state branch and the asymmetric steady state branches.

Hence at the position of symmetry breaking, we know two insights.

1. The system is still symmetric.

2. The invariant describing the asymmetric steady state is also true.

Using these two information, we can simplify the original system considerably as follows.

$\epsilon := 1 :$

$$A11 := simplify\left(eval\left(-\frac{\left(c_1\, c_2^{\,2}\, k_1\, k_2^{\,2} + k_4\, \epsilon\, c_4\, k_2\, (c_1\, k_1 + c_3\, k_3)\, c_2 + c_3\, c_4^{\,2}\, k_3\, k_4^{\,2}\right)\epsilon}{(c_1\, k_1 + c_3\, k_3)\,\left(\left((-c_1 - c_3)\, k_4 + c_1\, k_1\right) k_2 + c_3\, k_3\, k_4\right)(\epsilon + 1)\, c_4\, c_2}, \epsilon = 1\right)\right)$$

$$A11 := -\frac{(c_2\, k_2 + k_4\, c_4)\,(c_1\, c_2\, k_1\, k_2 + c_3\, c_4\, k_3\, k_4)}{2\, c_2\, (c_1\, k_1 + c_3\, k_3)\, c_4\, \left(k_2\, (k_1 - k_4)\, c_1 - c_3\, k_4\, (k_2 - k_3)\right)} \qquad \textbf{(3.4.1)}$$

Now, by solving the conservation expression for the substrate we can isolate the value of A_{Total} when the asymmetric steady states and the symmetric steady state intersect (indicating the pitchfork bifurcation point).

$A_{Total} = simplify(solve(ACon, A_{Total}))$

$$A_{Total} = \left(2\,(c_1\, c_2\, k_1\, k_2 + c_3\, c_4\, k_3\, k_4)\left(-\frac{k_2^{\,2}\left((k_1 + k_2)\, c_1 + c_3\, (k_2 + k_3)\right) c_1\, k_1\, c_2^{\,3}}{2} + \left(\left(k_1\, k_2\, P_{Total}\, (k_1 - k_4)\, (k_1 + k_2)\, c_1^{\,3} + \left(\left(\left((-2\, k_1 - k_3)\, k_4 + k_1\, (k_1 + k_3)\right) k_2^{\,2} + \left(-k_1\, (k_1 + k_3)\, k_4 + 2\, k_1^{\,2}\, k_3\right) k_2 + k_1^{\,2}\, k_3\, k_4\right) P_{Total}\, c_3 + \frac{k_2\, k_1\, \left((k_1 - 4\, k_4)\, k_2 - k_4\, k_1\right)}{2}\right) c_1^{\,2} + \left(\left(\left((-k_1 - 2\, k_3)\, k_4 + k_1\, k_3\right) k_2^{\,2} + k_1\, k_3\, (k_3 - k_4)\, k_2 + 2\, k_1\, k_3^{\,2}\, k_4\right) P_{Total}\, c_3 + \frac{k_2^{\,2}\left((-4\, k_1 - 3\, k_3)\, k_4 + k_1\, k_3\right)}{2}\right) c_3\, c_1 - \left(\left(k_2^{\,2}\, P_{Total} - k_3^{\,2}\, P_{Total}\right) c_3 + \frac{3\left(k_2 - \frac{k_3}{3}\right) k_2}{2}\right) k_4\, c_3^{\,2}\, k_3\right) c_4 \qquad \textbf{(3.4.2)}$$

$$
- \frac{(c_1 k_1 + c_3 k_3) k_2 ((k_1 + k_2) c_1 + c_3 (k_2 + k_3)) c_1 k_1}{2} \Bigg) c_2^2
$$

$$
+ c_4 \Bigg(\Bigg(\Bigg(k_1 k_2 P_{Total} (k_1 - k_4) (k_1 + k_4) c_1^3 + \Bigg(2 \Bigg(\Bigg(\Bigg(\Bigg(-k_1 - \frac{k_3}{2} \Bigg) k_4^2 - \frac{k_1 k_3 k_4}{2}
$$

$$
+ k_1^2 k_3 \Bigg) k_2 + \frac{k_1 k_3 k_4 (k_1 + k_4)}{2} \Bigg) P_{Total} c_3 + \frac{k_1 k_2 k_4 (k_1 - 3 k_4)}{2} \Bigg) c_1^2
$$

$$
+ \Bigg(\Big(\big((-k_1 - 2 k_3) k_4^2 - k_3 (k_1 + k_3) k_4 + k_1 k_3^2 \big) k_2 + k_3 (k_1 + k_3) k_4^2
$$

$$
+ 2 k_1 k_3^2 k_4 \Big) P_{Total} c_3 - \frac{3 k_4^2 \left(\left(k_1 + \frac{4 k_3}{3} \right) k_2 - \frac{k_1 k_3}{3} \right)}{2} \Bigg) c_3 c_1
$$

$$
- \Bigg(P_{Total} (k_3 + k_4) (k_2 - k_3) c_3 + \left(\left(\frac{k_3}{2} + 2 k_4 \right) k_2 - \frac{k_4 k_3}{2} \right) k_4 c_3^2 k_3 \Bigg) c_4
$$

$$
+ \frac{(c_1 k_1 + c_3 k_3)^2 \left(k_2 (k_1 - 3 k_4) c_1 - 3 \left(k_2 - \frac{k_3}{3} \right) k_4 c_3 \right)}{2} \Bigg) c_2
$$

$$
- \frac{c_4^2 (c_1 k_1 + c_3 k_3 + k_4 c_4) k_4 ((k_1 + k_4) c_1 + c_3 (k_3 + k_4)) c_3 k_3}{2} \Bigg) \Bigg) \Bigg/
$$

$$
\Bigg(c_2 (c_1 k_1 + c_3 k_3) c_4 \Big(k_2 ((k_1 + k_2) c_1 + c_3 (k_2 + k_3)) c_1 k_1 c_2^2 - (c_1 k_1
$$

$$
+ c_3 k_3) c_4 \left(k_2 (k_1 - 3 k_4) c_1 - 3 \left(k_2 - \frac{k_3}{3} \right) k_4 c_3 \right) c_2 + c_4^2 k_4 ((k_1 + k_4) c_1
$$

$$
+ c_3 (k_3 + k_4)) c_3 k_3 \Big) (k_2 (k_1 - k_4) c_1 - c_3 k_4 (k_2 - k_3)) \Bigg)
$$

A cross verification of this analytical work is carried out in the read me file for the parameters used in generating the figure (Fig 3A).

Random distributive DSP System 2 : Separate Kinase Common Phosphatase

Case 1	Case 2	Case 3
Symmetry Doesn't Exist	Present and Can't Break	Symmetry Doesn't Exist

In this Maple file we analytically show the infeasibility of case 2 symmetry breaking in Random distributive DSP network with separate kinase and common phosphatase (System 2) effecting phosphorylation and dephoshorylation respectively.We do this by first describing the model as a system of ODEs along with the associated enzyme and substrate conservations. We then impose the kinetic constraints (and constraints on total enzyme concentrations) pertaining to case 2 symmetry. By algebraically solving for the steady state of the resulting system of ODEs we obtain relations between concentrations of the substrate variables in terms of each other and the free enzyme concentrations. After this, we identify key symmetric pairings that represent the symmetric steady state. i.e. case 2 symmetry breaking requires symmetry between [A01] & [A10]; By leveraging this insight, we show the infeasibility of case 2 symmetry breaking by revealing that [A01] and [A10] are always equal for any given feasible steady state. These procedures are carried out in detail below using built in Maple commands.

Note: A subscript is used to distinguish between the two different complexes formed between [P] and [A11] ([$A11P_1$ and $A11P_2$]).

We initialize the Maple file with the restart command and load the relevant libraries of inbuilt Maple functions (*LinearAlgebra, VectorCalculus, Student[LinearAlgebra]*)

restart : *with*(*LinearAlgebra*) : *with*(*VectorCalculus*) : *with*(*Student*[*LinearAlgebra*]) :

The system is modelled as a set of ODEs using the kinetic nomenclature described in the main text and supplementary figure (refer Appendix 2 figure 10). Here dA00 represents d[A00]/dt and similarly for other expressions. At steady state thus, each of the right hand sides of these expressions will be equal to zero.

$dA00 := k_4 \cdot A01P + a_4 \cdot A10P + k_{ub1} \cdot A00K1 + a_{ub1} \cdot A00K2 - k_{b1} \cdot A00 \cdot K1 - a_{b1} \cdot A00 \cdot K2 :$

$dA01 := k_1 \cdot A00K1 + k_3 \cdot A11P_1 + k_{ub2} \cdot A01K2 + k_{ub4} \cdot A01P - k_{b2} \cdot A01 \cdot K2 - k_{b4} \cdot A01 \cdot P :$

$dA10 := a_1 \cdot A00K2 + a_3 \cdot A11P_2 + a_{ub2} \cdot A10K1 + a_{ub4} \cdot A10P - a_{b2} \cdot A10 \cdot K1 - a_{b4} \cdot A10 \cdot P :$

$dA11 := k_2 \cdot A01K2 + a_2 \cdot A10K1 + k_{ub3} \cdot A11P_1 + a_{ub3} \cdot A11P_2 - k_{b3} \cdot A11 \cdot P - a_{b3} \cdot A11 \cdot P :$

$dA00K1 := k_{b1} \cdot A00 \cdot K1 - (k_1 + k_{ub1}) \cdot A00K1 :$

$dA10K1 := a_{b2} \cdot A10 \cdot K1 - (a_2 + a_{ub2}) \cdot A10K1 :$

$dA00K2 := a_{b1} \cdot A00 \cdot K2 - (a_{ub1} + a_1) \cdot A00K2 :$

$dA01K2 := k_{b2} \cdot A01 \cdot K2 - (k_{ub2} + k_2) \cdot A01K2 :$

$dA11P_1 := k_{b3} \cdot A11 \cdot P - k_{ub3} \cdot (A11P_1) - k_3 \cdot (A11P_1) :$

$dA10P := a_{b4} \cdot A10 \cdot P - (a_{ub4} + a_4) \cdot A10P :$

$dA11P_2 := a_{b3} \cdot A11 \cdot P - (a_{ub3} + a_3) \cdot A11P_2 :$

$dA01P := k_{b4} \cdot A01 \cdot P - (k_{ub4} + k_4) \cdot A01P :$

$dK1 := -k_{b1} \cdot A00 \cdot K1 + (k_1 + k_{ub1}) \cdot A00K1 - a_{b2} \cdot A10 \cdot K1 + (a_2 + a_{ub2}) \cdot A10K1 :$
$dK2 := -a_{b1} \cdot A00 \cdot K2 + (a_{ub1} + a_1) \cdot A00K2 - k_{b2} \cdot A01 \cdot K2 + (k_{ub2} + k_2) \cdot A01K2 :$
$dP := -k_{b3} \cdot A11 \cdot P + (k_{ub3} + k_3) \cdot A11P_1 - a_{b4} \cdot A10 \cdot P + (a_{ub4} + a_4) \cdot A10P - a_{b3} \cdot A11 \cdot P + (a_{ub3} + a_3) \cdot A11P_2 - k_{b4} \cdot A01 \cdot P + (k_{ub4} + k_4) \cdot A01P :$

The above equations are also associated with conservation conditions which are described below. Here we store the conservation expressions as ACon, PCon, K1Con and K2Con for the substrate and the respective enzymes. Each of these expressions is always equal to zero (both in the transient behavior and at steady state).

$ACon := A_{Total} - A00 - A10 - A01 - A11 - A00K1 - A01K2 - A00K2 - A10K1 - A11P_1 - A10P - A11P_2 - A01P :$
$K1Con := K1_{Total} - K1 - A00K1 - A10K1 :$
$K2Con := K2_{Total} - K2 - A00K2 - A01K2 :$
$PCon := P_{Total} - P - A11P_1 - A10P - A11P_2 - A01P :$

Case 2 Symmetry : Present and Can't Break

Kinetic constraints for case 2 symmetry (refer main text) are imposed on the original model.

$a_1 := k_1 : a_{b1} := k_{b1} : a_{ub1} := k_{ub1} :$
$a_2 := k_2 : a_{b2} := k_{b2} : a_{ub2} := k_{ub2} :$
$a_3 := k_3 : a_{b3} := k_{b3} : a_{ub3} := k_{ub3} :$
$a_4 := k_4 : a_{b4} := k_{b4} : a_{ub4} := k_{ub4} :$

In addition to the kinetic constraints the total enzyme concentrations of the two kinases need to be equal for exact case 2 symmetry to be present. This is imposed as shown below.

$K1_{Total} := K_{Total} :$
$K2_{Total} := K_{Total} :$

At this stage we introduce auxiliary constants c_1, c_2, c_3 and c_4 in place of the binding constants so as to make further analytical expressions more accessible.

$k_{b1} := c_1 \cdot (k_1 + k_{ub1}) :$
$k_{b2} := c_2 \cdot (k_2 + k_{ub2}) :$
$k_{b3} := c_3 \cdot (k_3 + k_{ub3}) :$
$k_{b4} := c_4 \cdot (k_4 + k_{ub4}) :$

Once this is done, we solve for the steady state of the system in terms of fewer key variables. In this context we want to solve all variables in terms of the concentrations of the free enzymes ([K1], [K2] & [P]) and concentration of the completely unmodified substrate ([A00]). In order to

do this, we use the Maple command *solve,* which algebraically solves the equation supplied for a given variable. We first solve for the individual complexes using their corresponding differential equation. An example of this (using [A00K1]) is given below in detail.

The differential equation of [A00K1] is given by,

$$\frac{d\,[A00K1]}{dt} = dA00K1$$

$$\frac{d\,[A00K1]}{dt} = c_1\,(k_1 + k_{ub1})\,A00\,K1 - (k_1 + k_{ub1})\,A00K1 \tag{1.1}$$

The solve command by Maple, uses this solves this equation for the given variable (in this case [A00K1]). This is performed by the following command. Here we assign [A00K1], the solution returned by the *solve* command.

$$A00K1 := solve(dA00K1, A00K1)$$

$$A00K1 := K1\,A00\,c_1 \tag{1.2}$$

This operation is performed for the other complexes and substrate forms as well. Here we simultaneously solve expressions for the substrate forms using the *solve* command as shown below. The solution is stored in a variable labelled *Sol*, and then the respective solutions are extracted from this vector using the *eval* command.

$A01K2 := solve(dA01K2, A01K2)$:
$A00K2 := solve(dA00K2, A00K2)$:
$A10K1 := solve(dA10K1, A10K1)$:
$A11P_1 := solve(dA11P_1, A11P_1)$:
$A10P := solve(dA10P, A10P)$:
$A11P_2 := solve(dA11P_2, A11P_2)$:
$A01P := solve(dA01P, A01P)$:

$Sol := solve(\{dA10, dA01, dA11\}, \{A10, A01, A11\})$:

$A10 := eval(A10, Sol)$:
$A01 := eval(A01, Sol)$:
$A11 := eval(A11, Sol)$:

Doing this results in the following correlations between the concentrations of the various substrate forms at steady state.

$$A00 = A00$$

$$A01 = \frac{K1\,A00\,c_1\,k_1}{c_4\,k_4\,P}$$

$$A10 = \frac{K2\,A00\,c_1\,k_1}{c_4\,k_4\,P}$$

$$A11 = \frac{A00\ K1\ K2\ c_1\ c_2\ k_1\ k_2}{c_4\ k_4\ P^2\ c_3\ k_3}$$

Proof for impossibility of symmetry breaking

We know that K1Con and K2Con are both individually equal to zero always. Thus at a given steady state, K1Con - K2Con must also be equal to zero.

We thus introduce the term T = (K1Con - K2Con) = 0 and also introduce a new ratio, ϵ = [K2]/[K1]. Note: As discussed in the main text, the symmetric steady state is one where [K1] = [K2] or ϵ = 1. Since we are isolating solutions of asymmetry, we are primarily interested in solutions that permit, $\epsilon \neq 1$.

$T := K1Con - K2Con = 0 :$
$K2 := \epsilon \cdot K1 :$

The following command (*simplify*), simplifies the expression algebraically

$simplify(T) = K1\ (\epsilon - 1)\ (A00\ c_1 + 1) = 0$

Thus from this we can clearly see that irrespective of kinetic parameters, [K1] is always going to be equal to [K2]. This means, from the expressions given above for [A01] and [A10], that [A01] is always equal to [A10] irrespective of kinetic parameters or total concentrations of enzymes or substrate. Thus there is no scope for any asymmetric steady state or case 2 symmetry breaking.

Random DSP System 3 : Separate Kinase Separate Phosphatase

Case 1	Case 2	Case 3
**Present and Breaks Invariant identified**	**Present and Breaks Invariant identified**	**Present and breaks**

In this maple file we analytically show presence of case 1 and case 2 symmetry breaking in Random DSP network with separate kinase and separate phosphatase (System 3) effecting phosphorylation and dephoshorylation respectively. In each case, we do this by first describing the model as a system of ODEs along with the associated enzyme and substrate conservations. By algebraically solving for the steady state of the system of ODEs we obtain relations between substrate variables (concentrations) in terms of each other and the free enzyme concentrations. After this, we identify key symmetric pairings that represent the symmetric steady state. i.e. In Random System 3, case 1 symmetry symmetry requires symmetry between [A00] & [A11]; case 2 symmetry breaking requires symmetry between [A01] & [A10].

We then proceed along two lines. One hand by leveraging this insight, we isolate correlations pertaining to asymmetric steady states involving substrate forms and free enzymes. Through an alternate approach considering the conservation of individual species and an overall flux balance around substrate forms, we ascertain a secondary correlation that is pertinent to all feasible steady states of the system. By bringing the two conditions together, we then finally ascertain the necessary conditions and features of symmetry breaking in case 1 and case 2 symmetry. These procedures are carried out in detail below using built in maple commands.

We initialize the maple file with the *restart* command and load the relevant libraries of inbuilt maple functions (*LinearAlgebra, VectorCalculus, Student[LinearAlgebra]*)

$restart : with(LinearAlgebra) : with(VectorCalculus) : with(Student[LinearAlgebra]) :$

The system is modelled as a set of ODEs using the kinetic nomenclature described in the main text and supplementary figure (refer Appendix 2 figure 10). Here dA00 represents d[A00]/dt and similarly for other expressions. At steady state thus, each of the right hand sides of these expressions will be equal to zero.

$$dA00 := k_4 \cdot A01P1 + a_4 \cdot A10P2 + k_{ub1} \cdot A00K1 + a_{ub1} \cdot A00K2 - k_{b1} \cdot A00 \cdot K1 - a_{b1} \cdot A00 \cdot K2 :$$
$$dA01 := k_1 \cdot A00K1 + k_3 \cdot A11P2 + k_{ub2} \cdot A01K2 + k_{ub4} \cdot A01P1 - k_{b4} \cdot A01 \cdot P1 - k_{b2} \cdot A01 \cdot K2 :$$
$$dA10 := a_1 \cdot A00K2 + a_3 \cdot A11P1 + a_{ub2} \cdot A10K1 + a_{ub4} \cdot A10P2 - a_{b2} \cdot A10 \cdot K1 - a_{b4} \cdot A10 \cdot P2 :$$
$$dA11 := k_2 \cdot A01K2 + a_2 \cdot A10K1 + k_{ub3} \cdot A11P2 + a_{ub3} \cdot A11P1 - k_{b3} \cdot A11 \cdot P2 - a_{b3} \cdot A11 \cdot P1 :$$
$$dA00K1 := k_{b1} \cdot A00 \cdot K1 - (k_1 + k_{ub1}) \cdot A00K1 :$$
$$dA01K2 := k_{b2} \cdot A01 \cdot K2 - (k_2 + k_{ub2}) \cdot A01K2 :$$
$$dA11P2 := k_{b3} \cdot A11 \cdot P2 - (k_3 + k_{ub3}) \cdot A11P2 :$$
$$dA01P1 := k_{b4} \cdot A01 \cdot P1 - (k_4 + k_{ub4}) \cdot A01P1 :$$
$$dA00K2 := a_{b1} \cdot A00 \cdot K2 - (a_1 + a_{ub1}) \cdot A00K2 :$$
$$dA10K1 := a_{b2} \cdot A10 \cdot K1 - (a_2 + a_{ub2}) \cdot A10K1 :$$
$$dA11P1 := a_{b3} \cdot A11 \cdot P1 - (a_3 + a_{ub3}) \cdot A11P1 :$$

$dA10P2 := a_{b4} \cdot A10 \cdot P2 - (a_4 + a_{ub4}) \cdot A10P2 :$
$dP2 := -k_{b3} \cdot A11 \cdot P2 + (k_3 + k_{ub3}) \cdot A11P2 - a_{b4} \cdot A10 \cdot P2 + (a_4 + a_{ub4}) \cdot A10P2 :$
$dP1 := -a_{b3} \cdot A11 \cdot P1 + (a_3 + a_{ub3}) \cdot A11P1 - k_{b4} \cdot A01 \cdot P1 + (k_4 + k_{ub4}) \cdot A01P1 :$
$dK1 := -k_{b1} \cdot A00 \cdot K1 + (k_1 + k_{ub1}) \cdot A00K1 - a_{b2} \cdot A10 \cdot K1 + (a_2 + a_{ub2}) \cdot A10K1 :$
$dK2 := -a_{b1} \cdot A00 \cdot K2 + (a_1 + a_{ub1}) \cdot A00K2 - k_{b2} \cdot A01 \cdot K2 + (k_2 + k_{ub2}) \cdot A01K2 :$

The above equations are also associated with conservation conditions which are described below. Here we store the conservation expressions as ACon, K1Con, K2Con, P1Con and P2Con for the substrate and the respective enzymes. Each of these expressions is always equal to zero (both in the transient behavior and at steady state).

$ACon := A_{Total} - A00 - A10 - A01 - A11 - A00K1 - A01K2 - A11P2 - A01P1 - A00K2 - A10K1 - A11P1 - A10P2 :$
$K1Con := K1_{Total} - A00K1 - A10K1 - K1 :$
$K2Con := K2_{Total} - A01K2 - A00K2 - K2 :$
$P1Con := P1_{Total} - A11P1 - A01P1 - P1 :$
$P2Con := P2_{Total} - A10P2 - A11P2 - P2 :$

Until now we have modelled the System 3 Random DSP network with separate kinase effecting phosphorylation and a separate phosphatase for dephosphorylation - without any impositions on kinetics or concentrations for symmetry. In the following segments, we specifically do this for each class of symmetry. The codes for each symmetry are modular and in order to run a particular symmetry, please run the code until this point and then run only the code for the specific class of symmetry.

Note: Do not run the whole script at the same time, as this will impose all symmetries at the same time and give incorrect results.

Case 1 Symmetry : Present and Breaks

Kinetic constraints for case 1 symmetry (refer main text) are imposed on the original model.

$k_3 := k_1 : k_{b3} := k_{b1} : k_{ub3} := k_{ub1} :$
$k_4 := k_2 : k_{b4} := k_{b2} : k_{ub4} := k_{ub2} :$

$a_3 := a_1 : a_{b3} := a_{b1} : a_{ub3} := a_{ub1} :$
$a_4 := a_2 : a_{b4} := a_{b2} : a_{ub4} := a_{ub2} :$

In addition to the kinetic constraints the total enzyme concentrations of corresponding kinases and phosphatases need to be equal for exact case 1 symmetry in Random DSP System 3. This is imposed as shown below.

$K1_{Total} := P2_{Total} :$

$K2_{Total} := P1_{Total}:$

At this stage we introduce auxiliary constants c_1, c_2, d_1 and d_2 in place of the binding constants so as to make further analytical expressions more accessible.

$k_{b1} := c_1 \cdot (k_1 + k_{ub1}):$
$k_{b2} := c_2 \cdot (k_2 + k_{ub2}):$

$a_{b1} := d_1 \cdot (a_1 + a_{ub1}):$
$a_{b2} := d_2 \cdot (a_2 + a_{ub2}):$

Once this is done, we solve for the steady state of the system in terms of fewer key variables. In this context we want to solve all variables in terms of the concentrations of the free enzymes ([K1], [K2], [P1] & [P2]) and concentration of the partially modified substrate [A01]. In order to do this, we use the Maple command *solve,* which solves the equation supplied for a given variable. We first solve for the individual complexes using their corresponding differential equation. An example of this (using [A00K1]) is given below in detail.

The differential equation of [A00K1] is given by,

$$\frac{d\,[A00K1]}{dt} = dA00K1 \qquad \frac{d\,[A00K1]}{dt} = c_1\,(k_1 + k_{ub1})\,A00\,K1 - (k_1 + k_{ub1})\,A00K1$$

The *solve* command by Maple, solves this equation for the given variable (in this case [A00K1]). We in turn store this value (the solution returned by the *solve* command) in [A00K1]. This is performed by the following command.

$A00K1 := solve(dA00K1, A00K1)$

$$A00K1 := K1\,A00\,c_1 \qquad \textbf{(1.1)}$$

This operation is performed for the other complexes and substrate forms as well. Here we simultaneously solve expressions for the substrate forms using the *solve* command as shown below. The solution is stored in a variable labelled *Sol*, and then the respective solutions are extracted from this vector using the *eval* command.

$A01K2 := solve(dA01K2, A01K2):$
$A11P2 := solve(dA11P2, A11P2):$
$A01P1 := solve(dA01P1, A01P1):$
$A00K2 := solve(dA00K2, A00K2):$
$A10K1 := solve(dA10K1, A10K1):$
$A11P1 := solve(dA11P1, A11P1):$
$A10P2 := solve(dA10P2, A10P2):$

$Sol := solve(\{dA00, dA11, dA10\}, \{A00, A11, A10\}):$

$A00 := eval(A00, Sol):$

$A01 := eval(A01, Sol) :$
$A10 := eval(A10, Sol) :$
$A11 := eval(A11, Sol) :$

Doing this results in the following correlations between the concentrations of the various substrate forms at steady state.

$$A00 = \frac{A01\ P1\ c_2\ k_2}{c_1\ k_1\ K1}$$

$$A01 = A01$$

$$A10 = \frac{A01\ K2\ P1\ a_1\ c_2\ d_1\ k_2}{P2\ c_1\ k_1\ K1\ a_2\ d_2}$$

$$A11 = \frac{K2\ A01\ c_2\ k_2}{P2\ c_1\ k_1}$$

Proof for invariant in asymmetric branches

We know that $(P1_{Total} \cdot P2_{Total} - K1_{Total} \cdot K2_{Total} = 0)$ in this system under case 1 symmetry (Since $K1_{Total}$ = $P2_{Total}$ and $K2_{Total}$ = $P1_{Total}$). Thus at a given steady state, $(P1_{Total} \cdot P2_{Total} - K1_{Total} \cdot K2_{Total})$ must be equal to 0. We introduce a term T = $(P1_{Total} \cdot P2_{Total} - K1_{Total} \cdot K2_{Total})$ as shown below. Here we write $P1_{Total}$ as $P1_{Total}$ - P1Con. Note: This comes from the expression used for P1Con earlier (P1Con = $P1_{Total}$ - P1 - A11P1 - A01P1) and the fact that PCon = 0. This way we have represented the independent enzyme concentrations as a sum of the free enzyme and the complexes in which it is sequestered.

Similarly for the other total enzyme concentrations as well.

$$T := (P1_{Total} - P1Con) \cdot (P2_{Total} - P2Con) - (K2_{Total} - K2Con) \cdot (K1_{Total} - K1Con) = 0 :$$

At this stage we evaluate the ratio of [A00] and [A11]. Note that with case 1 symmetry breaking, we are looking for asymmetric states such that [A00] not equal to [A11].

$$simplify\left(\frac{A00}{A11}\right) = \frac{P1\ P2}{K1\ K2}$$

We denote this ratio as ϵ. i.e. ϵ = (P2*P1)/(K2*K1).

$$P2 := \frac{\epsilon \cdot K1 \cdot K2}{P1} :$$

$$simplify\left(\frac{A00}{A11}\right) = \epsilon$$

After introducing this ratio and algebraically simplifying T using the Maple command *simplify*, we get the following equation.

$$simplify(T) = \frac{1}{K1\, c_1\, \epsilon\, k_1^2\, a_2}\Big((\epsilon - 1)\,\big(K1^2\, \epsilon\, a_2\, c_1\, (A01\, c_2 + 1)\, k_1^2 + k_2\, P1^2\, A01\, a_1\, c_2\, d_1\, (A01\, c_2 + 1)\, k_1 - A01^2\, P1^2\, a_2\, c_2^2\, d_1\, k_2^2\big)\, K2\Big) = 0$$

From this we can ascertain that, should an asymmetric steady state exist (where $\epsilon \neq 1$) - the term
$\big(K1^2\, a_2\, \epsilon\, c_1\, (A01\, c_2 + 1)\, k_1^2 + a_1\, P1^2\, A01\, c_2\, d_1\, k_2\, (A01\, c_2 + 1)\, k_1 - A01^2\, P1^2\, c_2^2\, d_1\, k_2^2\, a_2\big)$ **in the numerator of the expression needs to be zero. This term is an expression in the partial substrate form [A01], ϵ, the free enzymes and kinetic constants.**

We show here that this term is nothing but the numerator of the following expression where [A01] written as a function of [A10].

$$r := \left(\left(\frac{A01}{k_1}\cdot\frac{A10}{a_1} - \left(\frac{A01}{k_2} + \frac{1}{c_2 \cdot k_2}\right)\cdot\left(\frac{A10}{a_2} + \frac{1}{d_2 \cdot a_2}\right)\right)\right):$$

This is verified below. The *numer* command in Maple isolates only the numerator of an expression. In this context we isolate the numerator of 'r' and add the term denoting asymmetry from earlier. We can see that all expressions in the term cancel out revealing that they are identical.

$$simplify\Big(numer(r) + \big(K1^2\, a_2\, \epsilon\, c_1\, (A01\, c_2 + 1)\, k_1^2 + a_1\, P1^2\, A01\, c_2\, d_1\, k_2\, (A01\, c_2 + 1)\, k_1 - A01^2\, P1^2\, c_2^2\, d_1\, k_2^2\, a_2\big)\Big)$$
$$= 0$$

Thus, here we have a clear representation of the asymmetric steady state in terms of a correlation between only the concentrations of the partially modified enzymes and key kinetic constants (i.e. r = 0). r in turn can be rewritten for simplicity as follows.

$restart:$

$r := \lambda_{A01} \cdot \lambda_{A10} - 1:$

Where the λ_{A01} and λ_{A10} expressions are given as follows.

$$\lambda_{A01} := simplify\left(\frac{\frac{A01}{k_1}}{\left(\frac{A01}{k_2}+\frac{1}{c_2 \cdot k_2}\right)}\right) = \frac{A01\, c_2\, k_2}{k_1\, (A01\, c_2 + 1)}$$

$$\lambda_{A10} := simplify\left(\frac{\frac{A10}{a_1}}{\left(\frac{A10}{a_2}+\frac{1}{d_2 \cdot a_2}\right)}\right) = \frac{A10\, d_2\, a_2}{a_1\, (A10\, d_2 + 1)}$$

Now we use an auxiliary analysis to ascertain other correlations between [A01] and [A10] representing all steady states of the system. We refresh Maple here to rewrite some new expressions.

restart :

We begin by evaluating the following ratio of enzymes $\frac{(P2_{Total} + K1_{Total})}{(P1_{Total} + K2_{Total})}$**. We know from the individual conservation equations that they correspond to the sum of the free enzyme and the complexes in which they are sequestered. Thus,**

$$\frac{(P2_{Total} + K1_{Total})}{(P1_{Total} + K2_{Total})} = \frac{(A10P2 + A11P2 + P2) + (A00K1 + A10K1 + K1)}{(A11P1 + A01P1 + P1) + (A01K2 + A00K2 + K2)} :$$

This can be further simplified as follows, (by rewriting the concentration of the complexes at steady state and simplifying the resulting expression)

$$\frac{(P2_{Total} + K1_{Total})}{(P1_{Total} + K2_{Total})} = \frac{(A11P2 + A00K1) + (1 + d_2 \cdot A10) \cdot (K1 + P2)}{(A00K2 + A11P1) + (1 + c_2 \cdot A01) \cdot (K2 + P1)}$$

Now from the dynamical representation of the network as a system of ODEs earlier (particularly equations dA01 and dA10) we know that at steady state,

$$A11P2 + A00K1 = \frac{k_2}{k_1} \cdot (A01K2 + A01P1) :$$

$$A11P1 + A00K2 = \frac{a_2}{a_1} \cdot (A10P2 + A10K1) :$$

Substituiting this in the earlier expression we get the following,

$$\frac{\left(P2_{Total} + K1_{Total}\right)}{\left(P1_{Total} + K2_{Total}\right)} = \frac{\frac{k_2}{k_1}\cdot(A01K2 + A01P1) + \left(1 + d_2\cdot A10\right)\cdot(K1 + P2)}{\frac{a_2}{a_1}\cdot(A10P2 + A10K1) + \left(1 + c_2\cdot A01\right)\cdot(K2 + P1)} :$$

Which simplifies to,

$$\frac{\left(P2_{Total} + K1_{Total}\right)}{\left(P1_{Total} + K2_{Total}\right)} = \frac{\frac{k_2}{k_1}\cdot c_2\cdot A01\cdot(K2 + P1) + \left(1 + d_2\cdot A10\right)\cdot(K1 + P2)}{\frac{a_2}{a_1}\cdot d_2\cdot A10\cdot(P2 + K1) + \left(1 + c_2\cdot A01\right)\cdot(K2 + P1)} :$$

Now we introduce a new ratio, $\alpha = \frac{\left(1 + c_2\cdot A01\right)\cdot(K2 + P1)}{\left(1 + d_2\cdot A10\right)\cdot(K1 + P2)}$ **Substituting this ratio in the above expression we get the following simplified form,**

$$\frac{\left(P2_{Total} + K1_{Total}\right)}{\left(P1_{Total} + K2_{Total}\right)} = \frac{\frac{k_2}{k_1}\cdot\frac{c_2\cdot A01\cdot \text{alpha}}{\left(1 + c_2\cdot A01\right)} + 1}{\frac{a_2}{a_1}\cdot\frac{d_2\cdot A10}{\left(1 + d_2\cdot A10\right)} + \alpha} :$$

Reintroducting the nomenclature of λ_{A01} **and** λ_{A10} **the above equation simplifies as,**

$$\frac{\left(P2_{Total} + K1_{Total}\right)}{\left(P1_{Total} + K2_{Total}\right)} = \frac{\lambda_{A01}\cdot\alpha + 1}{\lambda_{A10} + \alpha} :$$

Under case 1 symmetry, $K2_{Total} = P1_{Total}$**, and** $K1_{Total} = P2_{Total}$**. Here we bring in the asymmetry correlation ascertained earlier, i.e. r = 1 or** $\lambda_{A01} = \frac{1}{\lambda_{A10}}$ **. Using these two insights, the above equation simplifies to**

$$\frac{P2_{Total}}{P1_{Total}} = \lambda_{A01}\cdot\frac{\left(\lambda_{A01}\cdot\alpha + 1\right)}{\left(1 + \alpha\cdot\lambda_{A01}\right)}$$

Since the expression on the right is always positive (α **and** λ_{A01} **are all positive sums of kinetic constants and species concentrations), we can cancel it in the numerator and the denominator, without making any assumptions about the steady state.**

$$\lambda_{A01} = \frac{P2_{Total}}{P1_{Total}}$$

Thus,

$$\lambda_{A10} = \frac{P1_{Total}}{P2_{Total}}$$

Solving these equations for their respective substrate concentrations we find that in an asymmetric state, the concentrations of [A01] and [A10], the partially modified substrates are fixed and is given by few key kinetic constants.

$$A01 := -\frac{P2_{Total}\, k_1}{c_2\,(P2_{Total}\, k_1 - k_2\, P1_{Total})} = -\frac{P2_{Total}\, k_1}{c_2\,(k_1\, P2_{Total} - k_2\, P1_{Total})}$$

$$A10 := -\frac{P1_{Total}\, a_1}{d_2\,(P1_{Total}\, a_1 - a_2\, P2_{Total})} = -\frac{P1_{Total}\, a_1}{d_2\,(a_1\, P1_{Total} - a_2\, P2_{Total})}$$

Necessary Conditions

Since substrate concentration are always necessarily positive, the expression for the concentrations of [A00] and [A11] should be positive. The numerator is only a function of kinetic parameters and total enzyme concentrations which are always positive, thus the denominator must necessarily be positive to ensure that the resulting concentration is positive. This gives us the necessary condition for the asymmetric state to exist as follows.

1. $k_2 \cdot P1_{Total} > k_1 \cdot P2_{Total}$
2. $a_2 \cdot P2_{Total} > a_1 \cdot P1_{Total}$

Sufficiency of necessary conditions

In this section we show that the necessary conditions generated above are sufficient to ensure symmetry breaking at some finite A_{Total}.

As shown above, should an asymmetric branch exist the associated invariants (of concentrations) need to be true irrespective of A_{Total} values. To show that the asymmetric state is a feasible steady state for the system, we need to show that the concentrations of all variables involved (substrates, complexes and free enzymes) are all positive. Simultaneoulsy we need ensure that the system satisfies the conservation conditions associated with the concentrations of the substrate and the respective enzymes.

We show this in the following manner.

1. We evaluate the steady state of the system to obtain expressions of concentration of all substrates and complexes in terms of a few key concentrations ([A01], [K1], [P1], [K2], [P2] as done earlier).
Note: This is done by running the code until before the section 'Proof for invariant in asymmetric branches'

2. We then substitute one of the invariants [A01] concentration into the system.

$$A01 := -\frac{P2_{Total}\, k_1}{c_2\,\left(P2_{Total}\, k_1 - k_2\, P1_{Total}\right)} :$$

3. Further we solve the conservation expressions K1Con and P1Con, for expressions of the free enzymes [K1] and [P1] in terms of the concentrations of other free enzymes ([K2] and [P2]) and the invariant substituted earlier for [A01] concentration.

$$P1 := solve(P1Con, P1) :$$
$$K1 := solve(K1Con, K1) :$$

We can thus ascertain concentrations of the following substrates as a function of free enzymes [K2], [P2] and constants. Here we introduce a new ratio $\alpha = \frac{K2}{P2}$

$$K2 := \alpha \cdot P2 :$$

$$simplify(A10) = -\frac{P1_{Total}\, a_1}{\left(a_1\, P1_{Total} - a_2\, P2_{Total}\right) d_2}$$

$$simplify(K1) = -\frac{P2_{Total}\, \alpha\, d_1\, \left(a_1\, P1_{Total} - a_2\, P2_{Total}\right)}{\left(\alpha\, d_1\, P2_{Total} + c_1\, P1_{Total}\right) a_2}$$

$$simplify(P1) = -\frac{P1_{Total}\, \left(P2_{Total}\, k_1 - k_2\, P1_{Total}\right) c_1}{k_2\, \left(\alpha\, d_1\, P2_{Total} + c_1\, P1_{Total}\right)}$$

$$simplify(A00) = -\frac{P1_{Total}\, a_2}{\alpha\, d_1\, \left(a_1\, P1_{Total} - a_2\, P2_{Total}\right)}$$

$$simplify(A11) = -\frac{\alpha\, P2_{Total}\, k_2}{\left(P2_{Total}\, k_1 - k_2\, P1_{Total}\right) c_1}$$

We can see that automatically we get the second invariant for the concentration of [A10] to be true. From the expressions of the variables we note that when [K2] and [P2] are both positive the variable concentrations are positive.

4. Now we solve K2Con (the conservation condition associated with the enzyme [K2]) for the concentration of free enzyme [P2] and get the following correlation.

$P2 := simplify(solve(P2Con, P2)) =$

$$P2 := -\frac{(a_1 P1_{Total} - a_2 P2_{Total})(P2_{Total} k_1 - k_2 P1_{Total})}{k_2 (a_1 \alpha - a_2) P1_{Total} + a_2 P2_{Total} (-\alpha k_2 + k_1)} \quad (1.3.1)$$

Note that the denominator can be simplified as
$\alpha k_2 (a_1 P1_{Total} - a_2 P2_{Total}) + a_2 (k_1 P2_{Total} - k_2 P1_{Total})$

So again, if α is positive, [P2] is positive implying [K2] is positive.

In an asymmetric condition we have no restriction on the value that this α can take. Note that in a symmetric state, by mandating [A01] = [A10] we fix the value of α in terms of kinetic constants and total enzyme concentrations.

$simplify(K1Con) = 0$
$simplify(K2Con) = 0$
$simplify(P1Con) = 0$
$simplify(P2Con) = 0$

$simplify(dA00) = 0$
$simplify(dA11) = 0$
$simplify(dA01) = 0$
$simplify(dA10) = 0$
$simplify(dA00K1) = 0$
$simplify(dA00K2) = 0$
$simplify(dA01P1) = 0$
$simplify(dA10P2) = 0$
$simplify(dA01K2) = 0$
$simplify(dA10K1) = 0$
$simplify(dA11P1) = 0$
$simplify(dA11P2) = 0$
$simplify(dK2) = 0$
$simplify(dK1) = 0$
$simplify(dP1) = 0$
$simplify(dP2) = 0$

We can thus ensure that for any positive value of α, all concentrations are positive. This thus implies that there exists a finite positive ATotal value where this asymmetric state is realized.

Hence we have shown that the necessary conditions are sufficient for symmetry breaking at some finite A_{Total} value.

Position of symmetry breaking (Pitchfork bifurcation)

At the position of symmetry breaking, we know two insights.
1. The system is still symmetric, hence [A00] = [A11], [K1] = [P2], [P1] = [K2]

2. The invariants descibing the asymmetric steady state are also true.

Using these two information, we can simplify the original system considerably as follows. Note:

$K1 := P2:$
$P1 := K2:$

$$A00 = \frac{K2\, A01\, c_2\, k_2}{P2\, c_1\, k_1}$$
$$A01 = A01$$
$$A10 = \frac{A01\, K2^2\, a_1\, c_2\, d_1\, k_2}{P2^2\, c_1\, k_1\, a_2\, d_2}$$
$$A11 = \frac{K2\, A01\, c_2\, k_2}{P2\, c_1\, k_1}$$

But we also know that [A01] and [A10] are invariants given by the following expressions.

$$A01 := -\frac{P2_{Total}\, k_1}{c_2\,(P2_{Total}\, k_1 - k_2\, P1_{Total})} = -\frac{P2_{Total}\, k_1}{c_2\,(k_1\, P2_{Total} - k_2\, P1_{Total})}$$
$$A10 := -\frac{P1_{Total}\, a_1}{d_2\,(P1_{Total}\, a_1 - a_2\, P2_{Total})} \quad -\frac{P1_{Total}\, a_1}{d_2\,(a_1\, P1_{Total} - a_2\, P2_{Total})}$$

Using this information we can find the value of [K2] and [P2]. Substituiting this into the expressions for conservation of the individual enzymes, we get the concentrations of the respective free enzymes.

$A_{Total} = simplify(solve(ACon, A_{Total}))$

$$A_{Total} = \Bigg(-2\, a_2 \bigg(\Big(P2\,(k_2 + k_1)\, c_1 + k_2\,(K2\, d_1 + 1)\Big)\, K2\, c_2 + \frac{P2\, c_1\, k_1}{2} \bigg)\, d_2\, P2_{Total}^{\,2} + 2\, P1_{Total} \bigg(\Big(\big(P2\,(k_1\, P2 + K2\,(k_2 + k_1))\, c_1 + K2\, k_2\,(K2\, d_1 + 1)\big)\, d_2 + \frac{P2\, c_1\, k_1}{2} \Big)\, c_2 + \frac{P2\, c_1\, d_2\, k_1}{2} \bigg)\, a_1\, P2_{Total} - 2\, P1_{Total}^{\,2}\, P2\, c_1 \Big(P2\, d_2 + \frac{1}{2}\Big)\, a_1\, k_2\, c_2 \Bigg) \Big/ \Big((-P2_{Total}\, k_1 + k_2\, P1_{Total})\, c_1\, P2\, d_2\, (P1_{Total}\, a_1 - a_2\, P2_{Total})\, c_2 \Big) \quad \textbf{(1.4.1)}$$

An example of this is done in the read me file using the parameters used in generating the

figures. A cross verification of the analytical work and bifurcation analysis is done in this manner.

Case 2 Symmetry : Present and Breaks

Kinetic constraints for case 2 symmetry to be present (refer main text) are imposed on the original model.

$a_1 := k_1 : a_{b1} := k_{b1} : a_{ub1} := k_{ub1} :$
$a_2 := k_2 : a_{b2} := k_{b2} : a_{ub2} := k_{ub2} :$
$a_3 := k_3 : a_{b3} := k_{b3} : a_{ub3} := k_{ub3} :$
$a_4 := k_4 : a_{b4} := k_{b4} : a_{ub4} := k_{ub4} :$

In addition to the kinetic constraints the total enzyme concentrations of kinase and phosphatase need to be equal for exact case 2 symmetry to be present. This is imposed as shown below.

$K1_{Total} := K_{Total} :$
$K2_{Total} := K_{Total} :$
$P1_{Total} := P_{Total} :$
$P2_{Total} := P_{Total} :$

At this stage we introduce auxiliary constants c_1, c_2, c_3 and c_4 in place of the binding constants so as to make further analytical expressions more accessible.

$k_{b1} := c_1 \cdot (k_1 + k_{ub1}) :$
$k_{b2} := c_2 \cdot (k_2 + k_{ub2}) :$
$k_{b3} := c_3 \cdot (k_3 + k_{ub3}) :$
$k_{b4} := c_4 \cdot (k_4 + k_{ub4}) :$

Once this is done, we solve for the steady state of the system in terms of fewer key variables. In this context we want to solve all variables in terms of the concentrations of the free enzymes ([K1], [K2], [P1] & [P2]) and concentration of the completely unmodified substrate [A00]. In order to do this, we use the Maple command *solve,* which algebraically solves the equation supplied for a given variable. We first solve for the individual complexes using their corresponding differential equation. An example of this (using [A00K1]) is given below in detail.

The differential equation of [A00K1] is given by,

$$\frac{d\,[A00K1]}{dt} = dA00K1 \qquad \frac{d\,[A00K1]}{dt} = c_1\,(k_1 + k_{ub1})\,A00\,K1 - (k_1 + k_{ub1})\,A00K1$$

The solve command by Maple, uses this solves this equation for the given variable (in this case [A00K1]). This is performed by the following command. Here we assign [A00K1], the solution

returned by the solve command.

$A00K1 := solve(dA00K1, A00K1)$

$$A00K1 := K1\ A00\ c_1 \qquad (2.1)$$

This operation is performed for the other complexes and substrate forms as well. Here we simultaneously *solve* expressions for the substrate forms using the solve command as shown below. The solution is stored in a variable labelled *Sol*, and then the respective solutions are extracted from this vector using the *eval* command.

$A01K2 := solve(dA01K2, A01K2):$
$A11P2 := solve(dA11P2, A11P2):$
$A01P1 := solve(dA01P1, A01P1):$
$A00K2 := solve(dA00K2, A00K2):$
$A10K1 := solve(dA10K1, A10K1):$
$A11P1 := solve(dA11P1, A11P1):$
$A10P2 := solve(dA10P2, A10P2):$

$Sol := solve(\{dA01, dA10, dA11\}, \{A01, A10, A11\}):$

$A00 := eval(A00, Sol):$
$A01 := eval(A01, Sol):$
$A10 := eval(A10, Sol):$
$A11 := eval(A11, Sol):$

Doing this results in the following correlations between the concentrations of the various substrate forms at steady state.

$A00 = A00$

$$A01 = \frac{A00\ K1\ c_1\ k_1}{c_4\ k_4\ P1}$$

$$A10 = \frac{K2\ A00\ c_1\ k_1}{P2\ c_4\ k_4}$$

$$A11 = \frac{A00\ K1\ K2\ c_1\ c_2\ k_1\ k_2}{P2\ c_4\ k_4\ P1\ c_3\ k_3}$$

Proof for invariant in asymmetric branches

We know that $P1_{Total}$*$K2_{Total}$ = $K1_{Total}$*$P2_{Total}$ in this system under case 1 symmetry (Since $K1_{Total}$ = $K2_{Total}$ and $P2_{Total}$ = $P1_{Total}$). Thus at a given steady state, ($P1_{Total}$* $K2_{Total}$ = $K1_{Total}$*$P2_{Total}$) must be equal to 0. We introduce a term T = ($P1_{Total}$*$K2_{Total}$ = $K1_{Total}$*$P2_{Totall}$) as shown below. Here we write $P1_{Total}$ as P1Con + $P1_{Total}$. Note: This comes from the expression used for P1Con earlier and the fact that PCon = 0. This way we have represented the independent enzyme concentrations as a sum of the free enzyme and the complexes in which it is sequestered.

Similarly for the other total enzyme concentrations as well.

$$T := (P1Con - P1_{Total}) \cdot (K2Con - K2_{Total}) - (P2Con - P2_{Total}) \cdot (K1Con - K1_{Total}) = 0 :$$

This way we have represented the independent enzyme concentrations as a sum of the free enzyme and the complexes in which it is sequestered.

At this stage we evaluate the ratio of [A01] and [A10]. Note that with case 2 symmetry breaking, we are looking for asymmetric states such that [A01] not equal to [A10].

$$simplify\left(\frac{A01}{A10}\right) = \frac{K1\ P2}{P1\ K2}$$

We denote this ratio as ϵ. i.e. ϵ = (P2*K1)/(K2*P1).

$$P2 := \frac{\epsilon \cdot P1 \cdot K2}{K1} :$$

$$simplify\left(\frac{A01}{A10}\right) = \epsilon =$$

After introducing this ratio and algebraically simplifying T using the Maple command *simplify*, we get the following equation.

$$simplify(T)$$

$$\frac{1}{P1\ c_4\ k_4^2\ k_3\ \epsilon}\Big((\epsilon - 1)\big(-k_3\ \epsilon\ P1^2\ c_4\ (A00\ c_1 + 1)\ k_4^2 - K1^2\ A00\ c_1\ c_2\ k_1\ k_2\ (A00\ c_1 + 1)\ k_4 + A00^2\ K1^2\ c_1^2\ c_2\ k_1^2\ k_3\big)\ K2\Big) = 0 \quad \textbf{(2.1.1)}$$

From this we can ascertain that, should an asymmetric steady state exist (where $\epsilon \neq 1$) - the term (
$-k_3\ \epsilon\ P1^2\ c_4\ (A00\ c_1 + 1)\ k_4^2 - K1^2\ A00\ c_1\ c_2\ k_1\ k_2\ (A00\ c_1 + 1)\ k_4 + A00^2\ K1^2\ c_1^2\ c_2\ k_1^2\ k_3$**)**
in the numerator of the expression needs to be zero. This term is an expression in the partial substrate form [A00], ϵ, the free enzymes and kinetic constants.

We show here that this term is nothing but the numerator of the following expression multiplied by the ratio ($\epsilon\ P1^2\ c_4\ k_4$) where [A01] written as a function of [A10].

$$r := \left(\left(\frac{k_3 \cdot c_3 \cdot A11}{k_2 \cdot (1 + c_3 \cdot A11)}\right) \cdot \left(\frac{k_1 \cdot c_1 \cdot A00}{k_4 \cdot (1 + c_1 \cdot A00)}\right) - 1\right) :$$

This is verified below. The *numer* command in Maple isolates only the numerator of an expression. In this context we isolate the numerator of 'r', divide it by the factor ($\epsilon\ P1^2\ c_4\ k_4$) and add the term denoting asymmetry from earlier. We can see that all expressions in the

term cancel out revealing that they are identical.

$$factor\left(-k_3\,\epsilon\, P1^2\, c_4\, \left(A00\, c_1 + 1\right) k_4^{\,2} - K1^2\, A00\, c_1\, c_2\, k_1\, k_2\, \left(A00\, c_1 + 1\right) k_4 + A00^2\, K1^2\, c_1^{\,2}\, c_2\, k_1^{\,2}\, k_3 - \frac{(numer(r))}{\epsilon\, P1^2\, c_4\, k_4} \right)$$

Thus, here we have a clear representation of the asymmetric steady state in terms of a correlation between only the concentrations of the partially modified enzymes and key kinetic constants (i.e. r = 0). r in turn can be rewritten for simplicity as follows.

$restart:$
$r := \lambda_{A00}\cdot\lambda_{A11} - 1:$

Where the λ_{A00} and λ_{A11} expressions are given as follows.

$$\lambda_{A11} := \frac{k_3\cdot c_3\cdot A11}{k_2\cdot\left(1 + c_3\cdot A11\right)}:$$

$$\lambda_{A00} := \frac{k_1\cdot c_1\cdot A00}{k_4\cdot\left(1 + c_1\cdot A00\right)}:$$

Now we use an auxiliary analysis to ascertain other correlations between [A00] and [A11] that is valid for all steady states of the system (not just asymmetric ones). We refresh Maple here to rewrite some new expressions.

$restart:$

We begin by evaluating the following ratio of enzymes $\frac{\left(P1_{Total} + P2_{Total}\right)}{\left(K1_{Total} + K2_{Total}\right)}$. We know from the individual conservation equations that they correspond to the sum of the free enzyme and the complexes in which they are sequestered. Thus,

$$\frac{\left(P1_{Total} + P2_{Total}\right)}{\left(K1_{Total} + K2_{Total}\right)} = \frac{(A11P1 + A01P1 + P1) + (A10P2 + A11P2 + P2)}{(A00K1 + A10K1 + K1) + (A01K2 + A00K2 + K2)}:$$

Now from the dynamical representation of the network as a system of ODEs earlier (particularly equations dA01 and dA10) we know that at steady state,

$$A01P1 + A10P2 = \frac{k_1}{k_4}\cdot(A00K1 + A00K2):$$

$$A01K2 + A10K1 = \frac{k_3}{k_2}\cdot(A11P1 + A11P2) :$$

Thus,

$$\frac{(P1_{Total} + P2_{Total})}{(K1_{Total} + K2_{Total})} = \frac{\frac{k_1}{k_4}\cdot(A00K1 + A00K2) + P1 + P2 + A11P1 + A11P2}{\frac{k_3}{k_2}\cdot(A11P1 + A11P2) + A00K1 + K1 + K2 + A00K2} :$$

This can be further simplified as follows, (by rewriting the concentration of the complexes at steady state and simplifying the resulting expression)

$$\frac{(P1_{Total} + P2_{Total})}{(K1_{Total} + K2_{Total})} = \frac{\frac{k_1}{k_4}\cdot c_1\cdot A00\cdot(K1 + K2) + (1 + c_3\cdot A11)\cdot(P1 + P2)}{\frac{k_3}{k_2}\cdot c_3\cdot A11\cdot(P1 + P2) + (1 + c_1\cdot A00)\cdot(K1 + K2)} :$$

Now we introduce a new ratio, $\alpha = \frac{(1 + c_1\cdot A00)\cdot(K1 + K2)}{(1 + c_3\cdot A11)\cdot(P1 + P2)}$ **Substituting this ratio in the above expression we get the following simplified form,**

$$\frac{(P1_{Total} + P2_{Total})}{(K1_{Total} + K2_{Total})} = \frac{\frac{k_1}{k_4}\cdot\frac{c_1\cdot A00\cdot\alpha}{(1 + c_1\cdot A00)} + 1}{\frac{k_3}{k_2}\cdot\frac{c_3\cdot A11}{(1 + c_3\cdot A11)} + \alpha} :$$

Reintroducting the nomenclature of λ_{A00} **and** λ_{A11} **the above equation simplifies as,**

$$\frac{(P1_{Total} + P2_{Total})}{(K1_{Total} + K2_{Total})} = \frac{\lambda_{A00}\cdot\alpha + 1}{\lambda_{A11} + \alpha} :$$

Under case 2 symmetry, $K2_{Total} = K1_{Total}$**, and** $P1_{Total} = P2_{Total}$**. Here we bring in the asymmetry correlation ascertained earlier, i.e. r = 1 or** $\lambda_{A00} = \frac{1}{\lambda_{A11}}$ **. Using these two insights, the above equation simplifies to**

$$\frac{(P_{Total})}{(K_{Total})} = \frac{(\lambda_{A00}\cdot\alpha\cdot D + 1)}{(1 + \alpha\cdot D\cdot\lambda_{A00})}\cdot\lambda_{A00} :$$

Since the expression on the right is always positive (α and λ_{A00} are all positive sums of kinetic constants and species concentrations), we can cancel it in the numerator and the denominator, without making any assumptions about the steady state.

$$\lambda_{A00} := \frac{P_{Total}}{K_{Total}} :$$

Thus

$$\lambda_{A11} := \frac{K_{Total}}{P_{Total}} :$$

Solving these equations for their respective substrate concentrations we find that in an asymmetric state, the concentrations of [A00] and [A11], the partially modified substrates are fixed and is given by few key kinetic constants.

$$A00 = solve\left(\frac{k_1 \cdot c_1 \cdot A00}{k_4 \cdot (1 + c_1 \cdot A00)} = \frac{P_{Total}}{K_{Total}}, A00\right)$$

$$A00 = \frac{P_{Total}\, k_4}{c_1\, (k_1\, K_{Total} - P_{Total}\, k_4)} \tag{2.1.2}$$

$$A11 = solve\left(\frac{k_3 \cdot c_3 \cdot A11}{k_2 \cdot (1 + c_3 \cdot A11)} = \frac{K_{Total}}{P_{Total}}, A11\right)$$

$$A11 = -\frac{K_{Total}\, k_2}{c_3\, (K_{Total}\, k_2 - k_3\, P_{Total})} \tag{2.1.3}$$

Necessary conditions

Since substrate concentration are always necessarily positive, the expression for the concentrations of [A00] and [A11] should be positive. The numerator is only a function of kinetic parameters and total enzyme concentrations which are always positive, thus the denominator must necessarily be positive to ensure that the resulting concentration is positive. This gives us the necessary condition for the asymmetric state to exist as follows.

1. $k_1 \cdot K_{Total} > k_4 \cdot P_{Total}$
2. $k_3 \cdot P_{Total} > k_2 \cdot K_{Total}$

Sufficiency of necessary conditions

In this section we show that the necessary conditions generated above are sufficient to ensure symmetry breaking at some finite A_{Total}.

As shown above, should an asymmetric branch exist the associated invariants (of concentrations) need to be true irrespective of A_{Total} values. To show that the asymmetric state is a feasible steady state for the system, we need to show that the concentrations of all variables involved (substrates, complexes and free enzymes) are all positive. Simultaneoulsy we need ensure that the system satisfies the conservation conditions associated with the concentrations of the substrate and the respective enzymes.

We show this in the following manner.

1. We evaluate the steady state of the system to obtain expressions of concentration of all substrates and complexes in terms of a few key concentrations ([A00], [K1], [P1], [K2], [P2] as done earlier).
Note: This is done by running the code until before the section 'Proof for invariant in asymmetric branches'

2. We then substitute one of the invariants [A00] concentration into the system.

$$A00 := \frac{P_{Total}\, k_4}{c_1\,\left(k_1\, K_{Total} - P_{Total}\, k_4\right)} :$$

3. Further we solve the conservation expressions K1Con and P1Con, for expressions of the free enzymes [K1] and [P1] in terms of the concentrations of other free enzymes ([K2] and [P2]) and the invariant substituted earlier for [A00] concentration.

$$K1 := simplify(solve(K1Con, K1)) :$$
$$P1 := simplify(solve(P1Con, P1)) :$$

We can thus ascertain concentrations of the following substrates as a function of free enzymes [K2], [P2] and constants. Here wee introduce a new ratio $\alpha = \frac{K2}{P2}$

$$K2 := \alpha \cdot P2 :$$

$$simplify(A11) = -\frac{K_{Total}\, k_2}{\left(k_2\, K_{Total} - k_3\, P_{Total}\right) c_3}$$

$$simplify(K1) = \frac{K_{Total}\,\left(k_1\, K_{Total} - P_{Total}\, k_4\right) c_4}{k_1\,\left(c_2\, \epsilon\, P_{Total} + c_4\, K_{Total}\right)}$$

$$simplify(P1) = -\frac{P_{Total}\, \epsilon\, c_2\,\left(k_2\, K_{Total} - k_3\, P_{Total}\right)}{\left(c_2\, \epsilon\, P_{Total} + c_4\, K_{Total}\right) k_3}$$

$$simplify(A10) = \frac{\epsilon\, P_{Total}\, k_1}{\left(k_1\, K_{Total} - P_{Total}\, k_4\right) c_4}$$

$$simplify(A01) = -\frac{K_{Total}\, k_3}{\epsilon\, c_2\,\left(k_2\, K_{Total} - k_3\, P_{Total}\right)}$$

We can see that automatically we get the second invariant for the concentration of [A11] to

be true. From the expressions of the variables we note that when [K2] and [P2] are both positive the variable concentrations are positive.

4. Now we solve K2Con (the conservation condition associated with the enzyme [K2]) for the concentration of free enzyme [P2] and get the following correlation.

$P2 := simplify(solve(P2Con, P2))$

$$P2 := \frac{(k_2 K_{Total} - k_3 P_{Total})(k_1 K_{Total} - P_{Total} k_4)}{k_1 (\epsilon k_2 - k_3) K_{Total} - k_3 P_{Total} (\epsilon k_1 - k_4)} \qquad \textbf{(2.3.1)}$$

Note that the denominator can be simplified as
$\epsilon k_1 (k_2 K_{Total} - k_3 P_{Total})$ -$k_3 (k_1 K_{Total} - P_{Total} k_4)$

So again, if α is positive, [P2] is positive implying [K2] is positive.

Note that in a symmetric state, by mandating [A01] = [A10] we fix the value of α in terms of kinetic constants and total enzyme concentrations.

$simplify(K1Con) = 0$
$simplify(K2Con) = 0$
$simplify(P1Con) = 0$
$simplify(P2Con) = 0$

$simplify(dA00) = 0$
$simplify(dA11) = 0$
$simplify(dA01) = 0$
$simplify(dA10) = 0$
$simplify(dA00K1) = 0$
$simplify(dA00K2) = 0$
$simplify(dA01P1) = 0$
$simplify(dA10P2) = 0$
$simplify(dA01K2) = 0$
$simplify(dA10K1) = 0$
$simplify(dA11P1) = 0$
$simplify(dA11P2) = 0$
$simplify(dK2) = 0$
$simplify(dK1) = 0$
$simplify(dP1) = 0$
$simplify(dP2) = 0$

We can thus ensure that for any positive value of α, all concentrations are positive. This thus implies that there exists a finite positive A_{Total} value where this asymmetric state is realized.

Hence we have shown that the necessary conditions are sufficient for symmetry breaking at

some finite A_{Total} value.

Position of symmetry breaking (Pitchfork bifurcation)

At the position of symmetry breaking, we know two insights.
1. The system is still symmetric, hence [A01] = [A10], [K1] = [K2], [P1] = [P2]
2. The invariants descibing the asymmetric steady state are also true.

Using these two information, we can simplify the original system considerably as follows.
Note:

$K1 := K2:$
$P1 := P2:$

$$A00 = A00$$
$$A01 = \frac{K2\, A00\, c_1\, k_1}{P2\, c_4\, k_4}$$
$$A10 = \frac{K2\, A00\, c_1\, k_1}{P2\, c_4\, k_4}$$
$$A11 = \frac{A00\, K2^2\, c_1\, c_2\, k_1\, k_2}{P2^2\, c_4\, k_4\, c_3\, k_3}$$

But we also know that [A00] and [A11] are invariants given by the following expressions.

$$A00 := \frac{P_{Total}\, k_4}{c_1\, (k_1\, K_{Total} - P_{Total}\, k_4)}:$$
$$A11 := -\frac{K_{Total}\, k_2}{(k_2\, K_{Total} - k_3\, P_{Total})\, c_3}:$$

Using this information we can find the value of [K2] and [P2]. Substituiting this into the expressions for conservation of the individual enzymes, we get the concentrations of the respective free enzymes.

$$A_{Total} = simplify(solve(ACon, A_{Total}))$$

$$A_{Total} = \frac{1}{P2^2\, c_4\, k_4\, c_3\, k_3}\left(2\left(P2\left(\left(K2\,(k_4+k_1)\,c_1+\frac{k_4}{2}\right)c_4\,k_3\,P2+\left(\left(K2\,c_2+1\right)k_3+K2\,c_2\,k_2\right)c_1\,k_1\,K2\right)c_3+\frac{K2^2\,c_1\,c_2\,k_1\,k_2}{2}\right)A00\right) \quad \textbf{(2.4.1)}$$

An example of this is done in the read me file using the parameters used in generating the figures. A cross verification of the analytical work and bifurcation analysis is done in this manner.

Case 3 Symmetry : Present and Breaks (Arguments for necessary conditions)

Unlike case 3 symmetry breaking in other models of Random DSP with common enzymes or case 1-2 symmetry breaking in System 3 Random DSP with separate kinases and phosphatases, there exists no simple linear invariant of concentrations at asymmetric steady states post symmetry breaking (upon bifurcation along A_{Total})

However the asymmetric steady states in this case 3 System 3 Random DSP exhibit their own signature of symmetry breaking in the form of asymptotic values for concentrations of specific substrates to increasing A_{Total}. These asymptotic values manifest as approximate concentration robustness for concentrations of the specific substrates.

In this analysis below we show the presence of such symmetries and how the underlying kinetics dictates both the specific pair that exhibits asymptotic response to increasing A_{Total} and the individual asymptote concentrations.

Through this process we also obtain necessary conditions for case 3 symmetry to break in System 3 Random DSP (including the necessary conditions for approximate robustness in relevant substrate concentrations through asymptotic behavior)

Kinetic constraints for case 3 symmetry (refer main text) are imposed on the original model.

$$a_3 := k_1 : a_{b3} := k_{b1} : a_{ub3} := k_{ub1} :$$
$$a_4 := k_2 : a_{b4} := k_{b2} : a_{ub4} := k_{ub2} :$$
$$a_1 := k_3 : a_{b1} := k_{b3} : a_{ub1} := k_{ub3} :$$
$$a_2 := k_4 : a_{b2} := k_{b4} : a_{ub2} := k_{ub4} :$$

In addition to the kinetic constraints the total enzyme concentrations of kinase and phosphatase also need to be equal for exact case 3 symmetry to be present. This is imposed as shown below.

$$K1_{Total} := P1_{Total} :$$
$$K2_{Total} := P2_{Total} :$$

At this stage we introduce auxiliary constants c_1, c_2, c_3 and c_4 in place of the binding constants so as to make further analytical expressions more accessible.

$$k_{b1} := c_1 \cdot (k_1 + k_{ub1}) :$$
$$k_{b2} := c_2 \cdot (k_2 + k_{ub2}) :$$
$$k_{b3} := c_3 \cdot (k_3 + k_{ub3}) :$$

$k_{b4} := c_4 \cdot (k_4 + k_{ub4}) :$

Once this is done, we solve for the steady state of the system in terms of fewer key variables. In this context we want to solve all variables in terms of the concentrations of the free enzymes ([K1], [K2], [P1] & [P2]) and concentration of the substrates. In order to do this, we use the Maple command *solve,* which solves the equation supplied for a given variable. We first solve for the individual complexes using their corresponding differential equation. An example of this (using [A00K1]) is given below in detail.

The differential equation of [A00K$_1$] is given by,

$$\frac{d\,[A00K1]}{dt} = dA00K1$$

$$\frac{d\,[A00K1]}{dt} = c_1\,(k_1 + k_{ub1})\,A00\,K1 - (k_1 + k_{ub1})\,A00K1 \tag{3.1}$$

The *solve* command by Maple, solves this equation for the given variable (in this case [A00K1]). We in turn store this value (the solution returned by the *solve* command) in [A00K$_1$]. This is performed by the following command.

$A00K1 := solve(dA00K1, A00K1)$

$$A00K1 := K1\,A00\,c_1 \tag{3.2}$$

This operation is performed for the other complexes as well as shown below.

$A01K2 := solve(dA01K2, A01K2) :$
$A11P2 := solve(dA11P2, A11P2) :$
$A01P1 := solve(dA01P1, A01P1) :$
$A00K2 := solve(dA00K2, A00K2) :$
$A10K1 := solve(dA10K1, A10K1) :$
$A11P1 := solve(dA11P1, A11P1) :$
$A10P2 := solve(dA10P2, A10P2) :$

At this stage we solve for the individual free enzyme concentrations of [P1] and [P2] as shown below. Note that since K1Con, K2Con, P1Con and P2Con are all equal to zero at any given steady state, K1Con-P1Con and K2Con-P2Con is also equal to zero. We define these terms as T = K1Con-P1Con and Q = K2Con-P2Con and use these expressions to solve for [P1] and [P2] as shown below

$T := K1Con - P1Con :$
$Q := K2Con - P2Con :$

$P1 := solve(T, P1)$

$$P1 := \frac{K1\,(A00\,c_1 + A10\,c_4 + 1)}{A01\,c_4 + A11\,c_1 + 1} \tag{3.3}$$

$P2 := solve(Q, P2)$

$$P2 := \frac{K2\left(A00\, c_3 + A01\, c_2 + 1\right)}{A10\, c_2 + A11\, c_3 + 1} \quad (3.4)$$

Now at this stage we simplify the remaining unsolved equations from the system of ODE description of the model (dA00, dA01, dA10, dA11). (Note: dP1, dP2, dK1, dK2 are all equal to zero since we solved the respective complex equations in the sytem of ODE description)

We definte two terms, M = dA00 + dA01 and N = dA00 + dA10. Note that since dA00, dA01 and dA10 are each equally to zero at any given steady state, M and N are also equal to zero.

$M := simplify(dA00 + dA01)$

$$M := \frac{1}{A10\, c_2 + A11\, c_3 + 1}\left(K2\left(\left(\left(k_2 - k_3\right)\left(A00\, A10 - A01\, A11\right) c_3 - k_2\left(A01 - A10\right)\right) c_2 - c_3\, k_3\left(A00 - A11\right)\right)\right) \quad (3.5)$$

$N := simplify(dA00 + dA10)$

$$N := -\frac{1}{A01\, c_4 + A11\, c_1 + 1}\left(K1\left(\left(\left(k_1 - k_4\right)\left(A00\, A01 - A10\, A11\right) c_4 + k_1\left(A00 - A11\right)\right) c_1 - c_4\, k_4\left(A01 - A10\right)\right)\right) \quad (3.6)$$

Thus from M and N we can ascertain that for any given feasible steady stae the following expressions styled m and n need to be equal to zero.

$m := \left(\left(k_2 - k_3\right)\left(A00\, A10 - A01\, A11\right) c_3 - k_2\left(A01 - A10\right)\right) c_2 - c_3\, k_3\left(A00 - A11\right) :$

$n := \left(\left(k_1 - k_4\right)\left(A00\, A01 - A10\, A11\right) c_4 + k_1\left(A00 - A11\right)\right) c_1 - c_4\, k_4\left(A01 - A10\right) :$

At this stage, we introduce the ratio $\epsilon = \dfrac{A00}{A11}$ **and thus m and n simplifies as shown below**

$A00 := \text{epsilon} \cdot A11 :$

$simplify(m)$

$$\left(-A11\left(k_2 - k_3\right)\left(-A10\,\epsilon + A01\right) c_3 - k_2\left(A01 - A10\right)\right) c_2 - A11\, c_3\, k_3\left(\epsilon - 1\right) \quad (3.7)$$

$simplify(n)$

$$A11\left(\left(k_1 - k_4\right)\left(A01\,\epsilon - A10\right) c_4 + k_1\left(\epsilon - 1\right)\right) c_1 - c_4\, k_4\left(A01 - A10\right) \quad (3.8)$$

We first solve for the concentration of [A11] from m as shown below.

$A11 := simplify(solve(M, A11))$

$$A11 := -\frac{c_2\, k_2\left(A01 - A10\right)}{\left(\left(k_2 - k_3\right)\left(-A10\,\epsilon + A01\right) c_2 + k_3\left(\epsilon - 1\right)\right) c_3} \quad (3.9)$$

Substituiting this back in n, we get the following

$simplify(n)$

$$\left(\left(\left(\left(\left(\left((A01\,c_1 + A10\,c_3)\,k_4 - A01\,c_1\,k_1\right)\epsilon + \left(-A01\,c_3 - A10\,c_1\right)k_4 + A10\,c_1\,k_1\right)k_2 + k_4\,c_3\,k_3\,(-A10\,\epsilon + A01)\right)c_4 - c_1\,k_1\,k_2\,(\epsilon - 1)\right)c_2 - k_4\,c_3\,c_4\,k_3\,(\epsilon - 1)\right)(A01 - A10)\right) \Big/ \left(c_3\left(\left(k_2 - k_3\right)(-A10\,\epsilon + A01)\,c_2 + k_3\,(\epsilon - 1)\right)\right) \tag{3.10}$$

We now solve this expression for [A01]. Note that since we are isolating solutions of asymmetry (i.e. [A01] ≠ [A10]), we ignore the symmetric solution while solving the system as shown below.

$A01 := simplify(solve(N, A01)[2])$

$$A01 := \left(c_2\left(A10\,c_4\left(c_3\,\epsilon - c_1\right)k_4 + c_1\,k_1\left(A10\,c_4 - \epsilon + 1\right)\right)k_2 - c_3\,c_4\,k_3\,k_4\left(A10\,c_2\,\epsilon + \epsilon - 1\right)\right) \Big/ \left(c_2\left(\left(\left(-c_1\,\epsilon + c_3\right)k_4 + \epsilon\,c_1\,k_1\right)k_2 - k_4\,c_3\,k_3\right)c_4\right) \tag{3.11}$$

Finally we use the expressions obtained for [A11] and [A01] to solve for the free enzymes [K1] and [K2] from their respective conservation expressions as shown below.

$simplify(A11)$

$$\frac{\left(-A10\left(c_1\,k_1\,k_2 - k_4\left(\left(c_1 + c_3\right)k_2 - c_3\,k_3\right)\right)c_4 - c_1\,k_1\,k_2\right)c_2 - c_3\,c_4\,k_3\,k_4}{c_3\,c_1\left(\left(k_2 - k_3\right)\left(A10\left(k_1 - k_4\right)(\epsilon + 1)\,c_4 + k_1\right)c_2 - \epsilon\,c_4\,k_3\left(k_1 - k_4\right)\right)} \tag{3.12}$$

$K1 := simplify(solve(simplify(K1Con), K1))$

$$K1 := \left(P1_{Total}\left(\left(k_2 - k_3\right)\left(A10\left(k_1 - k_4\right)(\epsilon + 1)\,c_4 + k_1\right)c_2 - \epsilon\,c_4\,k_3\left(k_1 - k_4\right)\right)c_3\right) \Big/ \left(\left(\left(A10\,c_3\left(k_2 - k_3\right)(\epsilon + 1)\,c_4 + \left(k_2 - k_3\right)c_3 - \epsilon\,c_1\,k_2\right)c_2 - c_3\,\epsilon\,k_3\,c_4\right)\left(A10\left(k_1 - k_4\right)c_4 + k_1\right)\right) \tag{3.13}$$

$K2 := simplify(solve(K2Con, K2))$

$$K2 := \left(c_4\,P2_{Total}\left(\left(k_1 - k_4\right)\left(A10\left(k_2 - k_3\right)(\epsilon + 1)\,c_2 - \epsilon\,k_3\right)c_4 + c_2\,k_1\left(k_2 - k_3\right)\right)c_1\left(\epsilon\,k_2\left(k_1 - k_4\right)c_1 + c_3\,k_4\left(k_2 - k_3\right)\right)\right) \Big/ \left(\left(\left(\left(\left(k_2 - k_3\right)A10\left(k_2\left(k_1 - k_4\right)c_1 + \epsilon\,c_3\,k_4\left(k_2 - k_3\right)\right)c_2 - k_3\left(k_2\left(k_1 - k_4\right)c_1 + c_3\,k_4\left(k_2 - k_3\right)\right)\epsilon\right)c_4 - c_1\,c_2\,k_1\,k_2\left(k_2 - k_3\right)(\epsilon - 1)\right)\left(\left(A10\,c_1\left(k_1 - k_4\right)(\epsilon + 1)\,c_2 + \epsilon\left(k_1 - k_4\right)c_1 + c_3\,k_4\right)c_4 + c_1\,c_2\,k_1\right)\right) \tag{3.14}$$

At this stage, by solving a majority of the system of equations describing the sytem (at an asymmetric steady state), we have also obtained correlations between concentrations of variables, primarily, concentrations of substrates and enzymes are obtained as functions of [A10] and ϵ.

$simplify(K1Con) = 0$
$simplify(K2Con) = 0$
$simplify(P1Con) = 0$
$simplify(P2Con) = 0$

$simplify(dA00K1) = 0$
$simplify(dA00K2) = 0$
$simplify(dA01P1) = 0$
$simplify(dA10P2) = 0$
$simplify(dA01K2) = 0$
$simplify(dA10K1) = 0$
$simplify(dA11P1) = 0$
$simplify(dA11P2) = 0$
$simplify(dK2) = 0$
$simplify(dK1) = 0$
$simplify(dP1) = 0$
$simplify(dP2) = 0$

At an asymmetric steady state, $\epsilon \neq 1$. Thus in order to show the presence of symmetry breaking, we need to show the presence of positive solutions to the above correlations admitting $\epsilon \neq 1$. However due to the complexity of the system this is not possible to do symbolically however can be easily verified for various kinetic values.

Necessary conditions for and location of symmetry breaking (pitchfork bifurcation)

In this section we show the necessary condition for a pitchfork bifurcation/symmetry breaking to exist. The pitchfork bifurcation is by definition the intersection of an asymmetric branch and the symmetric branch of the steady states. Thus the correlation can be easily discerned from the expressions obtained earlier for asymmetric solutions where now $\epsilon = 1$.

Thus evaluating the expressions obtained for [A11] or [A00] at epsilon =1 (At symmetric steady states [A00] = [A11])

$simplify(eval(eval(simplify(A11)), \text{epsilon} = 1))$

$$\frac{\left(-A10\left(c_1 k_1 k_2 - k_4\left(\left(c_1 + c_3\right) k_2 - c_3 k_3\right)\right) c_4 - c_1 k_1 k_2\right) c_2 - c_3 c_4 k_3 k_4}{2\left(\left(k_2 - k_3\right)\left(A10\left(k_1 - k_4\right) c_4 + \frac{k_1}{2}\right) c_2 - \frac{c_4 k_3\left(k_1 - k_4\right)}{2}\right) c_1 c_3} \quad \textbf{(3.1.1)}$$

$simplify(eval(eval(simplify(A00)), \text{epsilon} = 1))$

$$\frac{\left(-A10\left(c_1 k_1 k_2 - k_4\left(\left(c_1 + c_3\right) k_2 - c_3 k_3\right)\right) c_4 - c_1 k_1 k_2\right) c_2 - c_3 c_4 k_3 k_4}{2\left(\left(k_2 - k_3\right)\left(A10\left(k_1 - k_4\right) c_4 + \frac{k_1}{2}\right) c_2 - \frac{c_4 k_3\left(k_1 - k_4\right)}{2}\right) c_1 c_3} \quad \textbf{(3.1.2)}$$

$simplify(eval(eval(simplify(A01)), \text{epsilon} = 1))$

$$A10 \quad \textbf{(3.1.3)}$$

$simplify(eval(eval(simplify(A10)), \text{epsilon} = 1))$

$$A10 \quad \textbf{(3.1.4)}$$

The above expression for A11 can be simplified further as the term t = 0 as shown below

$restart:$

$$t := \frac{A11 - \frac{k_2}{c_3 \cdot (k_3 - k_2)}}{A10 + \frac{k_3}{(k_3 - k_2) \cdot c_2}} = \frac{-A11 + \frac{k_4}{(k_1 - k_4) \cdot c_1}}{A10 + \frac{k_1}{(k_1 - k_4) \cdot c_4}}:$$

Simplifying the above equation we get the following,

$collect(simplify(t), \{k_2, k_3, k_4, k_1\})$

$$\frac{((c_3 A11 + 1) k_2 - c_3 k_3 A11) c_2}{c_3 (c_2 k_2 A10 + (-c_2 A10 - 1) k_3)} = -\frac{c_4 (c_1 k_1 A11 + (-c_1 A11 - 1) k_4)}{((c_4 A10 + 1) k_1 - c_4 k_4 A10) c_1} \qquad (3.1.5)$$

We now use the individual conservation expressions for the enzymes [P1] and [P2]

$P1_{Total} = (1 + c_4 \cdot A10 + c_1 \cdot A11) P1:$

$P2_{Total} = (1 + c_2 \cdot A10 + c_3 \cdot A11) P2:$

This can be rearranged as shown below

$$1 + c_4 \cdot A10 = \frac{P1_{Total}}{P1} - c_1 \cdot A11:$$

$$1 + c_1 \cdot A11 = \frac{P1_{Total}}{P1} - c_4 \cdot A10:$$

$$1 + c_2 \cdot A10 = \frac{P2_{Total}}{P2} - c_3 \cdot A11:$$

$$1 + c_3 \cdot A11 = \frac{P2_{Total}}{P2} - c_2 \cdot A10:$$

Substituiting these expressions in t, we get the following simplification for t

$$l := simplify\left(\frac{\left(\left(\frac{P2_{Total}}{P2} - c_2 \cdot A10\right) k_2 - c_3 k_3 A11\right) c_2}{c_3 \left(c_2 k_2 A10 + \left(-\frac{P2_{Total}}{P2} + c_3 \cdot A11\right) k_3\right)} = -\frac{c_4 \left(c_1 k_1 A11 + \left(-\frac{P1_{Total}}{P1} + c_4 \cdot A10\right) k_4\right)}{\left(\left(\frac{P1_{Total}}{P1} - c_1 \cdot A11\right) k_1 - c_4 k_4 A10\right) c_1} \right)$$

$$l := -\frac{\left(A10\,P2\,c_2\,k_2 + A11\,P2\,c_3\,k_3 - k_2\,P2_{Total}\right)c_2}{c_3\left(A10\,P2\,c_2\,k_2 + A11\,P2\,c_3\,k_3 - k_3\,P2_{Total}\right)} = \frac{c_4\left(c_4\,k_4\,A10\,P1 + A11\,P1\,c_1\,k_1 - k_4\,P1_{Total}\right)}{\left(c_4\,k_4\,A10\,P1 + A11\,P1\,c_1\,k_1 - k_1\,P1_{Total}\right)c_1} \quad (3.1.6)$$

Analyzing the left hand side of the equation 3.1.6

Assume k3 is greater than k2. We know that the denominator is positve from equation 3.1.5. Thus the numerator is also positive (since k2<k3), thus the entire left hand side term is strictly negative.

Thus the right hand side expression has to be negative as well for the equation to be true.

Now suppose k4 > k1. Then the numerator as it appears in 3.1.6 is positive. And since k4 > k1, the denominator also has to be positive. Thus the right hand side is entirely positive.

This is a contradiction and so the equation cannot be true under this condition.

Hence, should the correlation above hold good, k1 > k4 when k3 > k2. Similarly one can reason that when k2 > k3, k4 > k1 is necessary.

Thus these become necessary kinetic constraints for symmetry breaking to be feasible. Note that these are not shown to be suffiient however extensive computational simulations show indications that these conditions are indeed sufficient for symmetry breaking to exist at some finite total substrate concentration.

The exact value at the pitchfork bifurcation point can be known by substituting equation 3.1.2 in the following correlation between the concentrations at the symmetric point involving total enzyme concentrations of [P1] and [P2].

$$E := \frac{P1_{Total}}{P2_{Total}} \cdot \frac{\left(1 + c_2 \cdot A10 + c_3 \cdot A11\right)}{\left(1 + c_4 \cdot A10 + c_1 \cdot A11\right)} - \frac{\left(c_3 \cdot k_3 \cdot A11 - c_2 \cdot k_2 \cdot A10\right)}{\left(-c_1 \cdot k_1 \cdot A11 + c_4 \cdot k_4 \cdot A10\right)} :$$

System 3 Random DSP breaks symmetry with a unique asymmetric signature in the form of asymptotes for the individual substrates. However the value of the asymptote and the substrate associated with it is given entirely by the kinetics.

Upon bifurcation along A_{Total} two scenarios are possible, as seen computationally

1. [A00] becomes 0(Infinity) as [A11] becomes Infinity(0) (On the asymmetric branches)

In this scenario, [A01] and [A10] each inidivually saturate towards an unique asymptotic value.

2. [A01] becomes 0(Infinity) as [A10] becomes Infinity(0) (On the asymmetric branches)

In this scenario, [A01] and [A10] each inidivually saturate towards an unique asymptotic value.

Scenario 1 : Asymptotes (If [A01] & [A10] is robust, [A00] & [A11] becomes 0/infinite)

Since [A00] or [A11] becomes infinite, we are interested in solutions of the kind, epsilon = 0/infinity

1. [A11] = infinity, [A00] = 0, epsilon = 0

simplify(*eval*(*eval*(*simplify*(*A11*)), epsilon = 0))

$$\frac{\left(-A10\left(\left(\left(\left(-c_1-c_3\right)k_4+c_1k_1\right)k_2+k_4c_3k_3\right)c_2-k_4c_3k_3\right)c_4-c_1c_2k_1k_2}{\left(A10\left(k_1-k_4\right)c_4+k_1\right)c_2c_1c_3\left(k_2-k_3\right)} \quad \textbf{(3.2.1)}$$

This value is infinite - so the denominator must be equal to zero. This gives us the asymptotic value of [A10] as shown below

A10 = *simplify*(*solve*(*denom*(*simplify*(*eval*(*eval*(*simplify*(*A11*)), epsilon = 0))), *A10*))

$$A10=-\frac{k_1}{c_4\left(k_1-k_4\right)} \quad \textbf{(3.2.2)}$$

At this value, we can evaluate the other concentrations, and so we have the second asymptote for [A01].

$$simplify\left(eval\left(eval(A01, \text{epsilon}=0), A10=-\frac{k_1}{c_4\left(k_1-k_4\right)}\right)\right)$$

$$\frac{k_3}{\left(k_2-k_3\right)c_2} \quad \textbf{(3.2.3)}$$

1. [A11] = 0, [A00] = Infinity, epsilon = infinity

simplify(*A00*)

$$-\frac{\left(\left(A10\left(c_1k_1k_2-k_4\left(\left(c_1+c_3\right)k_2-c_3k_3\right)\right)c_4+c_1k_1k_2\right)c_2+c_3c_4k_3k_4\right)\epsilon}{\left(\left(k_2-k_3\right)\left(A10\left(k_1-k_4\right)(\epsilon+1)c_4+k_1\right)c_2-\epsilon c_4k_3\left(k_1-k_4\right)\right)c_3c_1} \quad \textbf{(3.2.4)}$$

This value is infinite - so the denominator must be equal to zero. This gives us the asymptotic value of [A10] as shown below

simplify(*limit*(*solve*(*denom*(*A00*), *A10*), epsilon = infinity))

$$\frac{k_3}{c_2\left(k_2-k_3\right)} \quad \textbf{(3.2.5)}$$

At this value, we can evaluate the other concentrations, and so we have the second asymptote for [A01].

$$simplify\left(eval\left(limit((A01), \text{epsilon} = \text{infinity}), A10 = \frac{k_3}{c_2\,(k_2 - k_3)}\right)\right)$$

$$-\frac{k_1}{c_4\,(k_1 - k_4)} \tag{3.2.6}$$

Thus for symmetry to break and lead to robustness in A10 and A10, the necessary condition is $k_2 > k_3$ and $k_4 > k_1$ and these are the respective asymptotes

Scenario 2 : Asymptotes (If [A00] & [A11] is robust, [A01] & [A10] becomes 0/infinite)

Since [A01] or [A10] becomes infinite, we are interested in solutions of the kind, = 0 and epsilon = infinity

1. [A01] = infinity, [A10] = 0

$$simplify(A01)$$

$$\left(\left(A10\, c_4\, (c_3\, \epsilon - c_1)\, k_4 + c_1\, k_1\, (A10\, c_4 - \epsilon + 1)\right) c_2\, k_2 - c_3\, c_4\, k_3\, k_4\, (A10\, c_2\, \epsilon + \epsilon - 1)\right) \Big/ \left(\left(\left((-c_1\, \epsilon + c_3)\, k_4 + \epsilon\, c_1\, k_1\right) k_2 - k_4\, c_3\, k_3\right) c_2\, c_4\right) \tag{3.3.1}$$

This value is infinite - so the denominator must be equal to zero. This gives us the asymptotic value of epsilon as shown below

$$\text{epsilon} = solve(denom(A01), \text{epsilon})$$

$$\epsilon = -\frac{c_3\, k_4\, (k_2 - k_3)}{k_2\, (k_1 - k_4)\, c_1} \tag{3.3.2}$$

At this value, we can evaluate the other concentrations and thus we have our two invariants

$$simplify\left(eval\left(simplify(A11), \epsilon = -\frac{c_3\, k_4\, (k_2 - k_3)}{k_2\, (k_1 - k_4)\, c_1}\right)\right)$$

$$-\frac{k_2}{c_3\, (k_2 - k_3)} \tag{3.3.3}$$

$$simplify\left(eval\left(simplify(A00), \epsilon = -\frac{c_3\, k_4\, (k_2 - k_3)}{k_2\, (k_1 - k_4)\, c_1}\right)\right)$$

$$\frac{k_4}{c_1\, (k_1 - k_4)} \tag{3.3.4}$$

2. [A01] = 0, [A10] = Infinity

$simplify(A01)$

$$\left(c_2\left(A10\, c_4\left(\epsilon\, c_3 - c_1\right) k_4 + c_1\, k_1\left(A10\, c_4 - \epsilon + 1\right)\right) k_2 - c_3\, c_4\, k_3\, k_4\left(A10\, c_2\, \epsilon + \epsilon - 1\right)\right) \Big/ \left(c_4\, c_2\left(\left(\left(-\epsilon\, c_1 + c_3\right) k_4 + \epsilon\, c_1\, k_1\right) k_2 - k_4\, c_3\, k_3\right)\right) \quad (3.3.5)$$

This value is infinite - so the denominator must be equal to zero. This gives us the asymptotic value of epsilon as shown below, where [A10] = infinity

$\text{epsilon} = simplify(limit(solve(A01, \text{epsilon}), A10 = \text{infinity}))$

$$\epsilon = -\frac{c_1\left(k_1 - k_4\right) k_2}{c_3\, k_4\left(k_2 - k_3\right)} \quad (3.3.6)$$

At this value, we can evaluate the other concentrations and thus we have our two invariants

$$limit\left(simplify\left(eval\left(simplify(A11), \epsilon = -\frac{c_1\left(k_1 - k_4\right) k_2}{c_3\, k_4\left(k_2 - k_3\right)}\right)\right), A10 = \text{infinity}\right)$$

$$\frac{k_4}{\left(k_1 - k_4\right) c_1} \quad (3.3.7)$$

$$limit\left(simplify\left(eval\left(simplify(A00), \epsilon = -\frac{c_1\left(k_1 - k_4\right) k_2}{c_3\, k_4\left(k_2 - k_3\right)}\right)\right), A10 = \text{infinity}\right)$$

$$-\frac{k_2}{\left(k_2 - k_3\right) c_3} \quad (3.3.8)$$

Thus for symmetry to break and lead to robustness in A00 and A11, the necessary condition is $k_3 > k_2$ and $k_1 > k_4$ and these are the respective asymptotes

Mixed Random 1 DSP : Common Kinase Common Phosphatase Case 2 - Present and Doesn't Break

In this Maple file we analytically show the infeasibility of case 2 symmetry breaking in the Mixed Random 1 DSP network with common kinase and common phosphatase effecting distributive random phosphorylation and random processive dephoshorylation respectively. We do this by first describing the model as a system of ODEs along with the associated enzyme and substrate conservations. We then impose the kinetic constraints pertinent to case 2 symmetry. By solving for the steady state of the system of ODEs we obtain relations between concentrations of the substrate variables in terms of each other and the free enzyme concentrations. After this, we identify key symmetric pairings that represent the symmetric steady state. i.e. case 2 symmetry breaking requires symmetry between [A01] & [A10]; By leveraging this insight, we show the infeasibility of case 2 symmetry breaking by revealing that [A01] and [A10] are always equal for any given feasible steady state. These procedures are carried out in detail below using built in Maple commands.

Note: A subscript is used to distinguish between the two different complexes formed between [K] and [A00]. Similarly a subscript is used to distinguish between the two distinct complexes formed between [P] and [A11].

We initialize the Maple file with the *restart* command and load the relevant libraries of inbuilt Maple functions (*LinearAlgebra, VectorCalculus, Student[LinearAlgebra]*)

$restart : with(VectorCalculus) : with(LinearAlgebra) : with(Student[LinearAlgebra]) :$

The system is modelled as a set of ODEs using the kinetic nomenclature described in the main text and supplementary figure (refer Appendix 2 figure 10). Here dA00 represents d[A00]/dt and similarly for other expressions. At steady state thus, each of the right hand sides of these expressions will be equal to zero.

$$dA00 := -k_{b1}\, A00\cdot K - a_{b1}\, A00\cdot K + k_{ub1}\cdot A00K_1 + a_{ub1}\cdot A00K_2 + a_4\cdot A10P + k_4\cdot A01P :$$
$$dA01 := -k_{b2}\, A01\cdot K + k_1\, A00K_1 + k_{ub2}\, A01K :$$
$$dA10 := -a_{b2}\, A10\cdot K + a_1\, A00K_2 + a_{ub2}\, A10K :$$
$$dA11 := k_2\, A01K + a_2\, A10K - k_{b3}\cdot A11\cdot P - a_{b3}\cdot A11\cdot P + k_{ub3}\cdot A11P_1 + a_{ub3}\cdot A11P_2 :$$

$$dA00K_1 := k_{b1}\cdot A00\cdot K - (k_1 + k_{ub1})\cdot A00K_1 :$$
$$dA10K := a_{b2}\cdot A10\cdot K - (a_2 + a_{ub2})\cdot A10K :$$
$$dA00K_2 := a_{b1}\cdot A00\cdot K - (a_{ub1} + a_1)\cdot A00K_2 :$$
$$dA01K := k_{b2}\cdot A01\cdot K - (k_{ub2} + k_2)\cdot A01K :$$
$$dA11P_1 := k_{b3}\cdot A11\cdot P - (k_{ub3} + k_3)\cdot A11P_1 :$$
$$dA10P := a_3\cdot A11P_2 - a_4\cdot A10P :$$
$$dA11P_2 := a_{b3}\cdot A11\cdot P - (a_{ub3} + a_3)\cdot A11P_2 :$$
$$dA01P := k_3\cdot A11P_1 - k_4\cdot A01P :$$

$$dK := -k_{b1}\cdot A00\cdot K + (k_1 + k_{ub1})\cdot A00K_1 - a_{b2}\cdot A10\cdot K + (a_2 + a_{ub2})\cdot A10K - a_{b1}\cdot A00\cdot K + (a_{ub1}$$

$+ a_1)\cdot A00K_2 - k_{b2}\cdot A01\cdot K + (k_{ub2} + k_2)\cdot A01K$:

$dP := -k_{b3}\cdot A11\cdot P + k_{ub3}\cdot A11P_1 + a_4\cdot A10P - a_{b3}\cdot A11\cdot P + a_{ub3}\cdot A11P_2 + k_4\cdot A01P$:

The above equations are also associated with conservation conditions which are described below. Here we store the conservation expressions as ACon, PCon and KCon for the substrate and the respective enzymes. Each of these expressions is always equal to zero (both in the transient behavior and at steady state).

$ACon := A_{Total} - A00 - A10 - A01 - A11 - A00K_1 - A01K - A00K_2 - A10K - A11P_1 - A10P - A11P_2 - A01P$:

$KCon := K_{Total} - K - A00K_1 - A10K - A00K_2 - A01K$:

$PCon := P_{Total} - P - A11P_1 - A10P - A11P_2 - A01P$:

Proof for impossibility of symmetry breaking

Kinetic constraints for case 2 symmetry (refer main text) are imposed on the original model.

$a_1 := k_1 : a_{b1} := k_{b1} : a_{ub1} := k_{ub1}$:

$a_2 := k_2 : a_{b2} := k_{b2} : a_{ub2} := k_{ub2}$:

$a_3 := k_3 : a_{b3} := k_{b3} : a_{ub3} := k_{ub3}$:

$a_4 := k_4$:

There are no constraints on the total enzyme concentrations for case 2 symmetry to be present in Mixed Random 1 DSP. At this stage we introduce auxiliary constants c_1, c_2, and c_3 in place of the binding constants so as to make further analytical expressions more accessible.

$k_{b1} := c_1\cdot(k_{ub1} + k_1)$:

$k_{b2} := c_2\cdot(k_{ub2} + k_2)$:

$k_{b3} := c_3\cdot(k_{ub3} + k_3)$:

Once this is done, we solve for the steady state of the system in terms of fewer key variables. In this context we want to solve all variables in terms of the concentrations of the free enzymes ([K] & [P]) and the concentrations of the fully unmodified substrate [A00] and the fully modified substrate [A11]. In order to do this, we use the Maple command *solve* which solves the equation supplied for a given variable. We first solve for the individual complexes using their corresponding differential equation. An example of this (using [$A00K_1$] is given below in detail).

The differential equation of [$A00K_1$] is given by,

$$\frac{d\,[A00K_1]}{dt} = dA00K_1 \qquad \frac{d\,[A00K_1]}{dt} = c_1\,(k_1 + k_{ub1})\,A00\,K - (k_1 + k_{ub1})\,A00K_1$$

The *solve* command by Maple, solves this equation for the given variable (in this case [$A00K_1$]). We in turn store this value (the solution returned by the *solve* command) in [$A00K_1$]. This is performed by the following command.

$A00K_1 := solve(dA00K_1, A00K_1)$

$$A00K_1 := K\, A00\, c_1 \quad (1.1)$$

This operation is performed for the other complexes and substrate forms as well. Here we simultaneously solve expressions for the substrate forms using the *solve* command as shown below. The solution is stored in a variable labelled *Sol*, and then the respective solutions are extracted from this vector using the *eval* command.

$A01K := solve(dA01K, A01K) :$
$A00K_2 := solve(dA00K_2, A00K_2) :$
$A10K := solve(dA10K, A10K) :$
$A11P_1 := solve(dA11P_1, A11P_1) :$
$A10P := solve(dA10P, A10P) :$
$A11P_2 := solve(dA11P_2, A11P_2) :$
$A01P := solve(dA01P, A01P) :$

$S := solve(\{dA10, dA01\}, \{A01, A10\}) :$

$A00 := eval(A00, S) :$
$A01 := eval(A01, S) :$
$A10 := eval(A10, S) :$
$A11 := eval(A11, S) :$

Doing this results in the following correlations between the concentrations of the various substrate forms at steady state.

$A00 = A00$

$$A01 = \frac{A00\, c_1\, k_1}{c_2\, k_2}$$

$$A10 = \frac{A00\, c_1\, k_1}{c_2\, k_2}$$

$A11 = A11$

Proof for impossibility of symmetry breaking

Thus from this we can clearly see that irrespective of kinetic parameters, the concentration of [A01] is always going to be equal to the concentration of [A10]. Thus there is no scope for any asymmetric steady state or case 2 symmetry breaking.

$$A01 = \frac{A00\ c_1\ k_1}{c_2\ k_2}$$

$$A10 = \frac{A00\ c_1\ k_1}{c_2\ k_2}$$

Mixed Random 2 DSP : Separate Kinase Common Phosphatase Case 2 - Present and Breaks

In this Maple file we analytically show the presence of case 2 symmetry breaking in the Mixed Random 2 DSP network with separate kinase and common phosphatase effecting distributive random phosphorylation and processive dephoshorylation respectively. We do this by first describing the model as a system of ODEs along with the associated substrate and enzyme conservations. We then impose the kinetic constraints pertaining to case 2 symmetry. By solving for the steady state of the system of ODEs we obtain relations between concentrations of the substrate variables in terms of each other and the free enzyme concentrations. After this, we identify key symmetric pairings that represent the symmetric steady state. i.e. case 2 symmetry breaking requires symmetry between [A01] & [A10] and [K1] & [K2]. By leveraging this insight and isolating steady states not of this type, we ascertain the features of the asymmetric steady state emerging from symmetry breaking. These procedures are carried out in detail below using built in Maple commands.

Note: A subscript is used to distinguish between the two different complexes formed between [P] and [A11].

We initialize the Maple file with the *restart* command and load the relevant libraries of inbuilt Maple functions (*LinearAlgebra, VectorCalculus, Student[LinearAlgebra]*)

$restart : with(LinearAlgebra) : with(VectorCalculus) : with(Student[LinearAlgebra]) :$

The system is modelled as a set of ODEs using the kinetic nomenclature described in the main text and supplementary figure (refer Appendix 2 figure 10). Here dA00 represents d[A00]/dt and similarly for other expressions. At steady state thus, each of the right hand sides of these expressions will be equal to zero.

$dA00 := -k_{b1}\, A00 \cdot K1 - a_{b1}\, A00 \cdot K2 + k_{ub1} \cdot A00K1 + a_{ub1} \cdot A00K2 + a_4 \cdot A10P + k_4 \cdot A01P :$

$dA01 := -k_{b2}\, A01 \cdot K2 + k_1\, A00K1 + k_{ub2}\, A01K2 :$

$dA10 := -a_{b2}\, A10 \cdot K1 + a_1\, A00K2 + a_{ub2}\, A10K1 :$

$dA11 := k_2 \cdot A01K2 + a_2 \cdot A10K1 - k_{b3} \cdot A11 \cdot P - a_{b3} \cdot A11 \cdot P + k_{ub3} \cdot A11P_1 + a_{ub3} \cdot A11P_2 :$

$dA00K1 := k_{b1} \cdot A00 \cdot K1 - (k_1 + k_{ub1}) \cdot A00K1 :$

$dA10K1 := a_{b2} \cdot A10 \cdot K1 - (a_2 + a_{ub2}) \cdot A10K1 :$

$dA00K2 := a_{b1} \cdot A00 \cdot K2 - (a_{ub1} + a_1) \cdot A00K2 :$

$dA01K2 := k_{b2} \cdot A01 \cdot K2 - (k_{ub2} + k_2) \cdot A01K2 :$

$dA11P_1 := k_{b3} \cdot A11 \cdot P - (k_{ub3} + k_3) \cdot A11P_1 :$

$dA10P := a_3 \cdot A11P_2 - a_4 \cdot A10P :$

$dA11P_2 := a_{b3} \cdot A11 \cdot P - (a_{ub3} + a_3) \cdot A11P_2 :$

$dA01P := k_3 \cdot A11P_1 - k_4 \cdot A01P :$

$dK1 := -k_{b1} \cdot A00 \cdot K1 + (k_1 + k_{ub1}) \cdot A00K1 - a_{b2} \cdot A10 \cdot K1 + (a_2 + a_{ub2}) \cdot A10K1 :$

$dK2 := -a_{b1} \cdot A00 \cdot K2 + (a_{ub1} + a_1) \cdot A00K2 - k_{b2} \cdot A01 \cdot K2 + (k_{ub2} + k_2) \cdot A01K2 :$

$dP := -k_{b3} \cdot A11 \cdot P + k_{ub3} \cdot A11P_1 + a_4 \cdot A10P - a_{b3} \cdot A11 \cdot P + a_{ub3} \cdot A11P_2 + k_4 \cdot A01P :$

The model is also associated with conservation conditions for the substrate and enzyme concentrations which are described below. Here we store the conservation expressions in ACon, K1Con, K2Con and PCon for the substrate and respective enzymes. The right hand side of each of these expressions are always equal to zero (both in the transient behavior and at steady state).

$$ACon := A_{Total} - A00 - A10 - A01 - A11 - A00K1 - A01K2 - A00K2 - A10K1 - A11P_1 - A10P - A11P_2 - A01P :$$

$$K1Con := K1_{Total} - K1 - A00K1 - A10K1 :$$

$$K2Con := K1_{Total} - K2 - A00K2 - A01K2 :$$

$$PCon := P_{Total} - P - A11P_1 - A10P - A11P_2 - A01P :$$

Kinetic constraints for case 2 symmetry (refer main text) are imposed on the original model.

$$a_1 := k_1 : a_{b1} := k_{b1} : a_{ub1} := k_{ub1} :$$

$$a_2 := k_2 : a_{b2} := k_{b2} : a_{ub2} := k_{ub2} :$$

$$a_3 := k_3 : a_{b3} := k_{b3} : a_{ub3} := k_{ub3} :$$

$$a_4 := k_4 :$$

In addition to the kinetic constraints the total enzyme concentrations of the two kinases need to be equal for exact case 2 symmetry to be present in Mixed-Random 2 DSP. This is imposed as shown below.

$$K2_{Total} := K1_{Total} :$$

At this stage we introduce auxiliary constants c_1, c_2, and c_3 in place of the binding constants so as to make further analytical expressions more accessible.

$$k_{b1} := c_1 \cdot (k_{ub1} + k_1) :$$

$$k_{b2} := c_2 \cdot (k_{ub2} + k_2) :$$

$$k_{b3} := c_3 \cdot (k_{ub3} + k_3) :$$

Once this is done, we solve for the steady state of the system in terms of fewer key variables. In this context we want to solve all variables in terms of the free enzymes ([K1], [K2] & [P]) and the partially modified substrate [A01]. In order to do this, we use the Maple command *solve* which solves the equation supplied for a given variable. We first solve for the individual complexes using their corresponding differential equation. An example of this (using [A00K1] is given below in detail).

The differential equation of [A00K1] is given by,

$$\frac{d\,[A00K1]}{dt} = dA00K1$$

$$\frac{d\,[A00K1]}{dt} = c_1\,(k_1 + k_{ub1})\,A00\,K1 - (k_1 + k_{ub1})\,A00K1 \quad (1)$$

The *solve* command by Maple, solves this equation for the given variable (in this case [A00K1]). We in turn store this value (the solution returned by the *solve* command) in [A00K1]. This is performed by the following command.

$A00K1 := solve(dA00K1, A00K1)$

$$A00K1 := K1\,A00\,c_1 \quad (2)$$

This operation is performed for the other complexes and substrate forms as well. Here we simultaneously solve expressions for the substrate forms using the *solve* command as shown below. The solution is stored in a variable labelled *Sol*, and then the respective solutions are extracted from this vector using the *eval* command.

$A01K2 := solve(dA01K2, A01K2)$:
$A00K2 := solve(dA00K2, A00K2)$:
$A10K1 := solve(dA10K1, A10K1)$:
$A11P_1 := solve(dA11P_1, A11P_1)$:
$A10P := solve(dA10P, A10P)$:
$A11P_2 := solve(dA11P_2, A11P_2)$:
$A01P := solve(dA01P, A01P)$:

$S := solve(\{dA00, dA11, dA10\}, \{A00, A11, A10\})$:

$A00 := eval(A00, S)$:
$A01 := eval(A01, S)$:
$A10 := eval(A10, S)$:
$A11 := eval(A11, S)$:

Doing this results in the following correlations between the concentrations of the various substrate forms at steady state.

$$A00 = \frac{K2\,A01\,c_2\,k_2}{K1\,c_1\,k_1}$$

$$A01 = A01$$

$$A10 = \frac{K2^2\,A01}{K1^2}$$

$$A11 = \frac{K2\,A01\,c_2\,k_2\,(K1 + K2)}{2\,K1\,P\,c_3\,k_3}$$

Proof for invariant in the asymmetric branches

We know that K1Con and K2Con are both individually equal to zero always. Thus at a given

steady state, K1Con - K2Con must also be equal to zero.

We thus introduce the term T = (K1Con - K2Con) = 0 and also introduce a new ratio, ϵ = [K1]/[K2]. Note: As discussed in the main text, the symmetric steady state is one where [K1] = [K2] or ϵ = 1. Since we are isolating solutions of asymmetry, we are primarily interested in solutions that permit, ϵ ≠ 1.

$T := K1Con - K2Con = 0:$
$K1 := \epsilon \cdot K2:$

The following command (*simplify*), simplifies the expression algebraically

$$simplify(T) = \frac{\left(-\epsilon k_1 + A01\, c_2 \left(k_1 - k_2\right)\right) K2\, (\epsilon - 1)}{k_1\, \epsilon} = 0$$

From this we can ascertain that, should an asymmetric steady state exist (where ϵ ≠ 1) - the term ($-\epsilon k_1 + A01\, c_2 \left(k_1 - k_2\right)$) in the expression needs to necessarily be zero. This term is an expression in the partial substrate form [A01], ϵ and kinetic constants. Thus solving this to isolate the partially modified substrate form we get the following. Here we use the *solve* command from Maple to solve T for [A01] and the *simplify* command to algebraically simplify the resulting expression.

$A01 := simplify(solve(T, A01))$

$$A01 := \frac{\epsilon k_1}{\left(k_1 - k_2\right) c_2} \tag{1.1}$$

Substituting this value for [A01] it back into the expression for the concentration of [A01], we get the following correlation.

$$A00 = \frac{k_2}{\left(k_1 - k_2\right) c_1}$$

We note that the concentration of [A00] is fixed in the asymmetric steady state and is given by a few key kinetic parameters. Using this information (the concentration of [A00] at an asymmetric steady state) - we solve for [K2], [P] at this asymmetric steady state using their individual enzyme conservation equations. This is done by finding solutions for [K2] and [P] using equations K2Con and PCon as shown below, using the Maple command *solve.*

$P := simplify(solve(PCon, P)):$
$K2 := simplify(solve(K2Con, K2)):$

$$simplify(P) = \frac{-k_2 \left(k_3 + k_4\right) K1_{Total} + P_{Total}\, k_3\, k_4}{k_3\, k_4}$$

$simplify(K2) =$

$$\frac{K1_{Total}\,(k_1 - k_2)}{k_1\,(\epsilon + 1)}$$

Using these expressions, we find that the concentration of [A11] and [P] is also fixed and is given by only a few kinetic constants and total enzyme concentrations as shown below.

$$simplify(P) = \frac{-k_2\,(k_3 + k_4)\,K1_{Total} + P_{Total}\,k_3\,k_4}{k_3\,k_4}$$

$$simplify(A11) = -\frac{k_2\,k_4\,K1_{Total}}{2\,c_3\,\left(k_2\,(k_3 + k_4)\,K1_{Total} - P_{Total}\,k_3\,k_4\right)}$$

Necessary conditions

Since substrate concentration are always necessarily positive, the expression for [A00] and [A11] above must be positive. The numerators are only a function of kinetic parameters which are always positive, thus the denominator must necessarily be positive to ensure that the resulting concentration is positive. This gives us the necessary condition for an asymmetric state to exist as follows.

1. $k_2 < k_1$

2. $k_2\,K1_{total}\,(k_3 + k_4) < P_{total}\,k_3\,k_4$

Sufficiency of necessary conditions

In this section of the proof we show the necessary conditions shown above are also sufficient for an asymmetric steady state to exist for some positive A_{Total} value. i.e. We show that upon a bifurcation along A_{Total} we are bound to encounter symmetry breaking provided the necessary conditions are satisfied. Note that a feasible steady state in this context is one in which the concentrations of all substrates, complexes and enzymes are positive.

We do this by showing that the asymmetric states defined by the concentration (invariant) of [A00], [A11] & [P] described above is indeed a feasible solution for the system of ODEs at some positive A_{Total} value.

In an asymmetric steady state, as seen above the concentration of [A00], [A11] & [P] are fixed by a few kinetic constants and total enzyme concentrations,

$$A00 := \frac{k_2}{(k_1 - k_2)\,c_1} = \frac{k_2}{(k_1 - k_2)\,c_1}$$

$$A11 := -\frac{k_2\,k_4\,K1_{Total}}{2\,c_3\,\left(k_2\,(k_3 + k_4)\,K1_{Total} - P_{Total}\,k_3\,k_4\right)} =$$

$$-\frac{1}{2}\frac{k_2 k_4 K1_{Total}}{c_3 \left(k_2 \left(k_3+k_4\right) K1_{Total} - P_{Total} k_3 k_4\right)}$$

$$P := \frac{-k_2 \left(k_3+k_4\right) K1_{Total} + P_{Total} k_3 k_4}{k_3 k_4} = \frac{-k_2 \left(k_3+k_4\right) K1_{Total} + P_{Total} k_3 k_4}{k_3 k_4}$$

The other variables in this asymmetric state are thus given by

$$A00 = \frac{k_2}{\left(k_1 - k_2\right) c_1}$$

$$A11 = -\frac{k_2 k_4 K1_{Total}}{2 c_3 \left(k_2 \left(k_3+k_4\right) K1_{Total} - P_{Total} k_3 k_4\right)}$$

$$A00K1 = \frac{\epsilon K1_{Total} k_2}{k_1 (\epsilon + 1)}$$

$$A00K2 = \frac{K1_{Total} k_2}{k_1 (\epsilon + 1)}$$

$$A01K2 = \frac{K1_{Total} \epsilon}{\epsilon + 1}$$

$$A10K1 = \frac{K1_{Total}}{\epsilon + 1}$$

$$K1 = \frac{\epsilon K1_{Total} \left(k_1 - k_2\right)}{k_1 (\epsilon + 1)}$$

$$K2 = \frac{K1_{Total} \left(k_1 - k_2\right)}{k_1 (\epsilon + 1)}$$

$$simplify\left(A11P_1\right) = \frac{K1_{Total} k_2}{2 k_3}$$

$$simplify\left(A11P_2\right) = \frac{K1_{Total} k_2}{2 k_3}$$

$$simplify(A01P) = \frac{K1_{Total} k_2}{2 k_4}$$

$$simplify(A10P) = \frac{K1_{Total} k_2}{2 k_4}$$

The system of ODE is also satisfied at this point, as is verified below.

$simplify(dA00) = 0$
$simplify(dA11) = 0$
$simplify(dA01) = 0$
$simplify(dA10) = 0$

$simplify(dA00K1) = 0$
$simplify(dA00K2) = 0$
$simplify(dA01P) = 0$
$simplify(dA10P) = 0$
$simplify(dA01K2) = 0$
$simplify(dA10K1) = 0$
$simplify(dA11P_1) = 0$
$simplify(dA11P_2) = 0$
$simplify(dP) = 0$
$simplify(dK1) = 0$
$simplify(dK2) = 0$

Hence all that remains to be shown is that the variables (As described above) are positive for some value of A_{Total}.
This is true if and only if

1. **Necessary condition $(k_3 > k_1)$ is satisfied**
2. **ϵ is positive.**

However if ϵ is positive all concentrations are automatically positive (provided necessary conditions are satisfied).

Thus this means that for every positive value of $\epsilon \neq 1$, all concentrations are positive and the conservation of kinase and phosphatase is also satisfied.

Since the concentrations are all positive there exists a unique finite A_{Total} value for every ϵ (permitting asymmetric states).

Hence we have proved that symmetry breaking is guaranteed for some finite positive A_{Total}, provided the necessary conditions above are satisfied - making those conditions sufficient for the behavior.

Prediction of pitchfork bifurcation along A_{Total}

Here we predict the value of A_{Total} at which symmetry breaking occurs via a pitchfork bifurcation. This point in the bifurcation is characterized by the intersection of both the symmetric steady state branch and the asymmetric steady state branches.

Hence at the position of symmetry breaking, we know two insights.

1. The system is still symmetric, hence [A01] = [A10] and [K1] = [K2].
2. The invariant describing the asymmetric steady state is also true.

Using these two information, we can simplify the original system considerably as follows.

$K1 := K2 : \epsilon := 1 :$

$$A00 := \frac{k_2}{(k_1 - k_2)\, c_1} :$$

$$A11 := -\frac{k_2\, k_4\, K1_{Total}}{2\, c_3\, \left(k_2\, (k_3 + k_4)\, K1_{Total} - P_{Total}\, k_3\, k_4\right)} :$$

$$P := \frac{-k_2\, (k_3 + k_4)\, K1_{Total} + P_{Total}\, k_3\, k_4}{k_3\, k_4} :$$

Now, by solving the conservation expression for the substrate we can isolate the value of A_{Total} when the asymmetric steady states and the symmetric steady state intersect (indicating the pitchfork bifurcation point)

$$A_{Total} = simplify\left(solve\left(simplify(ACon), A_{Total}\right)\right)$$

$$A_{Total} = \Bigg(-2\, (k_3 + k_4)\, c_3\, c_1\, K1_{Total}^{\ 2}\, \left((k_1 + k_4)\, k_3 + k_1\, k_4\right) c_2\, k_2^{\ 3} + 2\, \Bigg(c_3\, \big(c_1\, P_{Total}\, k_4^{\ 2} + k_1\, (c_1\, P_{Total} + 1)\, k_4 + c_1\, k_1^{\ 2}\, K1_{Total} \big)\, k_3^{\ 2} + 2\, k_1\, k_4\, \Bigg(\left(\left(\frac{1}{2}\, c_1\, c_3\, P_{Total} + \frac{1}{4}\, c_1 + \frac{1}{2}\, c_3 \right) k_4 + c_1\, c_3\, k_1\, K1_{Total} \right) k_3 + c_1\, c_3\, k_1^{\ 2}\, k_4^{\ 2}\, K1_{Total} \Bigg) \Bigg) K1_{Total}\, c_2\, k_2^{\ 2} - 2\, k_3\, k_1\, k_4\, \Bigg(c_3\, \big(c_2\, P_{Total}\, k_4 + c_1\, k_1\, K1_{Total}\, (-K1_{Total}\, c_2 + c_2\, P_{Total} - 2) \big)\, k_3 + c_1\, k_1\, k_4\, K1_{Total} \left(c_2\, c_3\, P_{Total} - c_2\, c_3\, K1_{Total} + \frac{1}{2}\, c_2 - 2\, c_3 \right) \Bigg) k_2 - 2\, c_1\, c_3\, k_1^{\ 2}\, k_3^{\ 2}\, k_4^{\ 2}\, P_{Total}\, (K1_{Total}\, c_2 + 2) \Bigg) \Bigg/ \Big(2\, k_3\, (k_1 - k_2)\, k_1\, c_3\, \left(k_2\, (k_3 + k_4)\, K1_{Total} - P_{Total}\, k_3\, k_4\right) c_1\, k_4\, c_2 \Big) \qquad \textbf{(4.1)}$$

A cross verification of this analytical work is carried out in the read me file for the parameters used in generating the figures (Fig 2E).

Mixed Random 2a DSP : Separate Kinase Common Phosphatase Case 2 - Present and Breaks

In this Maple file we analytically show the presence of case 2 symmetry breaking in the Mixed Random 2a DSP network with separate kinase effecting distributive random phosphorylation and unsaturated processive dephoshorylation respectively (modelled as a linear reaction). We do this by first describing the model as a system of ODEs along with the associated substrate and enzyme conservations. We then impose the kinetic constraints pertaining to case 2 symmetry. By solving for the steady state of the system of ODEs we obtain relations between concentrations of the substrate variables in terms of each other and the free enzyme concentrations. After this, we identify key symmetric pairings that represent the symmetric steady state. i.e. case 2 symmetry breaking requires symmetry between [A01] & [A10] and [K1] & [K2]. By leveraging this insight and isolating steady states not of this type, we ascertain the features of the asymmetric steady state emerging from symmetry breaking. These procedures are carried out in detail below using built in Maple commands.

Note: A subscript is used to distinguish between the two different complexes formed between [P] and [A11].

We initialize the Maple file with the *restart* command and load the relevant libraries of inbuilt Maple functions (*LinearAlgebra, VectorCalculus, Student[LinearAlgebra]*)

$restart : with(LinearAlgebra) : with(VectorCalculus) : with(Student[LinearAlgebra]) :$

The system is modelled as a set of ODEs using the kinetic nomenclature described in the main text and supplementary figure (refer Appendix 2 figure 10). Here dA00 represents d[A00]/dt and similarly for other expressions. At steady state thus, each of the right hand sides of these expressions will be equal to zero.

$$dA00 := -k_{b1}\,A00\,K1 - p_{b1}\,A00\,K2 + k_{ub1}\cdot A00K1 + p_{ub1}\cdot A00K2 + k_3\cdot A11 + p_3\cdot A11 :$$
$$dA01 := -k_{b2}\,A01\,K2 + k_1\,A00K1 + k_{ub2}\,A01K2 :$$
$$dA10 := -p_{b2}\,A10\,K1 + p_1\,A00K2 + p_{ub2}\,A10K1 :$$
$$dA11 := k_2\,A01K2 + p_2\,A10K1 - k_3\cdot A11 - p_3\cdot A11 :$$

$$dA00K1 := k_{b1}\cdot A00\cdot K1 - (k_1 + k_{ub1})\cdot A00K1 :$$
$$dA10K1 := p_{b2}\cdot A10\cdot K1 - (p_2 + p_{ub2})\cdot A10K1 :$$
$$dA00K2 := p_{b1}\cdot A00\cdot K2 - (p_{ub1} + p_1)\cdot A00K2 :$$
$$dA01K2 := k_{b2}\cdot A01\cdot K2 - (k_{ub2} + k_2)\cdot A01K2 :$$

$$dK1 := -k_{b1}\cdot A00\cdot K1 + (k_1 + k_{ub1})\cdot A00K1 - p_{b2}\cdot A10\cdot K1 + (p_2 + p_{ub2})\cdot A10K1 :$$
$$dK2 := -p_{b1}\cdot A00\cdot K2 + (p_{ub1} + p_1)\cdot A00K2 - k_{b2}\cdot A01\cdot K2 + (k_{ub2} + k_2)\cdot A01K2 :$$

The above equations are associated with conservation conditions which are described below. Here we store the conservation expressions in ACon, K1Con and K2Con for the substrate and the respective enzymes. The right hand side of each of these expressions are always equal to zero (both in the transient behavior and at steady state).

$ACon := A_{Total} - A00 - A10 - A01 - A11 - A00K1 - A01K2 - A00K2 - A10K1 :$
$K1Con := K1_{Total} - K1 - A00K1 - A10K1 :$
$K2Con := K1_{Total} - K2 - A00K2 - A01K2 :$

Kinetic constraints for case 2 symmetry (refer main text) are imposed on the original model.

$p_1 := k_1 : p_{b1} := k_{b1} : p_{ub1} := k_{ub1} :$
$p_2 := k_2 : p_{b2} := k_{b2} : p_{ub2} := k_{ub2} :$
$p_3 := k_3 :$

In addition to the kinetic constraints the total enzyme concentrations of the two kinases need to be equal for exact case 2 symmetry to be present in Mixed-Random 3 DSP. This is imposed as shown below.

$K2_{Total} := K1_{Total} :$

At this stage we introduce auxiliary constants c_1, c_2, and c_3 in place of the binding constants so as to make further analytical expressions more accessible.

$k_{b1} := c_1 \cdot (k_{ub1} + k_1) :$
$k_{b2} := c_2 \cdot (k_{ub2} + k_2) :$

Once this is done, we solve for the steady state of the system in terms of fewer key variables. In this context we want to solve all variables in terms of the free enzymes ([K1], [K2] & [P]) and the partially modified substrate [A01]. In order to do this, we use the Maple command *solve* which solves the equation supplied for a given variable. We first solve for the individual complexes using their corresponding differential equation. An example of this (using [A00K1] is given below in detail).

The differential equation of [A00K1] is given by,

$$\frac{d\,[A00K1]}{dt} = dA00K1 \qquad \frac{d\,[A00K1]}{dt} = c_1\,(k_1 + k_{ub1})\,A00\,K1 - (k_1 + k_{ub1})\,A00K1$$

The *solve* command by Maple, solves this equation for the given variable (in this case [A00K1]). We in turn store this value (the solution returned by the *solve* command) in [A00K1]. This is performed by the following command.

$A00K1 := solve(dA00K1, A00K1)$

$$A00K1 := K1\,A00\,c_1 \qquad (1)$$

This operation is performed for the other complexes and substrate forms as well. Here we simultaneously solve expressions for the substrate forms using the *solve* command as shown below. The solution is stored in a variable labelled *Sol*, and then the respective solutions are

extracted from this vector using the *eval* command.

$A01K2 := solve(dA01K2, A01K2) :$
$A00K2 := solve(dA00K2, A00K2) :$
$A10K1 := solve(dA10K1, A10K1) :$

$Sol := solve(\{dA00, dA11, dA10\}, \{A00, A11, A10\}) :$

$A00 := eval(A00, Sol) :$
$A01 := eval(A01, Sol) :$
$A10 := eval(A10, Sol) :$
$A11 := eval(A11, Sol) :$

Doing this results in the following correlations between the concentrations of the various substrate forms at steady state.

$$A00 = \frac{A01\ K2\ c_2\ k_2}{K1\ c_1\ k_1}$$

$$A01 = A01$$

$$A10 = \frac{A01\ K2^2}{K1^2}$$

$$A11 = \frac{A01\ K2\ c_2\ k_2\ (K1 + K2)}{2\ K1\ k_3}$$

Proof for invariant in asymmetric branches

We know that K1Con and K2Con are both individually equal to zero always. Thus at a given steady state, K1Con - K2Con must also be equal to zero.

We thus introduce the term T = (K1Con - K2Con) = 0 and also introduce a new ratio, ϵ = [K1]/[K2]. Note: As discussed in the main text, the symmetric steady state is one where [K1] = [K2] or ϵ = 1. Since we are isolating solutions of asymmetry, we are primarily interested in solutions that permit, $\epsilon \neq 1$.

$T := K1Con - K2Con = 0 :$
$K1 := \epsilon \cdot K2 :$

The following command (*simplify*), simplifies the expression

$$simplify(T) = \frac{K2\ \left(-\epsilon\ k_1 + A01\ c_2\ \left(k_1 - k_2\right)\right)\ (\epsilon - 1)}{k_1\ \epsilon} = 0$$

From this we can ascertain that, should an asymmetric steady state exist (where $\epsilon \neq 1$) - the term ($-\epsilon\ k_1 + A01\ c_2\ \left(k_1 - k_2\right)$) in the expression needs to necessarily be zero. This term is an expression in the partial substrate form [A01], ϵ and kinetic constants. Thus solving this to

isolate the partially modified substrate form in terms of the kinetic parameters we get the following. Here we use the *solve* command from Maple to solve T for [A01]. We use the *simplify* command to algebraically simplify the resulting expression.

$A01 := simplify(solve(T, A01))$

$$A01 := \frac{\epsilon k_1}{(k_1 - k_2)\, c_2} \quad \textbf{(1.1)}$$

Substituting this value for [A01] it back into the expression for the concentration of [A01], we get the following correlation.

$$A00 = \frac{k_2}{(k_1 - k_2)\, c_1}$$

We note that the concentration of [A00] is fixed in the asymmetric steady state and is given by a few key kinetic parameters. Using this information (the concentration of [A00] at an asymmetric steady state) - we solve for the concentration of free enzyme [K2] at this asymmetric steady state using the corresponding enzyme conservation equation (K2Con). This operation is carried out using the Maple command *solve* as shown below.

$K2 := simplify(solve(K2Con, K2)) :$

$$K2 = \frac{K1_{Total}\,(k_1 - k_2)}{k_1\,(\epsilon + 1)}$$

Using these expressions, we find that the concentration of [A11] is also fixed and given by only a few key kinetic constants and total enzyme concentrations as shown below.

$$simplify(A11) = \frac{K1_{Total}\, k_2}{2\, k_3}$$

Necessary conditions

Since substrate concentration are always necessarily positive, the expression for [A00] and [A11] above must be positive. The numerators are only a function of kinetic parameters which are always positive, thus the denominator must be positive to ensure that the resulting concentration is positive. This gives us the necessary condition for an asymmetric state to exist as follows.

1. $k_2 < k_1$

Sufficiency of necessary conditions

In this section of the proof we show the necessary conditions shown above are also sufficient

for an asymmetric steady state to exist for some positive A_{Total} value. i.e. We show that upon a bifurcation along A_{Total} we are bound to encounter symmetry breaking provided the necessary conditions are satisfied. Note that a feasible steady state in this context is one in which the concentrations of all substrates, complexes and enzymes are positive.

We do this by showing that the asymmetric states defined by the invariant concentration of [A00] & [A11] described above is indeed a feasible solution for the system of ODEs at some positive A_{Total} value.

In an asymmetric steady state, as seen above the concentration of [A00] and [A11] are fixed by a few kinetic constants and total enzyme concentrations,

$$A00 := \frac{k_2}{(k_1 - k_2)\, c_1} = \frac{k_2}{(k_1 - k_2)\, c_1}$$

$$A11 := \frac{K1_{Total}\, k_2}{2\, k_3} = \frac{1}{2}\, \frac{K1_{Total}\, k_2}{k_3}$$

The other variables in this asymmetric state are thus given by

$$A00 = \frac{k_2}{(k_1 - k_2)\, c_1}$$

$$A11 = \frac{K1_{Total}\, k_2}{2\, k_3}$$

$$A00K1 = \frac{\epsilon\, K1_{Total}\, k_2}{k_1\, (\epsilon + 1)}$$

$$A00K2 = \frac{K1_{Total}\, k_2}{k_1\, (\epsilon + 1)}$$

$$A01K2 = \frac{K1_{Total}\, \epsilon}{\epsilon + 1}$$

$$A10K1 = \frac{K1_{Total}}{\epsilon + 1}$$

$simplify(A11P_1) = A11P_1$
$simplify(A11P_2) = A11P_2$
$simplify(A01P) = A01P$
$simplify(A10P) = A10P$

The system of ODE is also satisfied at this point, as is verified below.

$simplify(dA00) = 0$
$simplify(dA11) = 0$
$simplify(dA01) = 0$
$simplify(dA10) = 0$

$simplify(dA00K1) = 0$
$simplify(dA00K2) = 0$
$simplify(dA01K2) = 0$
$simplify(dA10K1) = 0$
$simplify(dK1) = 0$
$simplify(dK2) = 0$

Hence all that remains to be shown is that the variables (As described above) are positive for some value of A_{Total}.
This is true if and only if

1. **Necessary condition $(k_1 > k_2)$ is satisfied**
2. **ϵ is positive.**

However if ϵ is positive all concentrations are automatically positive (provided necessary conditions are satisfied).

Thus this means that for every non singular value of ϵ all concentrations are positive and the conservation of kinase and phosphatase is also satisfied.

Since the concentrations are all positive there exists a unique A_{Total} value for every ϵ (permitting asymmetric states).

Hence we have proved that symmetry breaking is guaranteed for some finite positive A_{Total}, provided the necessary conditions above are satisfied - making those conditions sufficient for the behavior.

Prediction of pitchfork bifurcation along A_{Total}

Here we predict the value of A_{Total} at which symmetry breaking occurs via a pitch fork bifurcation. This point in the bifurcation is characterized by the intersection of both the symmetric steady state branch and the asymmetric steady state branches.

Hence at the position of symmetry breaking, we know two insights.

1. The system is still symmetric, hence [A01] = [A10] and [K1] = [K2].
2. The invariant describing the asymmetric steady state is also true.

Using these two information, we can simplify the original system considerably as follows.

$K1 := K2 : \epsilon := 1 :$

$$A00 := \frac{k_2}{(k_1 - k_2)\, c_1} :$$

$$A11 := \frac{K1_{Total}\, k_2}{2\, k_3} :$$

Now, by solving the conservation expression for the substrate we can isolate the value of A_{Total} when the asymmetric steady states and the symmetric steady state intersect (indicating the pitchfork bifurcation point)

$$A_{Total} = simplify\left(solve\left(simplify(ACon), A_{Total}\right)\right)$$

$$A_{Total} = \frac{1}{2\left(k_1 - k_2\right) c_1 c_2 k_3 k_1}\left(c_1\left(K1_{Total}\left(k_2 + 2k_3\right) c_2 + 4k_3\right) k_1^2 - \left(K1_{Total} k_2 c_1 - 2k_3\right) k_2 c_2 k_1 - 2K1_{Total} k_2^2 c_1 c_2 k_3\right) \quad \textbf{(4.1)}$$

A cross verification of this analytical work is carried out in the read me file for the parameters used in generating the figures (Appendix 2 Fig 1).

Absolute (exact) concentration robustness
Ordered DSP - Common kinase common phosphatase

In this maple file, we analytically show three key results pertaining to the presence of (exact) ACR in the ordered DSP system with common kinase common phosphotase effecting phosphorylation and dephosphorylation respectively (without any imposition of symmetry at the kinetic level). Namely,

1. We show that among the substrates, only the partially modified form (Ap) can show ACR in the system irrespective of kinetic parameters; i.e. The compeltely modified or completely unmodified substrate form is incapable of exhibiting ACR. This ACR is again only possible with changing total amounts of substrate concentration (A_{Total}).
2. The presence of ACR is (if present) guaranteed on two distinct branches of steady states (for any given A_{Total}) value.
3. If an ACR branch is to be present for some A_{Total}, then there will always be a non-ACR branch on which the ratio of the free kinase to free phosphatase is exactly constant irrespective of total substrate concentrations.

The ACR considered here is defined here as follows -- "If the substrate form is robust (maintained at an exact concentration) to increasing (or changing) total concentration of either the substrate or the enzymes (A_{Total}/K_{Total}/P_{Total}), that branch of steady state is termed to be an ACR branch, and the substrate form that shows robustness is said to exhibit ACR"

Put together these results provide the insight that granular symmetry is not strictly required in the network for ACR to be present - and other constratints involving kinetic constants and total concentrations of enzymes can guarentee ACR. Further discussion on the relevance of these results is provided in the appendix of the manuscript.

This section of the maple document pertaining to these results is structured with a common text introducing the model and initializing the steady state calculations - followed by different sub-sections each containing the proof to a specific self contained result. When running the code - please take notice to only run one sub-section at a time.

We begin by initializing the Maple file with the *restart* command and load the relevant libraries of inbuilt Maple functions (*LinearAlgebra, VectorCalculus, Student[LinearAlgebra]*)

$restart : with(LinearAlgebra) : with(VectorCalculus) : with(Student[LinearAlgebra]) :$

The system is modelled as a set of ODEs using the kinetic nomenclature described in the main text and supplementary figure (refer to Appendix 2 Figure 10). Here dA represents d[A]/dt and similarly in the case of the other variables. At steady state thus, the right hand sides of each of these expressions will be equal to zero.

$$dA := k_4 \cdot ApP + k_{ub1} \cdot AK - k_{b1} \cdot A \cdot K :$$
$$dAp := k_1 \cdot AK + k_3 \cdot AppP + k_{ub2} \cdot ApK + k_{ub4} \cdot ApP - k_{b2} \cdot Ap \cdot K - k_{b4} \cdot Ap \cdot P :$$
$$dApp := k_2 \cdot ApK + k_{ub3} \cdot AppP - k_{b3} \cdot App \cdot P :$$

$$dAK := k_{b1} \cdot A \cdot K - (k_{ub1} + k_1) \cdot AK :$$
$$dApK := k_{b2} \cdot Ap \cdot K - (k_{ub2} + k_2) \cdot ApK :$$
$$dAppP := k_{b3} \cdot App \cdot P - (k_{ub3} + k_3) \cdot AppP :$$
$$dApP := k_{b4} \cdot Ap \cdot P - (k_{ub4} + k_4) \cdot ApP :$$

$$dK := -k_{b1} \cdot A \cdot K + (k_{ub1} + k_1) \cdot AK - k_{b2} \cdot Ap \cdot K + (k_{ub2} + k_2) \cdot ApK :$$
$$dP := -k_{b3} \cdot App \cdot P + (k_{ub3} + k_3) \cdot AppP - k_{b4} \cdot Ap \cdot P + (k_{ub4} + k_4) \cdot ApP :$$

The model is also associated with conservation conditions which are described below. Here we store the conservation expressions as ACon, PCon and KCon for the substrate and the respective enzymes. Each of these expressions is always equal to zero (both in the transient behavior and at steady state).

$$ACon := A_{Total} - A - Ap - App - AK - ApK - AppP - ApP :$$
$$PCon := P_{Total} - P - AppP - ApP :$$
$$KCon := K_{Total} - K - AK - ApK :$$

$$k_{b1} := c_1 \cdot (k_1 + k_{ub1}) :$$
$$k_{b2} := c_2 \cdot (k_2 + k_{ub2}) :$$
$$k_{b3} := c_3 \cdot (k_3 + k_{ub3}) :$$
$$k_{b4} := c_4 \cdot (k_4 + k_{ub4}) :$$

Once this is done, we solve for the steady state of the system in terms of fewer key variables. In this context we want to solve all variables in terms of the concentrations of the free enzymes ([K] & [P]) and the concentration of the partially modified substrate ([Ap]). In order to do this, we use the Maple command *solve,* which solves the supplied equation for a given variable. We first solve for the individual complexes using their corresponding differential equation. An example of this

(using [AK]) is given below in detail.

$AK := solve(dAK, AK) = K\,A\,c_1$

This operation is repeated for the other complexes forms as well.

$ApK := solve(dApK, ApK)$:
$AppP := solve(dAppP, AppP)$:
$ApP := solve(dApP, ApP)$:

Here we introduce a new ratio ϵ which is equal to the concentration of free (unbound) kinase to the free (unbound) phosphotase.

$K := \epsilon \cdot P$:

We know that PCon is always equal to zero. Here we solve for the steady state concentration of the free phosphatase and in doing so we obtain an expression for it in terms of the ratio epsilion and the substrate forms.

$$P := solve(PCon, P) = \frac{P_{Total}}{Ap\,c_4 + App\,c_3 + 1}$$

As stated earlier - different sub-sections pertaining to specific proofs follow from here. Please take notice to run one sub-section at a time.

Proof of inability of the ACR in any substrate with changing K_{Total} (or P_{Total})

In this sub-section we show that no substrate is capable of exhibiting ACR for changing enzyme concentrations. In order to do this - we systematically show that the completely unmodified, partially modified and completely modified substrate forms are incapable of exhibiting ACR with increasing or changing K_{Total}.

A

We begin by solving the remaining equations to obtain expressions for substrates App, Ap at steady state in terms of concentrations of A and the ratio ϵ. We do this by using the inbuilt solve command in *Maple*.

$$Sol := solve(\{dAp, dApp\}, \{Ap, App\}) = \left\{ Ap = \frac{\epsilon\,A\,c_1\,k_1}{c_4\,k_4},\ App = \frac{\epsilon^2\,A\,c_1\,k_1\,c_2\,k_2}{c_4\,k_4\,c_3\,k_3} \right\}$$

$assign(Sol)$

Thus at steady state - analyzing the total conservation of the substrate using these expressions leads to (we have used the *numer* and *collect* command to isolate the numerator and reorganize the resulting A_{Total} expression respectively),

$collect(numer(-ACon), \epsilon)$

$$A^2 c_1^2 c_2^2 k_1^2 k_2^2 \epsilon^4 + \left(A^2 c_1^2 c_2 c_3 k_1^2 k_2 k_3 + A^2 c_1^2 c_2 c_4 k_1^2 k_2 k_3\right) \epsilon^3 + \left(A^2 c_1^2 c_3 c_4 k_1^2 k_3^2 + A^2 c_1 c_2 c_3 c_4 k_1 k_2 k_3 k_4 - A c_1 c_2 c_3 c_4 k_1 k_2 k_3 k_4 A_{Total} + A c_1 c_2 c_3 c_4 k_1 k_2 k_3 k_4 P_{Total} + A c_1 c_2 c_3 c_4 k_1 k_3^2 k_4 P_{Total} + A c_1 c_2 c_4 k_1 k_2 k_3 k_4\right) \epsilon^2 + \left(A^2 c_1 c_3 c_4^2 k_1 k_3^2 k_4 - A c_1 c_3 c_4^2 k_1 k_3^2 k_4 A_{Total} + A c_1 c_3 c_4^2 k_1 k_3^2 k_4 P_{Total} + A c_1 c_3 c_4^2 k_3^2 k_4^2 P_{Total} + A c_1 c_3 c_4 k_1 k_3^2 k_4\right) \epsilon + A c_3 c_4^2 k_3^2 k_4^2 - c_3 c_4^2 k_3^2 k_4^2 A_{Total} \quad (1.1.1)$$

Here we proceed by a proof of contradiction - if we assume that A exhibits ACR for a range of K_{Total} values - then this expression simplifies to simply a single variate polynomial in ϵ (roots of which represents steady state solutions of the system). The key insight here is that since this expression does not contain K_{Total}, changing K_{Total} values would not affect the roots of this polynomial, implying ϵ can only take certain values at steady states with ACR. This is a contradiction as the expression for K_{Total} cannot accomodate a non-changing A and ϵ with changing K_{Total}, as shown below.

$collect(numer(KCon), \epsilon)$

$$c_4 k_4 \left(A c_1 c_2 k_1 k_2 K_{Total} - A c_1 c_2 k_1 k_3 P_{Total}\right) \epsilon^2 + c_4 k_4 \left(A c_1 c_4 k_1 k_3 K_{Total} - A c_1 c_4 k_3 k_4 P_{Total} - c_4 k_3 k_4 P_{Total}\right) \epsilon + c_4^2 k_4^2 k_3 K_{Total} \quad (1.1.2)$$

i.e. By the assumption of ACR in A, it is fixed at a given value (by definition of ACR) for a range of K_{Total} values. Thus in the resulting polynomial from KCon, ϵ (feasible roots of the resulting polynomial in ϵ) will have to necessarily change as K_{Total} changes. However equation ACon above, which was independent of K_{Total}, has fixed roots of ϵ which are consequently independent of K_{Total}. As stated earlier the common feasible root of these two equations would define steady state concentrations of the system. Since the roots in ϵ change in equation KCon with changing K_{Total} and remain fixed in equation ACon - we have a contradiction. Thus A cannot be fixed exactly for a range of K_{Total}.

Thus A cannot exhibit ACR.

Ap

We begin by solving the remaining equations to obtain expressions for substrates A, App at steady state in terms of concentrations of Ap and the ratio ϵ. We do this by using the inbuilt

solve **command in** ***Maple.***

$$Sol := solve(\{dA, dApp\}, \{A, App\}) = \left\{A = \frac{Ap\, c_4\, k_4}{c_1\, \epsilon\, k_1}, App = \frac{\epsilon\, Ap\, c_2\, k_2}{c_3\, k_3}\right\}$$

$assign(Sol)$

Thus at steady state - analyzing the total conservation of the substrate using these expressions leads to (we have used the *numer* and *collect* command to isolate the numerator and reorganize the resulting A_{Total} expression respectively),

$collect(numer(-ACon), \epsilon)$

$$Ap^2\, c_1\, c_2^2\, \epsilon^3\, k_1\, k_2^2 + \left(Ap^2\, c_1\, c_2\, c_3\, k_1\, k_2\, k_3 + Ap^2\, c_1\, c_2\, c_4\, k_1\, k_2\, k_3 - Ap\, c_1\, c_2\, c_3\, k_1\, k_2\, k_3\, A_{Total} + Ap\, c_1\, c_2\, c_3\, k_1\, k_2\, k_3\, P_{Total} + Ap\, c_1\, c_2\, c_3\, k_1\, k_3^2\, P_{Total} + Ap\, c_1\, c_2\, k_1\, k_2\, k_3\right)\epsilon^2 + \left(Ap^2\, c_1\, c_3\, c_4\, k_1\, k_3^2 + Ap^2\, c_2\, c_3\, c_4\, k_2\, k_3\, k_4 - Ap\, c_1\, c_3\, c_4\, k_1\, k_3^2\, A_{Total} + Ap\, c_1\, c_3\, c_4\, k_1\, k_3^2\, P_{Total} + Ap\, c_1\, c_3\, c_4\, k_3^2\, k_4\, P_{Total} + Ap\, c_1\, c_3\, k_1\, k_3^2 - c_1\, c_3\, k_1\, k_3^2\, A_{Total}\right)\epsilon + Ap^2\, c_3\, c_4^2\, k_3^2\, k_4 + Ap\, c_3\, c_4\, k_3^2\, k_4 \quad (1.2.1)$$

Here we proceed by contradiction - if we assume that Ap exhibits ACR for a range of K_{Total} values - then this expression simplifies to simply a single variate polynomial in ϵ (roots of which represents steady state solutions of the system). The key insight here is that since this expression does not contain K_{Total}, changing K_{Total} values would not affect the roots of this expresssion, implying ϵ can only take certain values at steady states with ACR. This is a contradiction as the expression for K_{Total} cannot accomodate a non-changing Ap and ϵ with changing K_{Total}, as shown below

$collect(numer(KCon), \epsilon)$

$$\left(Ap\, c_2\, k_1\, k_2\, K_{Total} - Ap\, c_2\, k_1\, k_3\, P_{Total} - k_1\, k_3\, P_{Total}\right)\epsilon + Ap\, c_4\, k_1\, k_3\, K_{Total} - P_{Total}\, k_3\, Ap\, c_4\, k_4 + k_1\, k_3\, K_{Total} \quad (1.2.2)$$

i.e. By the assumption of ACR in Ap, it is fixed at a given value (by definition of ACR) for a range of K_{Total} values. Thus in the resulting polynomial from KCon, ϵ (feasible roots of the resulting polynomial in ϵ) will have to necessarily change as K_{Total} changes. However equation ACon above, which was independent of K_{Total}, has fixed roots of ϵ which are consequently independent of K_{Total}. As stated earlier the common feasible root of these two equations would define steady state concentrations of the system. Since the roots in ϵ change in equation KCon with changing K_{Total} and remain fixed in equation ACon - we have a contradiction. Thus Ap cannot be fixed exactly for a range of K_{Total}.

Thus Ap cannot exhibit ACR.

App

We begin by solving the remaining equations to obtain expressions for substrates A, Ap at steady state in terms of concentrations of App and the ratio ϵ. We do this by using the inbuilt solve command in *Maple*.

$$Sol := solve(\{dA, dAp\}, \{A, Ap\}) = \left\{A = \frac{App\, c_3\, c_4\, k_3\, k_4}{c_1\, c_2\, \epsilon^2\, k_1\, k_2}, Ap = \frac{App\, c_3\, k_3}{c_2\, \epsilon\, k_2}\right\}$$

$assign(Sol)$

Thus at steady state - analyzing the total conservation of the substrate using these expressions leads to (we have used the *numer* and *collect* command to isolate the numerator and reorganize the resulting A_{Total} expression respectively),

$collect(numer(-ACon), \epsilon)$

$$\left(App^2\, c_1\, c_2^2\, c_3\, k_1\, k_2^2 - App\, c_1\, c_2^2\, c_3\, k_1\, k_2^2\, A_{Total} + App\, c_1\, c_2^2\, c_3\, k_1\, k_2^2\, P_{Total} + App\, c_1\, c_2^2\, c_3\, k_1\, k_2\, k_3\, P_{Total} + App\, c_1\, c_2^2\, k_1\, k_2^2 - c_1\, c_2^2\, k_1\, k_2^2\, A_{Total}\right) \epsilon^3 + \left(App^2\, c_1\, c_2\, c_3^2\, k_1\, k_2\, k_3 + App^2\, c_1\, c_2\, c_3\, c_4\, k_1\, k_2\, k_3 - App\, c_1\, c_2\, c_3\, c_4\, k_1\, k_2\, k_3\, A_{Total} + App\, c_1\, c_2\, c_3\, c_4\, k_1\, k_2\, k_3\, P_{Total} + App\, c_1\, c_2\, c_3\, c_4\, k_2\, k_3\, k_4\, P_{Total} + App\, c_1\, c_2\, c_3\, k_1\, k_2\, k_3\right) \epsilon^2 + \left(App^2\, c_1\, c_3^2\, c_4\, k_1\, k_3^2 + App^2\, c_2\, c_3^2\, c_4\, k_2\, k_3\, k_4 + App\, c_2\, c_3\, c_4\, k_2\, k_3\, k_4\right) \epsilon + App^2\, c_3^2\, c_4^2\, k_3^2\, k_4 \tag{1.3.1}$$

Here we proceed by contradiction - if we assume that App exhibits ACR for a range of K_{Total} values - then this expression simplifies to simply a single variate cubic polynomial in ϵ (roots of which represents steady state solutions of the system). The key insight here is that since this expression does not contain K_{Total}, changing K_{Total} values would not affect the roots of this expresssion, implying ϵ can only take certain values at steady states with ACR. This is a contradiction as the expression for K_{Total} cannot accomodate a non-changing App and ϵ with changing K_{Total}, as shown below

$collect(numer(KCon), \epsilon)$

$$-\epsilon^3\, P_{Total}\, c_2^2\, k_2^2\, k_1 + c_2\, k_2\left(App\, c_2\, c_3\, k_1\, k_2\, K_{Total} - App\, c_2\, c_3\, k_1\, k_3\, P_{Total} + c_2\, k_1\, k_2\, K_{Total}\right) \epsilon^2 + c_2\, k_2\left(App\, c_3\, c_4\, k_1\, k_3\, K_{Total} - P_{Total}\, App\, c_3\, c_4\, k_3\, k_4\right) \epsilon \tag{1.3.2}$$

i.e. By the assumption of ACR in App, it is fixed at a given value (by definition of ACR) for a range of K_{Total} values. Thus in the resulting polynomial from KCon, ϵ (feasible roots of the resulting polynomial in ϵ) will have to necessarily change as K_{Total} changes. However equation ACon above, which was independent of K_{Total}, has fixed roots of ϵ which are consequently independent of K_{Total}. As stated earlier the common feasible root of these two

equations would define steady state concentrations of the system. Since the roots in ϵ change in equation KCon with changing K_{Total} and remain fixed in equation ACon - we have a contradiction. Thus App cannot be fixed exactly for a range of K_{Total}.

Thus App cannot exhibit ACR.

Due to the structure of the DSP network, K_{Total} (App) and P_{Total} (A) are behaviorly equivalent (acheivable by a simple transformation of variables) and thus by extension a similar proof (omitted here) will rule out the presence of ACR in any of the substrates with increasing P_{Total}.

Absence of ACR in the fully modified (and unmodified) substrate with increasing A_{Total}

In this sub-section we show that the fully modified and the fully unmodified substrate forms (A and App) are capable of exhibiting ACR with changing total substrate concentrations.

We begin by solving the remaining equations to obtain expressions for substrates A, Ap at steady state in terms of concentrations of App and the ratio ϵ. We do this by using the inbuilt solve command in *Maple.*

$Sol := solve(\{dA, dAp\}, \{A, Ap\}) =$

$$Sol := \left\{A = \frac{App\, c_3\, c_4\, k_3\, k_4}{c_1\, c_2\, \epsilon^2\, k_1\, k_2}, Ap = \frac{App\, c_3\, k_3}{c_2\, \epsilon\, k_2}\right\} \quad \textbf{(2.1)}$$

$assign(Sol)$

Thus at steady state - analyzing the total conservation of the substrate using these expressions leads to (we have used the *numer* and *collect* command to isolate the numerator and reorganize the resulting A_{Total} expression respectively),

$collect(numer(KCon), \epsilon)$

$$-\epsilon^3 P_{Total}\, c_2^2\, k_2^2\, k_1 + c_2\, k_2\left(App\, c_2\, c_3\, k_1\, k_2\, K_{Total} - App\, c_2\, c_3\, k_1\, k_3\, P_{Total} + c_2\, k_1\, k_2\, K_{Total}\right)\epsilon^2 + c_2\, k_2\left(App\, c_3\, c_4\, k_1\, k_3\, K_{Total} - P_{Total}\, App\, c_3\, c_4\, k_3\, k_4\right)\epsilon \quad \textbf{(2.2)}$$

Similar to the earlier proof, we proceed with a proof by contradiction. If we assume that App presents with ACR for a range of A_{Total} values. Then the above expression simplifies to a single variate polynomial in ϵ (the roots of which represents steady state solutions of the system). The key insight here is that since this expression does not contain A_{Total}, changing A_{Total} values would not affect the roots of this expresssion, implying ϵ can only take certain values at steady states with ACR. This is a contradiction as the expression for A_{Total} cannot accomodate a non-

changing App and ϵ with changing A_{Total}.

collect(*numer*(*ACon*), ϵ)

$$\left(-App^2 c_1 c_2^2 c_3 k_1 k_2^2 + App\, c_1 c_2^2 c_3 k_1 k_2^2 A_{Total} - App\, c_1 c_2^2 c_3 k_1 k_2^2 P_{Total} - App\, c_1 c_2^2 c_3 k_1 k_2 k_3 P_{Total} - App\, c_1 c_2^2 k_1 k_2^2 + c_1 c_2^2 k_1 k_2^2 A_{Total}\right)\epsilon^3 + \left(-App^2 c_1 c_2 c_3^2 k_1 k_2 k_3 - App^2 c_1 c_2 c_3 c_4 k_1 k_2 k_3 + App\, c_1 c_2 c_3 c_4 k_1 k_2 k_3 A_{Total} - App\, c_1 c_2 c_3 c_4 k_1 k_2 k_3 P_{Total} - App\, c_1 c_2 c_3 c_4 k_2 k_3 k_4 P_{Total} - App\, c_1 c_2 c_3 k_1 k_2 k_3\right)\epsilon^2 + \left(-App^2 c_1 c_3^2 c_4 k_1 k_3^2 - App^2 c_2 c_3^2 c_4 k_2 k_3 k_4 - App\, c_2 c_3 c_4 k_2 k_3 k_4\right)\epsilon - App^2 c_3^2 c_4^2 k_3^2 k_4 \tag{2.3}$$

Thus App cannot exhibit ACR.

Due to the structure of the DSP network A and App are topologically equivalent with respect to variation in A_{Total} concentrations. Thus by a similar proof (omitted here), A is incapable of exhibiting ACR with changing A_{Total}.

Presence and features of ACR in the partially modified substrate form with increasing A_{Total}

In the earlier sub-sections we have shown how ACR is not possible in A and App with changing A_{Total}. In this sub-section we show that the remaining substrate form Ap is capable of exhibiting ACR with changing A_{Total} and also elucidate the associated features of such the network and it's steady states when it accomodates ACR.

We begin by solving the remaining equations to obtain expressions for substrates A, App at steady state in terms of concentrations of App and the ratio ϵ. We do this by using the inbuilt solve command in *Maple.*

$$Sol := solve(\{dA, dApp\}, \{A, App\}) = \left\{A = \frac{Ap\, c_4 k_4}{c_1 \epsilon k_1}, App = \frac{\epsilon Ap\, c_2 k_2}{c_3 k_3}\right\}$$

assign(*Sol*)

Thus at steady state - analyzing the total conservation of the substrate using these expressions leads to (we have used the *numer* and *collect* command to isolate the numerator and reorganize the resulting A_{Total} expression respectively),

collect(*numer*(*KCon*), ϵ)

$$\left(Ap\, c_2\, k_1\, k_2\, K_{Total} - Ap\, c_2\, k_1\, k_3\, P_{Total} - k_1\, k_3\, P_{Total}\right) \epsilon + Ap\, c_4\, k_1\, k_3\, K_{Total} - P_{Total}\, k_3\, Ap\, c_4\, k_4 + k_1\, k_3\, K_{Total} \tag{3.1}$$

Now if Ap were to exhibit ACR with changing A_{Total}, the above expression which is independent of A_{Total}, should be satisfied for differing values of the ratio ϵ. This independence in ϵ can only be guarenteed when the coeffecient of ϵ and the constant term are both zero.

This condition will provide a strict requirement on the kinetics (going beyond the granular symmetry assumed in case 1 symmetry earlier) and will also establish the concentration of Ap as functions of the kinetics of the network on the ACR branch as follows.

$$Ap = solve\left(\left(Ap\, c_2\, k_1\, k_2\, K_{Total} - Ap\, c_2\, k_1\, k_3\, P_{Total} - k_1\, k_3\, P_{Total}\right), Ap\right) =$$

$$Ap = \frac{k_3\, P_{Total}}{c_2\, \left(k_2\, K_{Total} - k_3\, P_{Total}\right)}$$

$$Ap = solve\left(Ap\, c_4\, k_1\, k_3\, K_{Total} - P_{Total}\, k_3\, Ap\, c_4\, k_4 + k_1\, k_3\, K_{Total}, Ap\right) =$$

$$Ap = -\frac{k_1\, K_{Total}}{c_4\, \left(K_{Total}\, k_1 - k_4\, P_{Total}\right)}$$

The two expressions for Ap obtained above (as functions of kinetics and total enzyme amounts) must be strictly equal and positive (since Ap is strictly positive).

Note: We quickly note an insight here that with case 1 symmetry in the kinetics, these two expressions are equivalent, however without the granular symmetry of case 1, these two expressions can still be satisfied, by suitable choice of kinetic constants and total enzyme concentrations.

Taking this in to account - ACon = 0 (the conservation expression for the total substrate concentration) similarly simplifies to

$$collect(numer(ACon), \epsilon)$$

$$\begin{aligned} &-Ap^2\, c_1\, c_2^2\, k_1\, k_2^2\, \epsilon^3 + \left(-Ap^2\, c_1\, c_2\, c_3\, k_1\, k_2\, k_3 - Ap^2\, c_1\, c_2\, c_4\, k_1\, k_2\, k_3 \right.\\ &\quad + Ap\, c_1\, c_2\, c_3\, k_1\, k_2\, k_3\, A_{Total} - Ap\, c_1\, c_2\, c_3\, k_1\, k_2\, k_3\, P_{Total} - Ap\, c_1\, c_2\, c_3\, k_1\, k_3^2\, P_{Total} \\ &\quad \left. - Ap\, c_1\, c_2\, k_1\, k_2\, k_3\right) \epsilon^2 + \left(-Ap^2\, c_1\, c_3\, c_4\, k_1\, k_3^2 - Ap^2\, c_2\, c_3\, c_4\, k_2\, k_3\, k_4 \right.\\ &\quad + Ap\, c_1\, c_3\, c_4\, k_1\, k_3^2\, A_{Total} - Ap\, c_1\, c_3\, c_4\, k_1\, k_3^2\, P_{Total} - Ap\, c_1\, c_3\, c_4\, k_3^2\, k_4\, P_{Total} \\ &\quad \left. - Ap\, c_1\, c_3\, k_1\, k_3^2 + c_1\, c_3\, k_1\, k_3^2\, A_{Total}\right) \epsilon - Ap^2\, c_3\, c_4^2\, k_3^2\, k_4 - Ap\, c_3\, c_4\, k_3^2\, k_4 \end{aligned} \tag{3.2}$$

Thus at a fixed value for Ap (deteremined from the equations earlier) - the above equation becomes a single variate polynomial in ϵ.

We make two observations here,

1. The above polynomial in ϵ has necessarily a negative real root (ascertained by noting the signs of the leading coeffecient and the constant) for positive kinetic constants.
2. The product of roots of the polynomial is negative.

Thus, if there exists an ϵ positive root, signifying a distinct ACR steady state, satisfying the polynomial there will always exist another positive real root root signifying the presence of another ACR steady state. Thus if ACR is to be present - it is necessarily present on two such branches for any given A_{Total}.

"Hence we draw our first conclusion regarding the ACR steady states - that should there exist an ACR steady state for Ap, there are necesarily two such branches with changing A_{Total}"

Sufficiency of necessary conditions

Here, before proceeding to ascertain further features of the system while exhibiting ACR, we show that the necessary kinetic conditions obtained earlier are sufficient to obtain ACR in the system at some finite positive A_{Total}

We make two further observations at this stage,

1. That at sufficiently large ATotal concentrations, the sum of the roots of the polynomial (in ϵ, ACon = 0) earlier is postive.
2. The discriminant of the cubic polynomial is also positive at some finite but sufficiently large A_{Total} as shown below

$$coeff\left(simplify(discrim(numer(ACon), \epsilon)), A_{Total}, 4\right)$$

$$c_3^4\, k_1^4\, c_1^4\, (-Ap\, c_4 - 1)^2\, k_3^6\, k_2^2\, Ap^2\, c_2^2 \qquad \textbf{(3.1.1)}$$

The resulting polynomial for the discriminant is a quartic in A_{Total}. We isolate the leading coeffecient of this polynomial as shown above using the inbuilt Maple command *coeff.* Now since this expression is positive for all positive kinetic constants, the discriminant is itself positive at some finite but sufficiently large A_{Total} concentration.

Thus the above two observations conclude that at some sufficiently large A_{Total} the polynomial admits two ϵ roots, each denoting a steady state on an ACR branch.

We now prove the existence of an intersection of the non-ACR branch and ACR branch.

We proceed with a proof by contraditction. Assuming that no intersection exists - there is no

common ϵ root for both equation 3.1 and 3.2. We should note here that while Ap is not fixed at its ACR concentration on the non-ACR branch (by definition) the kinetic parameters should none the less satisfy the necessary constraint elucidated above for the presence of ACR in the network; i.e.,

$$\frac{k_3 P_{Total}}{c_2 (k_2 K_{Total} - k_3 P_{Total})} = -\frac{k_1 K_{Total}}{c_4 (K_{Total} k_1 - k_4 P_{Total})}$$

Inorder to establish this equivalency (in kinetic constants and total concentrations of enzymes), we solve for k1 from this equation. This is an artibitrary choice and this expression can be satisfied by solving for any of the other constants involved.

$$k_1 = simplify\left(solve\left(\frac{k_3 P_{Total}}{c_2 (k_2 K_{Total} - k_3 P_{Total})} = -\frac{k_1 K_{Total}}{c_4 (K_{Total} k_1 - k_4 P_{Total})}, k_1\right)\right)$$

$$k_1 := \frac{c_4 k_3 k_4 P_{Total}^{\ 2}}{\left(-k_3 (c_2 - c_4) P_{Total} + c_2 k_2 K_{Total}\right) K_{Total}} \quad (3.3)$$

With this algebraic manipulation, equation 3.1 (KCon=0) from earlier can be rewritten as shown below,

$$simplify\left(numer\left(eval\left(KCon, k_1 = simplify\left(solve\left(\frac{k_3 P_{Total}}{c_2 (k_2 K_{Total} - k_3 P_{Total})} = -\frac{k_1 K_{Total}}{c_4 (K_{Total} k_1 - k_4 P_{Total})}, k_1\right)\right)\right)\right)\right)$$

$$k_3 \left((-Ap\, c_2 - 1) k_3 P_{Total} + K_{Total} Ap\, c_2 k_2\right) (\epsilon P_{Total} - K_{Total}) \quad (3.4)$$

We can see the birth and characteristic of the ACR branches more clearly now. There are two possible ways in which this conservation can be satisfied for any steady state. Either we are on an ACR branch - in which case the expression $(-Ap\, c_2 - 1) k_3 P_{Total} + Ap\, c_2 k_2 K_{Total}$ is equal to 0 (as we have noted earlier for the ACR branch), or ϵ is fixed as $\frac{K_{Total}}{P_{Total}}$.

Thus if we isolate for just the non-ACR branch (where ϵ is fixed as $\frac{K_{Total}}{P_{Total}}$), we observe that the remaining conservation equation for the substrate simplifies to a univariate quadratic polynomial in Ap. In this instance we don't resubstitute the k1 as we did earlier - but allow it to be a variable as such for the sake of transparancy in the expression.

$$T := collect\left(numer\left(eval\left(ACon, \epsilon = \frac{K_{Total}}{P_{Total}}\right)\right), Ap\right)$$

$$T := \left(-c_1 c_2^2 k_1 k_2^2 K_{Total}^3 - c_1 c_2 c_3 k_1 k_2 k_3 K_{Total}^2 P_{Total} - c_1 c_2 c_4 k_1 k_2 k_3 K_{Total}^2 P_{Total} - c_1 c_3 c_4 k_1 k_3^2 K_{Total} P_{Total}^2 - c_2 c_3 c_4 k_2 k_3 k_4 K_{Total} P_{Total}^2 - c_3 c_4^2 k_3^2 k_4 P_{Total}^3\right) Ap^2 + \left(c_1 c_2 c_3 k_1 k_2 k_3 A_{Total} K_{Total}^2 P_{Total} - c_1 c_2 c_3 k_1 k_2 k_3 K_{Total}^2 P_{Total}^2 - c_1 c_2 c_3 k_1 k_3^2 K_{Total}^2 P_{Total}^2 + c_1 c_3 c_4 k_1 k_3^2 A_{Total} K_{Total} P_{Total}^2 - c_1 c_3 c_4 k_1 k_3^2 K_{Total} P_{Total}^3 - c_1 c_3 c_4 k_3^2 k_4 K_{Total} P_{Total}^3 - c_1 c_2 k_1 k_2 k_3 K_{Total}^2 P_{Total} - c_1 c_3 k_1 k_3^2 K_{Total} P_{Total}^2 - c_3 c_4 k_3^2 k_4 P_{Total}^3\right) Ap + c_1 c_3 k_1 k_3^2 A_{Total} K_{Total} P_{Total}^2 \quad (3.5)$$

This quadratic polynomial has necessarily only one positive solution for Ap for every single value of A_{Total} (This can be quickly verified by noting the sign of the coeffecients of the polynomial). The range of the feasible root of Ap, as A_{Total} changes, would represent the range of Ap on the non-ACR branch. Allowing A_{Total} to vary between 0 to +infinity, one can note that the range of Ap also continously changes from 0 to +infinity (monotonically since it's a quadratic polynomial).

At some point within this range - Ap reaches the ACR value (provided by the kinetic constants).

Thus this proves the existence of an intersection between an ACR branch and the non-ACR branch, and that the non-ACR branch in the system has the unique feature that the ratio of unbound kinase to phosphatase is exactly fixed by the ratio of the total amounts of kinase and phosphatase at all total substrate concentrations.
